# Elevated protein lactylation promotes immunosuppressive microenvironment and therapeutic resistance in pancreatic ductal adenocarcinoma

**DOI:** 10.1172/JCI187024

**Published:** 2025-01-30

**Authors:** Kang Sun, Xiaozhen Zhang, Jiatao Shi, Jinyan Huang, Sicheng Wang, Xiang Li, Haixiang Lin, Danyang Zhao, Mao Ye, Sirui Zhang, Li Qiu, Minqi Yang, Chuyang Liao, Lihong He, Mengyi Lao, Jinyuan Song, Na Lu, Yongtao Ji, Hanshen Yang, Lingyue Liu, Xinyuan Liu, Yan Chen, Shicheng Yao, Qianhe Xu, Jieru Lin, Yan Mao, Jingxing Zhou, Xiao Zhi, Ke Sun, Xiongbin Lu, Xueli Bai, Tingbo Liang

**Affiliations:** 1 Department of Hepatobiliary and Pancreatic Surgery, The First Affiliated Hospital, School of Medicine and; 2 Zhejiang Provincial Key Laboratory of Pancreatic Disease, The First Affiliated Hospital, School of Medicine, Zhejiang University, Hangzhou, Zhejiang, China.; 3 MOE Joint International Research Laboratory of Pancreatic Diseases, Hangzhou, China.; 4 Zhejiang Provincial Clinical Research Center for the Study of Hepatobiliary & Pancreatic Diseases, Zhejiang University, Hangzhou, China.; 5 Cancer Center, Zhejiang University, Hangzhou, China.

**Keywords:** Immunology, Oncology, Cancer, Cancer immunotherapy, Macrophages

## Abstract

Metabolic reprogramming shapes the tumor microenvironment (TME) and may lead to immunotherapy resistance in pancreatic ductal adenocarcinoma (PDAC). Elucidating the impact of pancreatic cancer cell metabolism in the TME is essential to therapeutic interventions. “Immune cold” PDAC is characterized by elevated lactate levels resulting from tumor cell metabolism, abundance of protumor macrophages, and reduced cytotoxic T cells in the TME. Analysis of fluorine-18 fluorodeoxyglucose (^18^F-FDG) uptake in patients showed that increased global protein lactylation in PDAC correlates with worse clinical outcomes in immunotherapy. Inhibition of lactate production in pancreatic tumors via glycolysis or mutant-KRAS inhibition reshaped the TME, thereby increasing their sensitivity to immune checkpoint blockade (ICB) therapy. In pancreatic tumor cells, lactate induces K63 lactylation of endosulfine α (ENSA-K63la), a crucial step that triggers STAT3/CCL2 signaling. Consequently, elevated CCL2 secreted by tumor cells facilitates tumor-associated macrophage (TAM) recruitment to the TME. High levels of lactate also drive transcriptional reprogramming in TAMs via ENSA-STAT3 signaling, promoting an immunosuppressive environment. Targeting ENSA-K63la or CCL2 enhances the efficacy of ICB therapy in murine and humanized pancreatic tumor models. In conclusion, elevated lactylation reshapes the TME and promotes immunotherapy resistance in PDAC. A therapeutic approach targeting ENSA-K63la or CCL2 has shown promise in sensitizing pancreatic cancer immunotherapy.

## Abstract 2

Pancreatic cancer metabolism produces a high level of lactate, which changes tumor immune features by protein lactylation and thus results in immunotherapy resistance.

## Graphical abstract

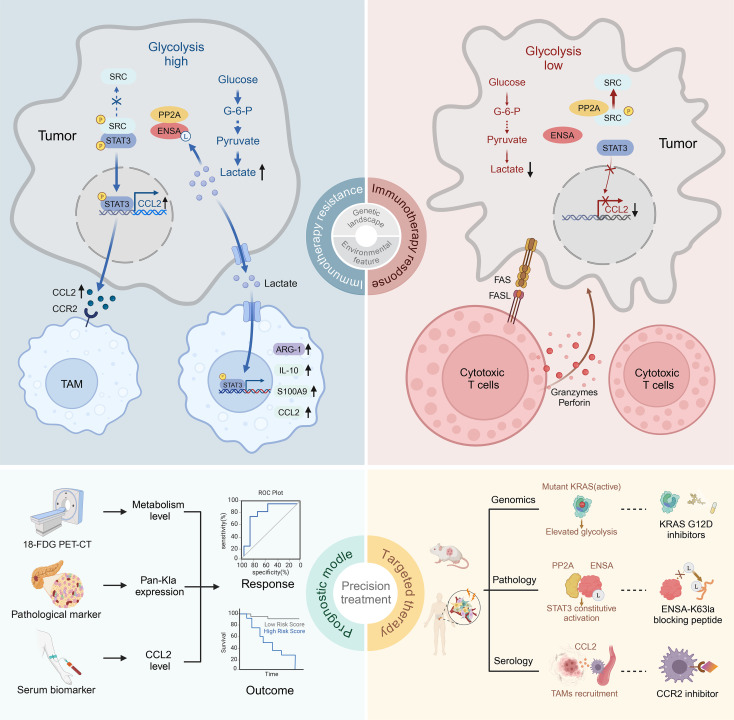

## Introduction

Pancreatic cancer remains a serious challenge in clinical practice, with 5-year survival rates very low at approximately 12% (1). Immunotherapy is considered promising for enabling long-term survival or recovery (2). However, in a randomized controlled trial (RCT) conducted at our center, while the combination use of immune checkpoint blockade (ICB) could improve the overall response rate (ORR), no clear survival benefit was observed and the benefit of adding PD-1 antibody sintilimab for advanced pancreatic cancer was not supported (3). A large portion of pancreatic cancer patients are resistant to immunotherapy.

During tumor initiation and progression, interactions among tumor cells, immunocytes, and stromal cells within the tumor microenvironment (TME) cause them to mutually influence each other. This dynamic interplay triggers a series of molecular biological changes that modify tumor metabolism and the microenvironment, culminating in the formation of a complex tumor ecosystem. This ecosystem collectively dictates treatment sensitivity (4). Tumor cells undergo metabolic reprogramming to gain growth advantages (5) driven by genetic mutations and environmental constraints (6, 7). Key somatic mutations such as KRAS, TP53, CDKN2A, and SMAD4 are prevalent in pancreatic cancer and impact metabolic processes (8, 9). Furthermore, pancreatic cancer progression is marked by vascular insufficiency, nutrient deficiency, hypoxia, and extracellular matrix alterations, which further contribute to metabolic shifts (10). Consequently, the metabolic profile of pancreatic cancer exhibits unique complexity and diversity compared with other malignancies, resulting in distinctive metabolic dependencies. Moreover, the heterogeneous genomic and environmental landscapes of pancreatic cancers contribute to tissue heterogeneity, leading to differential responses to therapeutic interventions. Therefore, a comprehensive exploration of the tissue, immune, metabolic, and molecular characteristics underlying resistance to immunotherapy in pancreatic cancer is essential. Identifying the pivotal drivers of resistance can facilitate precise interventions and enable the identification of patient subpopulations likely to benefit from immunotherapy, thereby enhancing treatment outcomes.

Pancreatic cancer is recognized for its high resistance to immune interventions, with a large portion of patients classified as immune exclusive or immune desert (2, 11). This poor immune profile often manifests with myeloid cell accumulation and sparse T cell presence, contributing to resistance against immunotherapies (12–14). Metabolic reprogramming plays a pivotal role in reshaping the immune microenvironment and is implicated in immunotherapy resistance (15). Analysis of data from The Cancer Genome Atlas– Pancreatic Adenocarcinoma (TCGA-PAAD) and Gene Expression Omnibus (GEO) databases revealed that “immune cold” pancreatic ductal adenocarcinoma (PDAC) is characterized by heightened glycolytic activity, which correlates positively with protumor macrophage infiltration and negatively with cytotoxic T cell infiltration. Clinically, patients exhibiting low fluorine-18 fluorodeoxyglucose (^18^F-FDG) uptake demonstrated improved responses to immunochemotherapy and prolonged progression-free survival (PFS). These findings underscore the impact of elevated glycolysis on immunotherapy resistance and underscore the need for identifying effective treatment modalities.

Lactate, as the end product of glycolysis, exerts a huge influence on the immune microenvironment (16). Studies indicate that elevated lactate levels can impair the migration and cytotoxic function of CD8^+^ T cells, while promoting polarization of macrophages toward a protumor phenotype and inhibiting monocyte differentiation into DCs. However, these observations are largely derived from in vitro investigations, and the precise role of lactate as a signaling molecule within the in vivo microenvironment remains ambiguous (17–19). Crucially, these studies have not accounted for spatial gradients in lactate distribution, spatial variations in immune cell infiltration, and the heterogeneous responses of immune cells to lactate. Therefore, further in vivo experiments are essential to elucidate how lactate remodels the immune microenvironment. Recent research has identified lactate as a versatile signaling molecule (2). Zhao et al. demonstrated that lysine lactylation contributes to epigenetic regulation (20). Lactylation has been implicated in regulating tissue homeostasis, metabolic transitions, tumor progression, and resistance to chemotherapy (20–23). Previous studies on lactylation have predominantly focused on histone lactylation. Yet, similarly to other acylation modifications, targeting histone lactylation may lead to unintended off-target effects (23, 24). Therefore, we are interested in nonhistone lactylation to identify critical proteins and specific lactylation sites for potential therapeutic intervention. In this study, we found that glycolysis-dependent ENSA-K63 lactylation inactivated PP2A, thereby sustaining SRC-S12 phosphorylation and STAT3-Y705 phosphorylation. Activation of STAT3 is known to enhance macrophage chemotaxis and promote protumor phenotypes. To counteract this pathway, we utilized a cell-penetrating peptide specific to ENSA-K63 lactylation, effectively blocking K63 lactylation and consequently inhibiting STAT3 activation in both tumor cells and macrophages. This strategy has shown promise in sensitizing immunotherapy approaches for pancreatic cancer.

## Results

### Elevated lactylation is correlated with immunosuppressive TME in PDAC.

**Figure 1:**
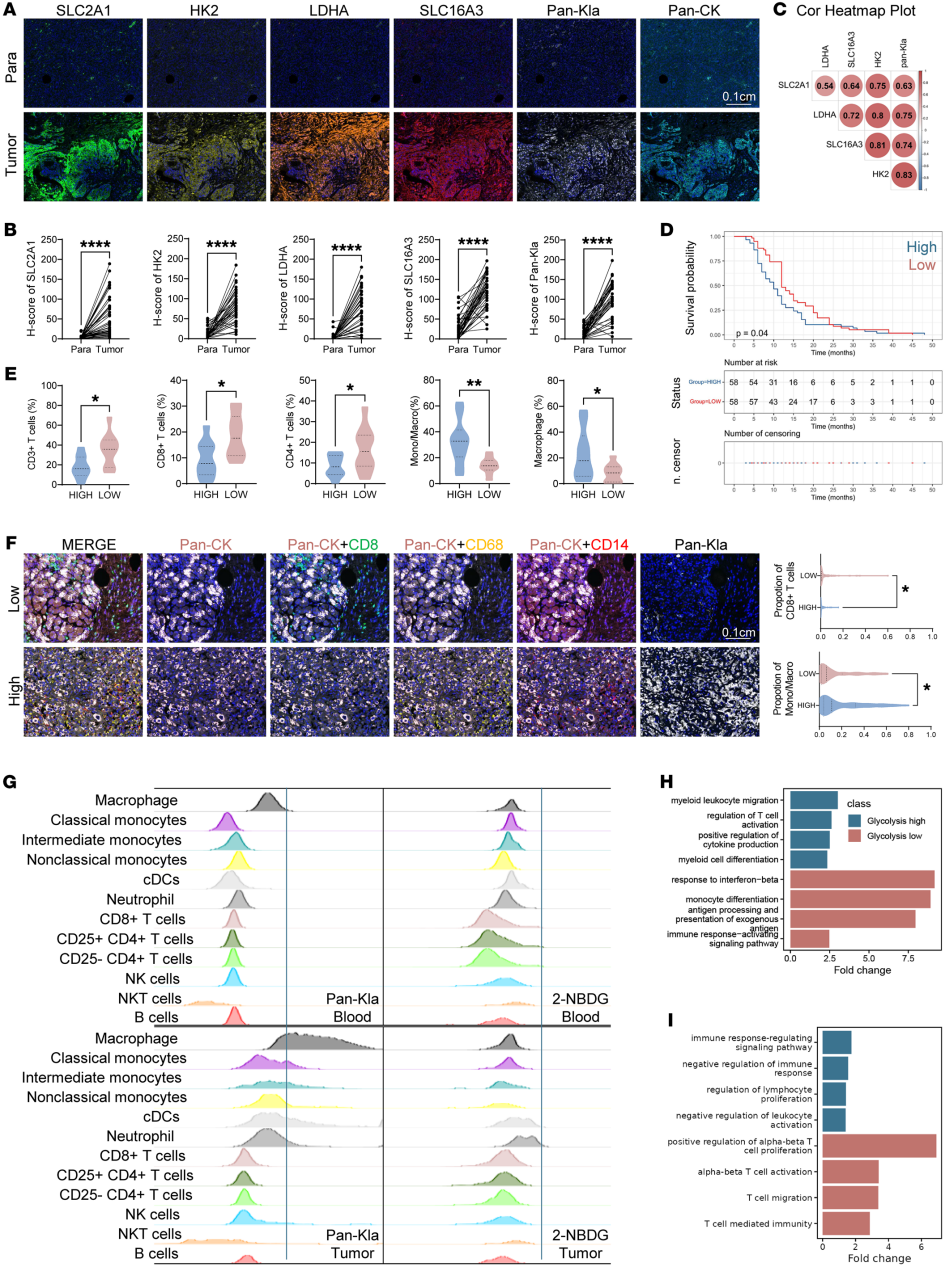
Elevated lactylation is correlated with immunosuppressive TME in PDAC. (**A**) Representative images of mIHC staining in paracancerous (Para) and tumor tissues. Paracancerous and tumor tissues on tissue microarray were stained. Scale bar: 0.1 cm. (**B**) Expression differences of indicated genes between paracancerous and tumor tissues. Histochemistry score (h-score) was used to measure the expression of these genes in each region. Intact tissues were included in follow-up analysis. Statistical analyses were performed with the paired sample *t* test (41 pairs). (**C**) Correlation analysis between SLC2A1, HK2, LDHA, SLC16A3, and Pan-Kla levels. Forty-one pairs of paracancerous and tumor tissues were included in the analysis. H-score was used to measure the expression of these genes in each region and then perform Pearson’s correlation analysis (*n* = 82). (**D**) Kaplan-Meier survival curves of overall survival (OS) for patients with PDAC in an in-house TMA. TMAs were stained using anti–Pan-Kla antibodies. Patients were divided into 2 groups according to median amount of h-score. Intact tumor tissues with survival data were included in follow-up analysis. Statistical analyses were performed with log-rank test (*n* = 116). (**E**) Twenty fresh pancreatic tumor samples were processed into a single-cell suspension and flow cytometry was performed using Cytek. Samples were divided into 2 groups according to Pan-Kla expression. Statistical analyses were performed with Student’s *t* test (*n* = 20). (**F**) Representative images and statistical chart of mIHC staining of pancreatic cancer samples. TMAs of 140 pancreatic cancer samples were stained. Intact tissues were included in follow-up analysis. Samples were divided into 2 groups according to h-score of Pan-Kla. Proportion of each cell type in total cells was calculated. Statistical analyses were performed with Student’s *t* test (*n* = 140). Scale bar: 0.1 cm. (**G**) Pan-Kla expression and 2-NBDG uptake tested using flow cytometry by each cell type in human paired tumors and peripheral blood. (**H** and **I**) Gene ontology enrichment analysis of macrophages (**H**) and CD8^+^ T cells (**I**). *P < 0.05; **P < 0.01; ****P < 0.0001.

As a metabolic waste and an end product from glycolysis, lactate was known to be associated with muscle fatigue and potential tissue harm (25). However, recent studies have unveiled a novel function of lactate: it can modify lysine residues on proteins, known as lysine lactylation (Kla or Klac). Recent studies have shown that lactylation promotes glycolysis in a feedback loop, sustaining the nuclear NAD^+^ salvage pathway, thereby driving oncogenesis and metastasis (26–28). However, the regulation of the immune microenvironment by lactylation in PDAC remains poorly understood. Using multiplex immunohistochemistry (mIHC), we observed that pan-lysine lactylation (Pan-Kla) levels were dramatically elevated in pancreatic tumor tissues compared with their paired paracancerous normal tissues (Figure 1, A and B). Increased levels of Pan-Kla modification indicated anaerobic glycolysis-related lactate accumulation in the tumor tissues. In the glycolysis pathway, SLC2A1 serves as the primary glucose transporter. Hexokinase 2 (HK2) acts as the rate-limiting enzyme responsible for converting glucose to glucose-6-phosphate, which is the first crucial step in this pathway. Lactate dehydrogenase A (LDHA) plays a pivotal role in converting pyruvate into lactate, while SLC16A3 functions as the transporter responsible for moving lactate into the TME (20, 29). We found that these key genes are all upregulated in the PDAC, and as expected, their expression levels are correlated well with Pan-Kla levels (Figure 1C and Supplemental Figure 1A; supplemental material available online with this article; https://doi.org/10.1172/JCI187024DS1). Pan-Kla level analysis using tissue microarrays demonstrated that elevated levels of Pan-Kla were correlated with worse overall survival (Figure 1D).

To study the impact of protein lactylation on the tumor immune microenvironment, we applied a 24-color high-dimensional immunoprofiling panel in spectral flow cytometry (CyTek) to identify the tumor-infiltrated immune cells in fresh PDAC tissue samples. A total of 20 resected pancreatic tumor tissues were divided into 2 groups according to their global lactylation levels (Supplemental Figure 1B and Supplemental Table 1). The group with high Pan-Kla levels exhibited a higher level of macrophage (CD11b^+^HLADR^+^CD68^+^) infiltration, but a lower level of infiltration for CD8^+^ T (CD3^+^CD4^-^CD8^+^) cells (Figure 1E and Supplemental Figure 1C). Next, we used an mIHC staining assay to validate the impact of lactylation on the tumor immune profiles with resectable PDAC using tissue microarrays. Consistently, the high Pan-Kla group showed higher infiltration of monocytes/macrophages (CD14^+^ or CD68^+^) and lower infiltration of CD8^+^ T cells (CD8^+^) in comparison with the low Pan-Kla group (Figure 1F). Collectively, elevated lactylation was associated with a more immunosuppressive TME in PDAC, characterized by macrophage accumulation and limited CD8^+^ T cell infiltration.

Lactate is produced by tumors and accumulates in the TME through SLC16A3. The concentration gradient of lactate influences the biological functions of cells near the tumor. Given that elevated lactylation impacts immune cell infiltration, we hypothesized that heterogeneous spatial distribution and different responsiveness to lactate could further reshape immune cell function. Among different cell types in the TME of patients with PDAC, lactylation was highest in macrophages (Figure 1G). Lactylation was significantly elevated in tumor-associated macrophages (TAMs) compared with macrophages in peripheral blood, supporting the idea that tumor cell metabolism reprograms macrophages in the TME (Figure 1G). 2-NBDG, a glucose analog, was used to detect glucose uptake and utilization both in vivo or in vitro (30). Compared with peripheral blood, glucose utilization in TAMs was not elevated in tumor tissue and did not constitute the majority of glucose utilization among immune cells (Figure 1G). This demonstrated that the elevated lactylation levels in TAMs were not derived from increased glycolysis activity, but likely from elevated lactate intake from the TME.

To further explore the impact of elevated lactate accumulation on infiltrated TAMs and CD8^+^ T cells at the single-cell level, we analyzed public single-cell RNA sequencing (scRNA-Seq) datasets of PDAC (Gene Expression Omnibus [GEO] GSE205013; National Genomics Data Center CRA001160; Human Tumor Atlas Network PHS002371; GEO GSE212966) (Supplemental Figure 1D) (31–34). Patient tumors were divided into 2 groups based on median glycolysis scores determined by the expression levels of glycolysis-related genes (SLC2A1, HK2, GPI1, PFKL, PGK, ALDOA, PGAM, GAPDH, PFKM, ENO1, PKM, LDHA, and SLC16A3) in tumor cells. Macrophages from tumors with high glycolysis scores underwent phenotype reprogramming, presenting as changes of transcriptional activity (such as NF-κB1, STAT1, JUN, and STAT3), elevating expression of protumor inflammation markers and immune checkpoints (IL1A, IL1B, IL10, S100A8, S100A9, CCL2, CCL4, CD274, NT5E), and alongside decreasing expression of genes associated with CD8^+^ T cell migration and activation (ICOS, CCL19, IFNG) (Figure 1H and Supplemental Figure 1, E and F). Correspondingly, CD8^+^ T cells in tumors with high glycolysis scores exhibited a trend toward negative regulation of immune response and leukocyte activation. This was characterized by upregulated expression of exhaustion markers (HAVCR2, CTLA4, TOX) and reduced expression of cytotoxicity and activation markers (GZMA, GZMK, TNF, LTB, CD69, TCF7, KLRB1) (Figure 1I and Supplemental Figure 1F). Together, these findings suggest that glycolysis and its associated lactylation contribute to an immunosuppressive TME, marked by enhanced accumulation of protumor macrophages. Given their high level of lactylation, we reasoned that macrophages might play an important role in inhibiting T cell infiltration and function.

### Elevated lactylation is associated with immunotherapy resistance in PDAC.

**Figure 2:**
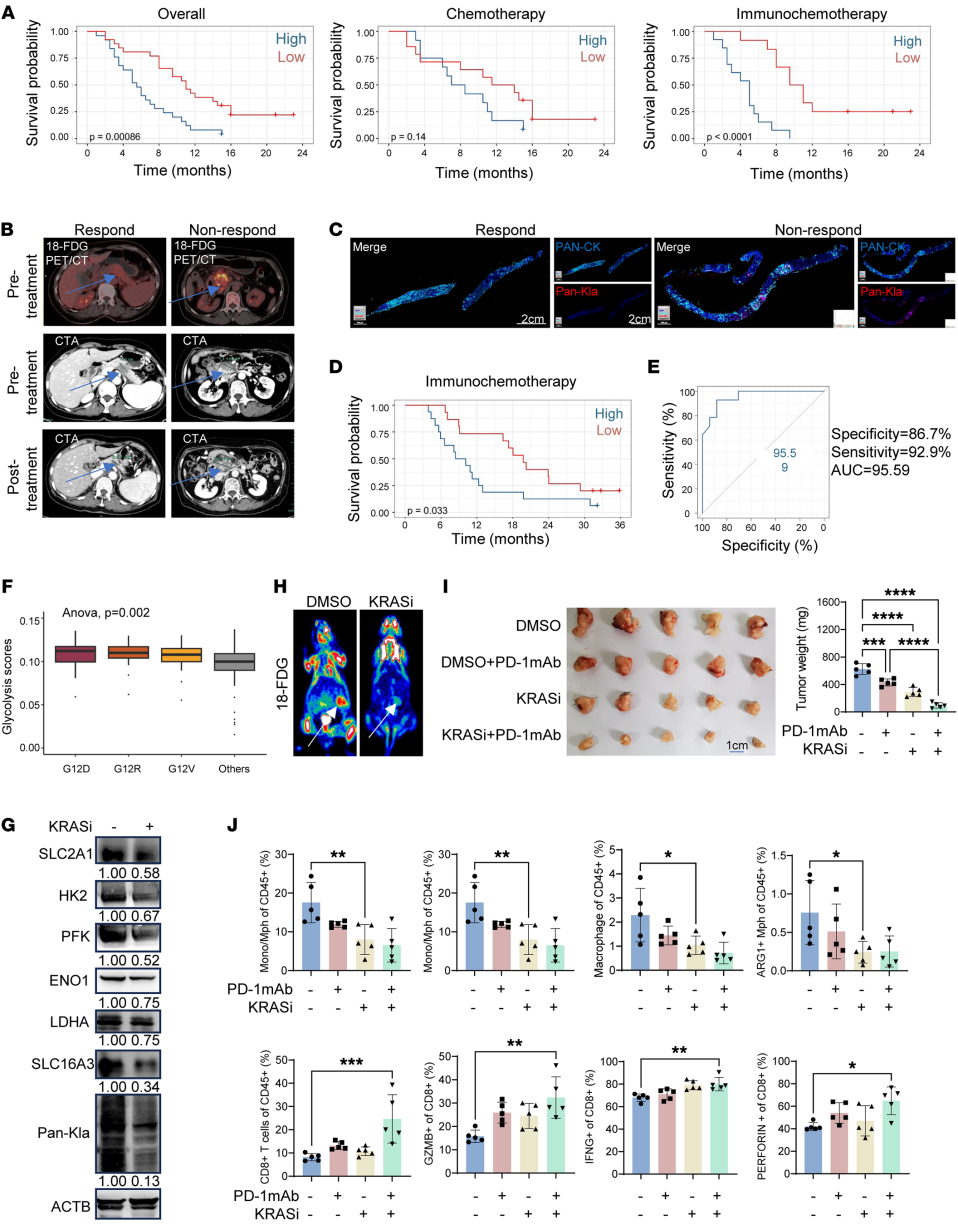
Elevated lactylation is associated with immunotherapy resistance in PDAC. (**A**) Kaplan-Meier survival curves of PFS for patients with PDAC based on the impact of ^18^F-FDG uptake with treatment. The median of ^18^F-FDG SUVmax was used to divide the cohort (cut off: 8.1). Statistical analyses were performed with log-rank test. (**B**) Representative images of ^18^F-FDG PET/CT and computed tomography angiography (CTA) of patients. Tumors are shown with a blue arrow. (**C**) Pan-Kla levels were analyzed by immunostaining of 31 biopsy specimens. Representative images of patients are shown. Scale bars: 2 cm. (**D**) Overall survival analysis was performed based on mean fluorescence intensity of Pan-Kla immunostaining. (**E**) ROC-AUC plot analysis was performed based on mean fluorescence intensity of Pan-Kla. Statistical analyses were performed with log-rank test. Prediction model of immunochemotherapy response situation was established. (**F**) Glycolysis score of patients with different KRAS mutation status using TCGA-PAAD database. Statistical analyses were performed with 1-way ANOVA. (**G**) Protein expression analysis of KPC cells treated with MRTX1133 (1 μM, 24 hours) or DMSO. (**H**) Representative images of ^18^FDG PET/CT of KPC orthotopic transplantation model treated with MRTX1133 (0.2 mg/mouse, i.p., qd) or DMSO. Tumors are shown with a white arrow. (**I**) Orthotopic transplantation tumors and statistical analysis are shown. KPC mice were individually treated by anti–PD-1 mAb (100 μg/mouse, i.p., tid) or MRTX1133 (0.2 mg/mouse, i.p., qd). Statistical analyses were performed with 1-way ANOVA (*n* = 5). (**J**) Proportions of each type of immunocytes and function markers in CD8^+^ T cells. The immune microenvironments of orthotopic transplantation model with different treatments were compared. Statistical analyses were performed with 1-way ANOVA (*n* = 5). *P < 0.05; **P < 0.01; ***P < 0.001.

Because of their association with immunosuppressive TME, we aimed to determine whether glycolysis and resultant lactylation contribute to immunotherapy resistance in PDAC. ^18^F-FDG PET/CT has been widely used in clinic to reveal glucose metabolism abnormalities in infection and inflammation, yet its use in diagnosing primary pancreatic cancer is uncommon (35). Our team previously performed a prospective cohort study that enrolled PDAC patients who received ^18^F-FDG PET/CT scans before chemotherapy or immunochemotherapy between August 2021 and February 2023 (35). We reviewed this cohort and assessed whether the standard uptake value (SUV) on PET scans correlated with immunotherapy outcomes (Supplemental Table 2). Among the 51 patients, 26 received chemotherapy alone and 25 received immunochemotherapy plus chemotherapy. Based on median SUVmax (cutoff = 8.1), the cohort was divided into high-uptake and low-uptake groups. The low-uptake group showed significantly improved PFS with immunochemotherapy compared with the high-uptake group, although this effect was not observed with chemotherapy alone (Figure 2A). The ORR was notably higher in the low-uptake group with immunochemotherapy (6/12) compared with the high-uptake group (1/13). These findings suggest that elevated glycolysis may influence the response to immunochemotherapy in PDAC patients. Representative pretreatment and posttreatment ^18^F-FDG PET/CT images of patients who did or did not respond to immunochemotherapy are illustrated in Figure 2B.

To investigate the correlation between lysine lactylation levels in tumor tissues and clinical outcomes of PDAC patients, biopsy specimens from 31 patients enrolled in a randomized phase II CISPD3 trial (ClinicalTrials.gov NCT03977272) were analyzed using immunohistochemical staining with Pan-cytokeratin (Pan-CK) and Pan-Kla antibodies (Figure 2C and Supplemental Figure 2, A and B, and Supplemental Table 3). This trial evaluated whether combining sintilimab (PD-1 monoclonal antibody) with modified FOLFIRINOX (mFFX) improves outcomes compared with mFFX alone for advanced pancreatic cancer (3). Elevated lactylation levels, measured by mean fluorescence intensity of Pan-Kla in tumor tissues, were significantly correlated with poorer patient survival (Figure 2D). Additionally, lactylation levels in tumor tissues demonstrated high specificity (86.7%), sensitivity (92.9%) and an AUC (95.59) for predicting outcome of immunotherapy, using overall survival time as the outcome metric (Figure 2E). These findings collectively suggest that increased glycolysis and consequent protein lactylation in tumors are associated with immunotherapy resistance in PDAC, underscoring the potential utility of lactylation levels as predictive biomarkers in PDAC immunotherapy outcomes.

We further investigated whether pancreatic cancer tissue features contribute to immunotherapy resistance. Approximately 90% of pancreatic cancers harbor KRAS mutations. It has been long known that KRAS G12 mutations promote glycolysis and anabolic metabolism in cancer cells (36). Indeed, our analysis of the pancreatic cancer database in TCGA-PAAD revealed that tumors with KRAS G12 mutations had significantly higher glycolysis scores than other tumors (Figure 2F). MRTX1133 is a newly synthesized selective KRAS-G12D inhibitor that entered clinical trials in 2023 (37, 38). Treatment of the Kras and Trp53 mutant pancreatic cancer (KPC) cell line — a genetically engineered mouse model (*KrasLSL-G12D/+*; *Trp53LSL-R172H/+*; *Pdx1-Cre*) — with MTRX133 downregulated the expression of key glycolysis-related genes, including SLC2A1, HK2, PFK, ENO1, LDHA, and SLC16A3 (Figure 2G and Supplemental Figure 2C). As a result, MRTX1133 significantly decreased the levels of global lactylation in the tumor cells (Figure 2G). In the KPC cell–derived orthotopic mouse PDAC model, MRTX1133 treatment significantly decreased glucose utilization of the tumors shown by ^18^F-FDG PET/CT (Figure 2H) and also inhibited tumor growth, but interestingly, the inhibition of KRAS dramatically sensitized the KPC tumors to anti–PD-1-based ICB (Figure 2I). Analysis of tumor immune profiles with flow cytometry showed that treatment with MRTX1133 decreased tumor infiltration of bone-marrow–derived monocytes (CD11b^+^Ly6C^hi^Ly6G^–^) and, particularly macrophages (CD11b^+^F4/80^+^IA/IE^+^) and protumoral macrophages (ARG1^+^ macrophages) (Figure 2J). By contrast, MRTX1133 treatment increased CD8^+^ T cell infiltration and activity (Figure 2J).

### Inhibiting glycolysis reduces the tumor cell–secreted CCL2 levels.

**Figure 3:**
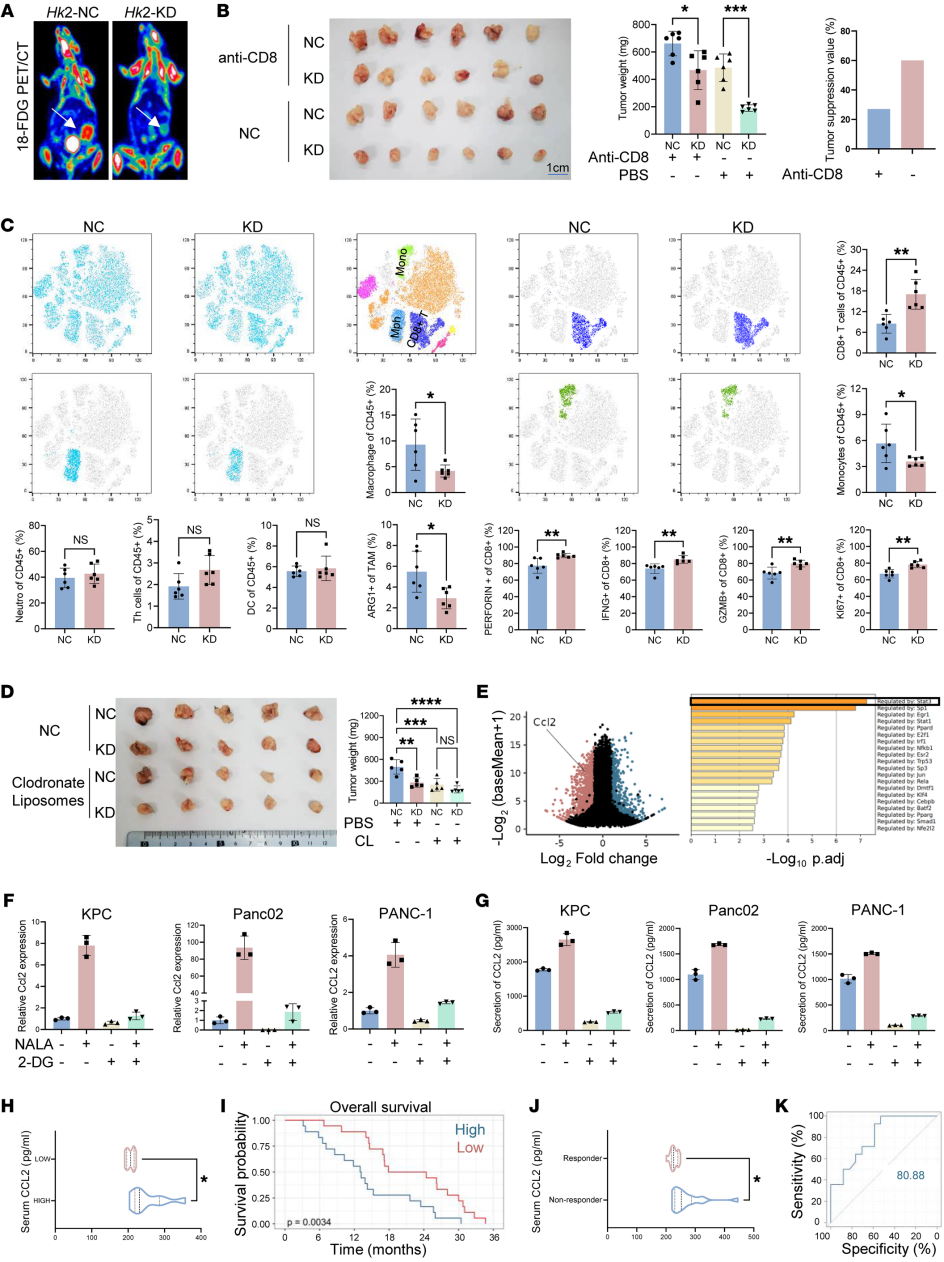
Inhibiting glycolysis reduces the levels of CCL2 secreted by tumor. (**A**) Representative images of ^18^FDG PET/CT of *Hk2*-NC and *Hk2*-KD KPC orthotopic transplantation models. Tumors are shown with a white arrow. (**B**) *Hk2*-NC and *Hk2*-KD KPC orthotopic transplantation mice were individually treated with or without anti-CD8 mAb (100 μg/mouse, i.p., tid). Statistical analyses were conducted with 1-way ANOVA (*n* = 6). (**C**) t-Distributed stochastic neighbor embedding (t-SNE) analysis and proportions of each type of immunocytes. Statistical analyses were performed with Student’s *t* test (*n* = 6). (**D**) Hk2-NC and Hk2-KD KPC orthotopic transplantation mice were individually treated with or without clodronate liposomes (1mg/mouse, i.p, q7d). Statistical analyses were performed with 1-way ANOVA (*n* = 5). (**E**) Volcano map of the RNA-Seq and transcriptional activity assay. KPC cells were treated with 2-DG (HK2 inhibitor, 10 mM, 24 hours), and differentially expressed genes are shown. Ccl2 was significantly downregulated when treated with 2-DG. Top 1,000 downregulated genes were validated by transcriptional activity assay using Metascape. (**F**) Relative *Ccl2* mRNA expression is shown. (**G**) Relative CCL2 secretion tested by ELISA is shown. (**H**) Concentration of serum CCL2. A total of 20 fresh PDAC samples were divided into 2 groups according to Pan-Kla expression. Statistical analyses were performed with Student’s *t* test (*n* = 20). (**I**) Overall survival analysis was performed based on concentration of serum CCL2. Patients were divided into 2 groups according to the medium amount of serum CCL2. Statistical analyses were performed with log-rank test (*n* = 36). (**J**) Concentration of serum CCL2 tested by ELISA. Thirty-one PDAC samples involved in the CISPD3 RCT study were divided into 2 groups according to their response to immunochemotherapy. (**K**) ROC-AUC plot analysis was performed based on the concentration of CCL2. Prediction model of immunochemotherapy response situation was established. *P < 0.05; **P < 0.01; ***P < 0.001; ****P < 0.0001.

Given the essential role of HK2 in glycolysis and resultant protein lactylation, we generated a KPC cell line with knockdown (KD) of *Hk2* and used it for an orthotopic mouse tumor model (39) (Figure 1C and Supplemental Figure 3A). KD of *Hk2* significantly decreased ^18^F-FDG uptake and tumor weight in vivo (Figure 3, A and B). Depletion of CD8^+^ T cells from the mice partially offset the tumor-inhibiting effect (60% vs 27%) by Hk2 KD in comparison with the control mice (Figure 3B and Supplemental Figure 3B). Glycolysis inhibition by *HK*2 KD had similar effects on the tumor immune microenvironment as observed in KRAS inhibition by MRTX1133, including decreased macrophage infiltration and increased CD8^+^ T cell infiltration and activity (Figure 3C).

Considering that elevated glycolysis increases macrophage infiltration and enhances protumor functions, we reasoned that aberrant glycolysis in tumor cells may suppress T cell activity through macrophages. By using clodronate liposomes to eliminate macrophages from the mice bearing KPC tumors, we found that macrophage deletion significantly decreased tumor weight (Figure 3D and Supplemental Figure 3C). However, *Hk2* KD did not exert additional tumor suppression activity, suggesting that the immunological effect of glycolysis on tumor growth was mediated by tumor-infiltrated macrophages (Figure 3D). To examine how elevated glycolysis in tumors contributes to macrophage infiltration, we used bulk RNA-Seq to identify glycolysis-dependent transcriptomics in KPC cells treated with or without 2-deoxy-d-glucose (2-DG), a specific HK2 inhibitor. Among the most significantly altered genes (log fold change [log FC] < –1.5, *P* adjusted < 0.05, base mean > 500), CCL2 is the only recognized chemokine for recruiting macrophages and monocytes (40) (Figure 3E). We found that KD of HK2 decreased CCL2 expression and secretion in KPC cells (Supplemental Figure 3D). We also confirmed that treatment with 2-DG significantly reduced CCL2 expression in human PDAC cell lines, Panc02 and PANC-1, while supplementation of sodium lactate (NALA), the end product of the glycolysis, increased CCL2 expression (Figure 3F). In line with the CCL2 expression levels, the secretion of CCL2 by tumor cells decreased when treated with 2-DG and increased when treated with NALA (Figure 3G). As a control, *Csf1*, another important monocyte/macrophage chemokine, was not significantly changed when treated with 2-DG or NALA (14) (Supplemental Figure 3E). We checked serum CCL2 levels in the previous cohort of 20 patients with high or low Pan-Kla levels. We found that the higher Pan-Kla group had higher serum CCL2 levels (Figure 3H and Supplemental Table 1). In a cohort of PDAC patients with available serum samples and clinical outcome information, we found that high serum CCL2 levels were significantly correlated with poor overall survival (Figure 3I and Supplemental Table 4).

To determine whether CCL2 acts as a key factor in immunotherapy resistance, we analyzed the previously mentioned randomized clinical trial cohort for PDAC immunotherapy (3). We found that the serum CCL2 levels in nonresponders were significantly higher than those in responders (Figure 3J and Supplemental Table 3). Serum CCL2 levels are closely related to immunotherapy outcomes, with an AUC value of 80.88 (specificity: 54.94; sensitivity:100 with cutoff 250.9756), using overall survival time as the outcome metric (Figure 3K and Supplemental Table 3). The results suggested that PDAC patients with high serum CCL2 are more likely to develop immunotherapy resistance. In the orthotopic KPC mouse tumor model, ICB using anti–PD-1 mAbs had minimal effect on tumor suppression. However, treatment with PF-4136309, a potent and selective CCR2 antagonist, drastically sensitized tumors to the anti–PD-1 therapy (Supplemental Figure 4A). In line with this result, anti–PD-1 immunotherapy was more effective for the KPC tumors with *Hk2* KD, while PF-4136309 had no effect on the HK2-KD KPC tumors compared with the vehicle control treatment (Supplemental Figure 4A). Analysis of tumor-infiltrated lymphocytes revealed that KD of *Hk*2 or inhibiting CCL2 signaling in KPC tumors reduced monocyte/macrophage infiltration but enhanced T cell infiltration and function (Supplemental Figure 4B). Collectively, these results demonstrated that inhibition of glycolysis sensitized PDAC to immunotherapy, with CCL2 acting as a key factor.

### ENSA K63 lactylation upregulates STAT3/CCL2 signaling in tumor cells.

**Figure 4:**
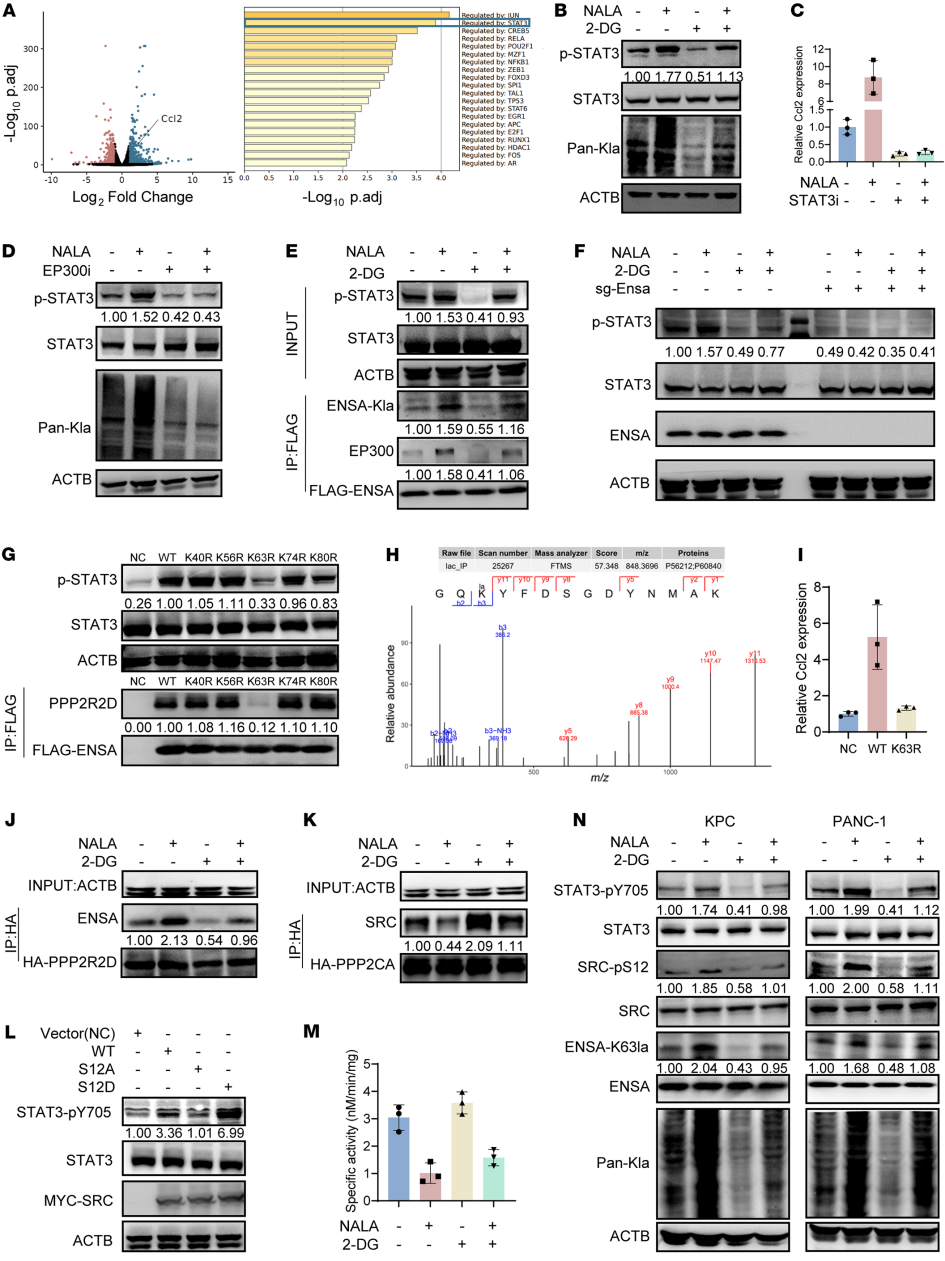
ENSA K63 lactylation upregulates STAT3/CCL2 signaling via PP2A and SRC. (**A**) Volcano map of the RNA-Seq analysis. Top 1,000 upregulated genes were deconvoluted to reveal related transcriptional factors using Metascape. (**B**) Protein expression analysis of KPC cells treated with 2-DG (10 mM, 24 hours) or NALA (40 mM, 24 hours). (**C**) Relative *Ccl2* mRNA expression is shown with 3 technical replicates. KPC cells were treated with STAT3-IN-11 (1 μM, 24 hours) or NALA (40 mM, 24 hours). (**D**) Protein expression analysis of KPC cells treated with A-485 (EP300 inhibitor, 1 μM, 24 hours) or NALA (40 mM, 24 hours). (**E**) Protein expression and immunoprecipitation-immunoblotting analyses of KPC cells treated with 2-DG (10 mM, 24 hours) or NALA (40 mM, 24 hours). Anti-FLAG antibody was used to immunoprecipitate ENSA-FLAG proteins. (**F**) Protein expression analysis of *Ensa*-NC and *Ensa*-KO KPC cells treated with 2-DG (10 mM, 24 hours) or NALA (40 mM, 24 hours). (**G**) Protein expression analysis of each *Ensa*-KO KPC cell line overexpressing ENSA-NC, ENSA-WT-FLAG, ENSA-K40R-FLAG, ENSA-K56R-FLAG, ENSA-K63R-FLAG, ENSA-K74R-FLAG, or ENSA-K80R-FLAG. (**H**) Mass spectrum analysis revealed that ENSA is lactylated at K63 site. (**I**) Relative *Ccl2* mRNA expression of each *Ensa*-KO KPC cell line overexpressing vector control, ENSA-WT, or ENSA-K63R is shown. (**J**) Protein expression and immunoprecipitation-immunoblotting analyses of KPC-HA-PPP2R2D cell line treated with 2-DG (10 mM, 24 hours) or NALA (40 mM, 24 hours). Anti-HA antibody was used to immunoprecipitate HA-PPP2R2D proteins. (**K**) Protein expression and immunoprecipitation-immunoblotting analysis of KPC-HA-PPP2CA cell line treated with 2-DG (10 mM, 24 hours) or NALA (40 mM, 24 hours). Anti-HA antibody was used to immunoprecipitate HA-PPP2CA proteins. (**L**) Protein expression analysis of each KPC cell line overexpressing vector (control), SRC-WT, SRC-S12A, or SRC-S12D. (**M**) SRC-pS12-specific activity of PP2A phosphatase. SRC-pS12 cell-penetrating peptide (HLYVSPWGG-SKPKDApSQRRRSL) was incubated with KPC cells treated with 2-DG (10 mM, 24 hours) or NALA (40 mM, 24 hours). (**N**) Protein expression analysis of KPC and PANC-1 cells treated with 2-DG (10 mM, 24 hours) or NALA (40 mM, 24 hours).

In both bulk RNA-Seq analyses of KPC cells treated with 2-DG or NALA, *Ccl2* was found significantly downregulated or induced (Figure 3E and Figure 4A). Additionally, STAT3 is a transcriptional factor associated with lactate metabolism among most differentially expressed genes (16, 41) (Figure 4A). We also confirmed that NALA upregulated the activity of STAT3, indicated by its tyrosine 705 (Y705) phosphorylation level, while 2-DG downregulated STAT3 phosphorylation (Figure 4B and Supplemental Figure 5A). The results were consistent with previous studies showing that CCL2 was transcriptionally regulated by STAT3 (42–44). Treatment of KPC cells with STAT3 inhibitor (STAT3-IN-11) decreased the mRNA levels of *Ccl2*, and this effect was not restored by NALA treatment (Figure 4C). Previous studies have shown that cell-intrinsic glycolytic activity and extracellular uptake of lactate can regulate intracellular lactate levels accompanied by protein lactylation levels (20, 23, 39). Considering that NALA did not significantly change the pH of the culture medium, lactate may act as a signaling molecule to regulate STAT3 phosphorylation. Lactylation is a dynamic process in a cell, and EP300 has been identified as a primary writer for both histone and nonhistone lactylation (24). With treatment of KPC cells with a EP300 inhibitor (A-485), STAT3 phosphorylation was decreased, and this effect could not be restored by the addition of NALA (Figure 4D and Supplemental Figure 5B). To identify lactylated proteins that regulate STAT3 phosphorylation, we conducted proteomics analysis to search for lactylated proteins on both human pancreatic tumor samples (*n* = 3) and mouse KPC cells. Out of 324 lactylated proteins identified in both human and mouse samples, 3 proteins — endosulfine α (ENSA), CDC28 protein kinase regulatory subunit 1B (CKS1B), and nuclear casein kinase and cyclin dependent kinase substrate 1 (NUCKS1) — are potential regulators for protein phosphorylation based on gene ontology analysis (Supplemental Table 5). Further validation showed that ENSA overexpression increased the phosphorylation of STAT3, but CKS1B or NUCKS overexpression did not (Supplemental Figure 5C). ENSA is a highly conserved cAMP-regulated phosphoprotein. We indeed confirmed lactylation of ENSA in KPC cells (Figure 4E). Previous studies revealed that inhibiting cellular glycolysis with 2-DG and mimicking extracellular lactate uptake with NALA can regulate intracellular lactate levels, which could be used as a tool to regulate the overall level of protein lactylation (20, 23, 39). Treatment of KPC cells with 2-DG decreased ENSA lactylation and interaction with EP300, while treatment with NALA produced the opposite effects, supporting that EP300 was the writer of ENSA lactylation (Figure 4E and Supplemental Figure 5D). To confirm whether NALA or 2-DG regulated phosphorylation of STAT3 in an ENSA-dependent manner, we constructed an *Ensa*-KO KPC cell line and found that knockout of *Ensa* decreased phosphorylation of STAT3, and this effect could not be restored by NALA (Figure 4F and Supplemental Figure 5E).

Proteomics analysis with mass spectrometry identified multiple lactylation sites on ENSA, including K40, K56, K63, K74, and K80. To determine the key lactylation event for STAT3 signaling, we mutated each lactylation site from lysine to arginine (K to R) on ENSA. While overexpression of ENSA-WT, ENSA-K40R, ENSA-K56R, ENSA-K74R, and ENSA-K80R in *Ensa*-KO KPC cells upregulated phosphorylation of STAT3, ENSA-K63R overexpression did not (Figure 4, G and H, and Supplemental Figure 5F). To better quantify the differences, we conducted an ELISA experiment to measure phosphorylated STAT3 (p-STAT3) levels. We found that the p-STAT3 levels in cells overexpressing ENSA-K63R were comparable to those of the control group, while overexpression of ENSA-WT and other mutants led to a 3-fold increase in p-STAT3 levels (Supplemental Figure 5G).

Quantitative PCR revealed that *Ccl2* expression increased with ENSA-WT overexpression but not with ENSA-K63R overexpression (Figure 4I). These results demonstrated that ENSA-K63 lactylation is a key event for STAT3/CCL2 signaling in pancreatic cancer cells.

ENSA is a protein phosphatase inhibitor that specifically inhibits protein phosphatase 2A (PP2A). In the cell, PP2A is a heterotrimeric complex that consists of a structural subunit A, a catalytic subunit C, and a regulatory subunit B. ENSA is known to physically interact with a regulatory subunit of PP2A, PP2A regulatory subunit B δ isoform (PPP2R2D), and thus inhibit the catalytic activity of PP2A (45). The interaction between ENSA and PPP2R2D was enhanced in KPC cells treated with NALA, whereas the interaction was weakened in the cells treated with 2-DG (Figure 4J and Supplemental Figure 5H). The results suggest that ENSA lactylation inhibits PP2A activity by enhancing the ENSA-PPP2R2D interaction. We also identified proto-oncogene, non-receptor tyrosine kinase (SRC), an upstream tyrosine kinase for STAT3, as an interactor of the PP2A catalytic subunit PPP2CA (PP2A catalytic subunit α) by mass spectrometry (Supplemental Table 6). The PPP2CA-SRC interaction was enhanced in KPC cells treated with 2-DG, whereas the interaction was weakened with cells treated with NALA (Figure 4K and Supplemental Figure 5I) (46). This is consistent with previous reports that PP2A dephosphorylates SRC at S12 and decreases the kinase activity of SRC (47–50). To validate the kinase activity of SRC on STAT3, constitutive phosphorylation (S12D) and nonphosphorylation (S12A) mutants were constructed to further investigate the function of S12 phosphorylation. The S12D mutation of SRC upregulated STAT3-Y705 phosphorylation, while the S12A mutation of SRC downregulated STAT3-Y705 phosphorylation (Figure 4L and Supplemental Figure 5J), highlighting the importance of SRC-S12 phosphorylation on its kinase activity. In a phosphatase assay, we tested the SRCpS12-specific phosphatase activity of PP2A in the KPC cells. The dephosphorylation activity of PP2A was decreased when the cells were treated with 2-DG, but increased upon NALA treatment (Figure 4M).

To demonstrate the endosulfine α (ENSA-K63la)/SRC-pS12/STAT3-pY705 signaling cascade, we generated antibodies that specifically identify ENSA-K63la or SRC-pS12. Western blotting analysis revealed that treatment with NALA upregulated ENSA-K63 lactylation, SRC-S12 phosphorylation, and STAT3-Y705 phosphorylation, while 2-DG downregulated ENSA-K63 lactylation, SRC-S12 phosphorylation, and STAT3-Y705 phosphorylation (Figure 4N and Supplemental Figure 5K) in both human and mouse PDAC cell lines. We also conducted relevant experiments using an LDHA inhibitor, sodium oxamate (20). After treating KPC and PANC-1 cell lines with the previously reported dose of 20 μM for 24 hours, ENSA-K63la, SRC-pS12, and STAT3-pY705 levels were significantly decreased while this effect was largely weakened by the addition of NALA (Supplemental Figure 5, L–N). Taken together, these data show that heightened glycolysis in pancreatic cancer cells causes lactate accumulation, leading to upregulated protein lactylation. Particularly, ENSA-K63 lactylation inactivates PP2A, enhances SRC kinase activity, and eventually increases STAT3-Y705 phosphorylation and boosts CCL2 secretion.

### Design of a peptide inhibitor specifically targeting ENSA-K63la.

**Figure 5:**
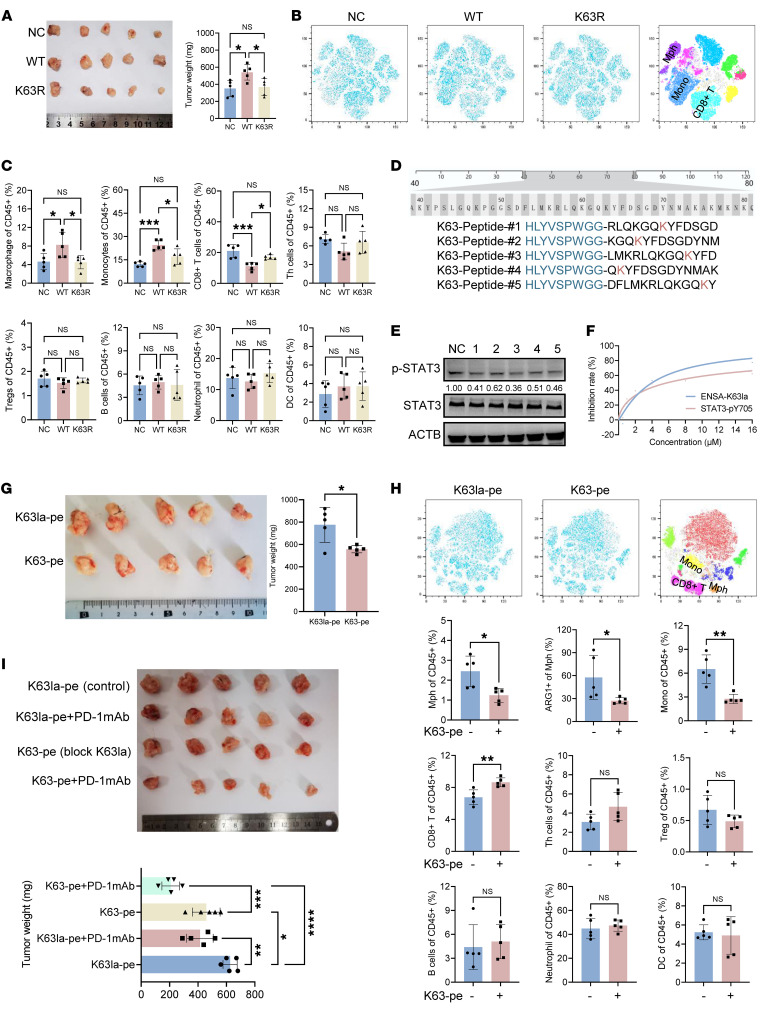
Design of a peptide inhibitor specifically targeting ENSA-K63la. (**A**) *Ensa*-KO KPC cells stably expressing vector control, ENSA-WT, and ENSA-K63R were orthotopically transplanted into mice and tumor growth was analyzed. Statistical analyses were performed with 1-way ANOVA (*n* = 5). (**B** and **C**) t-SNE analysis (**B**) and proportions (**C**) of each type of immunocytes in the tumors in **A**. The immune profiling was analyzed using Cytek. Statistical analyses were performed with 1-way ANOVA (*n* = 5). (**D**) Human and mouse ENSA protein sequences are shown. Cell-penetrating peptide targeting ENSA-K63la was designed around K63 site. (**E**) Protein expression analysis of KPC cells treated with individual K63-peptide (10μM, 24h). (**F**) Relative inhibition rate of STAT3-Y705 phosphorylation. KPC cells treated with K63-peptide inhibitor 3 (HLYVSPWGG-LMKRLQKGQKYFD) and K63la-peptide control 3 (HLYVSPWGG-LMKRLQKGQKlaYFD) with different concentrations (0 μM; 0.5 μM; 1 μM; 2 μM; 4 μM; 8 μM; 16 μM) for 24 hours. Immunoblotting band intensity was measured using Image J. The relative levels of ENSA-K63la and STAT3-pY705 were normalized by the levels of ACTB. The relative inhibition rate at a certain concentration was calculated by 1– expression (K63 inhibitor 3)/expression (K63la control 3). IC_50_ was calculated by nonlinear regression. (**G**) Orthotopic transplantation tumors and statistical analysis are shown. KPC orthotopic transplantation mice were treated with K63la-pe control 3 (control) or K63-pe inhibitor 3 (0.2 mg/mouse, i.p., qd). Tumor growth was analyzed with Student’s *t* test (*n* = 5). (**H**) t-SNE analysis and proportions of each type of immunocytes in the tumors in **G**. The immune profile of tumors was analyzed using Cytek. Statistical analyses were performed with Student’s *t* test (*n* = 5). (**I**) Orthotopic transplantation tumors and statistical analysis are shown. KPC mice were treated with anti-PD-1 mAb (100 μg/mouse, i.p., tid), K63la-pe control 3, or K63-pe inhibitor 3 (0.2 mg/mouse, i.p., qd). Tumor growth was analyzed with 1-way ANOVA (*n* = 5). *P < 0.05; **P < 0.01; ***P < 0.001; ****P < 0.0001.

We tested to determine whether ENSA-K63 lactylation makes a major contribution to an immunosuppressive TME in PDAC. To this end, we examined the orthotopic tumor growth and progression using mouse *Ensa*-KO KPC cells overexpressing WT (ENSA-WT) or K63 mutant ENSA (ENSA-K63R). Overexpression of ENSA-WT significantly increased tumor growth, while overexpression of ENSA-K63R did not (Figure 5A). Flow cytometric analysis revealed that overexpression of ENSA-WT markedly increased macrophage/monocyte infiltration but decreased CD8^+^ T cell infiltration and function, similar to the effect of elevated glycolysis on the immunosuppressive microenvironment (Figure 5, B and C, and Supplemental Figure 6A). Given its important role in pancreatic tumor progression, ENSA-K63la is thought of as a potential therapeutic target. Previous studies have demonstrated that cell-penetrating peptides are a promising and effective approach to inhibiting posttranslational modification of target proteins (23). Because the amino acids in the vicinity of K63 are conserved in humans and mice, we designed 5 individual peptides as competitive inhibitors for inhibiting both human and mouse ENSA-K63la (Figure 5D). A cell-penetrating peptide (HLYVSPWGG) was added to N-terminus of each peptide inhibitor (23). Y705 phosphorylation of STAT3 was used as an indicator for the inhibitory activity of the peptide inhibitors. After incubation with each peptide inhibitor (K63-pe) for 24 hours, KPC cells were tested for decreased Y705 phosphorylation of STAT3 (Figure 5E). Among the 5 peptide inhibitors, K63-pe inhibitor 3 exhibited the best inhibitory activity of STAT3 (IC_50_ = 5.797 μM) in comparison with vehicle control and negative control K63la-pe-3 (Figure 5, E and F). In the orthotopic KPC tumor model, K63-pe-3 also showed great antitumor activity when used at the dose of 10 mg/kg every day with i.p. injection (Figure 5G). Analysis of tumor-infiltrating lymphocytes also demonstrated that monocytes, macrophages, and ARG1^+^ macrophages were decreased and CD8^+^ T cells were increased with treatment of K63-pe (Figure 5H and Supplemental Figure 6B). In vivo experiments also revealed that K63-pe-3 sensitized anti-PD-1 immunotherapy in the orthotopic KPC mouse model (Figure 5I).

### Lactate accumulation reprograms TAMs by ENSA lactylation.

**Figure 6:**
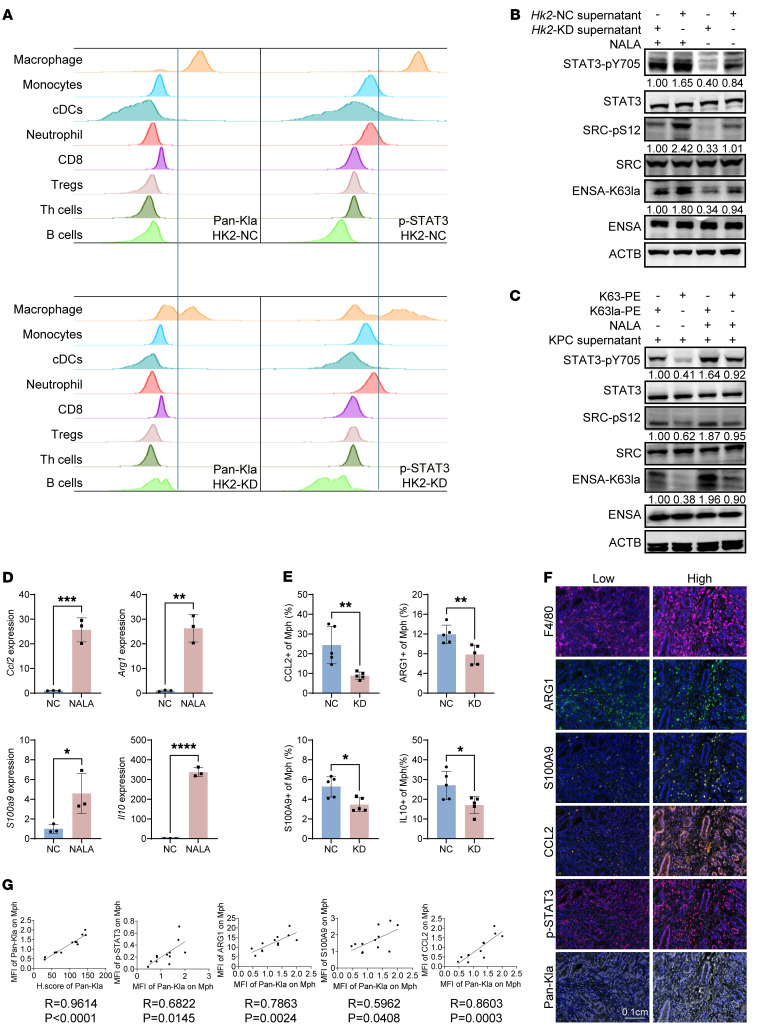
Lactate accumulation reprograms TAMs by ENSA lactylation. (**A**) Pan-Kla and p-STAT3 expression were tested using intracellular flow cytometry in each cell type from *Hk2*-NC and *Hk2*-KD KPC orthotopic tumors. (**B**) Protein expression analysis of BMDMs pretreated with *Hk2*-NC and *Hk2*-KD KPC cell supernatant and then treated with NALA (10 mM, 24 hours). (**C**) Protein expression analysis of BMDMs pretreated with KPC cell supernatant (1:1, 24 hours) and then treated with K63la-pe control 3 or K63-pe inhibitor 3 (10 μM, 24 hours). (**D**) BMDMs were pretreated with KPC supernatant (1:1, 24 hours) and then treated with NALA (40 mM, 24 hours). Relative *Ccl2*, *Arg1*, *S100A9*, and *Il10* mRNA expression levels are shown. (**E**) CCL2, IL10, ARG1, and S100A9 expression in TAMs. Flow cytometry was used. Statistical analyses were performed with Student’s *t* test (*n* = 5). (**F**) Representative images of mIHC staining of KTC tumor samples from transgenic mice (LSL-Kras [G12D/+]; Tgfbr2 [flox/flox]; p48 [Cre/+]). Representative images of paraffin sections with high or low Pan-Kla expression are shown. (**G**) Simple linear regression was used to reveal correlation of p-STAT3, CCL2, ARG1, S100A9, and Pan-Kla (*n* = 12). *P < 0.05; **P < 0.01; ***P < 0.001; ****P < 0.0001.

Lactate, mainly produced by tumor cells through glycolysis, is secreted into the TME as a signaling molecule (51, 52). We have shown that elevated glycolysis not only increased macrophage infiltration, but also promoted a protumor signature (Figure 1, G and H, and Supplemental Figure 1, E and F). In the microenvironment of KPC-derived mouse tumors, TAMs showed the highest levels of Pan-Kla and p-STAT3 among all the immunocytes (Figure 6A). *Hk2* KD in the tumor cells significantly decreased Pan-Kla and p-STAT3 levels in TAMs but not in other immunocytes, indicating that TAMs are the primary immune cells affected by tumor-derived lactate (Figure 6A). We verified in vitro whether CD8^+^ T cells, another important immune cell type in this study, are unable to upregulate their own lactylation modifications when stimulated by exogenous lactate. We also isolated naive CD8^+^ T cells (CD45^+^CD3^+^CD8^+^CD25^-^) from the spleen. We found that when CD8^+^ T cells were activated using anti-CD3/anti-CD8/IL-2 stimulation (CD45^+^CD3^+^CD8^+^CD25^+^), the overall Pan-Kla levels increased compared with naive CD8^+^ T cells. However, these activated T cells (CD45^+^CD3^+^CD8^+^CD25^+^) were not affected by tumor-secreted lactate or additional NALA supplementation, with no significant changes in Pan-Kla levels. In contrast, for naive CD8^+^ T cells (CD45^+^CD3^+^CD8^+^CD25^–^), tumor-secreted lactate increased the intracellular Pan-Kla levels. Moreover, naive CD8^+^ T cells treated with the supernatant of HK2-NC KPC cells showed a more pronounced increase in intracellular Pan-Kla levels compared with those treated with the supernatant of HK2-KD KPC cells. Additionally, NALA supplementation also enhanced the intracellular Pan-Kla levels (Supplemental Figure 7A). These results were quite interesting. Previous studies reveal that in activated CD8^+^ T cells with high glycolysis-derived intracellular lactate, extracellular lactate does not impact protein lactylation. However, in naive CD8^+^ T cells with low glycolysis, extracellular lactate upregulates protein lactylation (53). Previous studies have revealed that TAMs demonstrate significant metabolic adaptability, enabling them to thrive in the TME (16, 54–56). TAMs often display a shift from glycolysis (characteristic of M1 macrophages) to oxidative phosphorylation and fatty acid oxidation (similar to M2 macrophages) (16, 54–56). This lactate derived from tumor cells not only fuels their metabolic needs, but also promotes an immunosuppressive phenotype. Overall, TAMs’ metabolic plasticity enables them to respond to the dynamic conditions of the TME, fostering their protumorigenic roles and contributing to immune evasion.

Using conditioned medium from control (*Hk2*-NC) and *Hk2*-KD KPC cell cultures to treat bone marrow–derived macrophages (BMDMs), we observed decreased levels of Pan-Kla, ENSA-K63 lactylation, SRC-S12 phosphorylation, and STAT3-Y705 phosphorylation in BMDMs when cultured with *Hk2*-KD KPC medium, compared with those BMDMs cultured with *Hk2*-NC KPC medium, and these reduced levels could be largely restored in the BMDMs cultured with NALA-treated *Hk2*-KD KPC medium (Figure 6B and Supplemental Figure 7B). We also found that treatment with K63-pe-3 inhibitor decreased ENSA-K63 lactylation, SRC-S12 phosphorylation, and STAT3-Y705 phosphorylation in BMDMs pretreated with KPC conditioned medium (Figure 6C and Supplemental Figure 7C). Collectively, these results suggest that tumor cell–derived lactate regulated the ENSA-K63la/SRC-pS12/STAT3-pY705 axis in TAMs.

A number of protumor genes downstream of STAT3 signaling, including *Ccl2*, *Arg1*, *S100A9*, and *IL10*, were significantly upregulated in TAMs upon NALA treatment (57–60) (Figure 6D). Knocking down HK2 in KPC significantly reduced CCL2, ARG1, S100A9, and IL10 expression in macrophages in vivo (Figure 6E). Compared with BMDMs cultured with *Hk2*-KD KPC medium, BMDMs cultured with *Hk2*-NC KPC medium also revealed higher expression levels of *Ccl2*, *Arg1*, *S100A9*, and *IL10*, which could be reduced when *Hk2*-NC KPC cells were treated with K63-pe-3 (Supplemental Figure 7D). We also found that BMDMs pretreated with *Hk2*-NC KPC medium had greater inhibitory activity on CD8^+^ T cell proliferation compared with BMDMs cultured with *Hk2*-KD KPC medium, and this effect could be restored when *Hk2*-NC KPC cells were pretreated with K63-pe-3 (Supplemental Figure 7E). Multiplex IHC staining of paraffin sections of pancreatic tumor tissues from patients and genetically modified mouse model (*Ptf1a-Cre*; *KrasLSL-G12D/+*; *Tgfbr2lox/lox*) showed that tumor tissues with overall higher scores of Pan-Kla had higher Pan-Kla levels in macrophages, suggesting that lactate accumulated in the microenvironment impacts Pan-Kla levels in TAMs (Figure 6, F and G, and Supplemental Figure 7, F and G).” As expected, Pan-Kla levels in TAMs were positively correlated with the levels of CCL2, ARG1, S100A9, and p-STAT3 (Figure 6, F and G, and Supplemental Figure 7, F and G).

Overall, the tumor cells’ autonomously enhanced glycolysis and protein lactylation modification upregulate CCL2 expression through the ENSA-K63la/SRC-pS12/STAT3-pY705 axis, thereby recruiting macrophages. The lactate secreted by the tumor further affects the infiltrating macrophages and promotes the protumoral signature. Collectively, tumor cell intrinsic lactate production and paracrine lactate lead to the enrichment of immunosuppressive macrophages in the microenvironment.

### ENSA-K63la/STAT3-pY705/CCL2 axis is a therapeutic target in PDAC.

**Figure 7:**
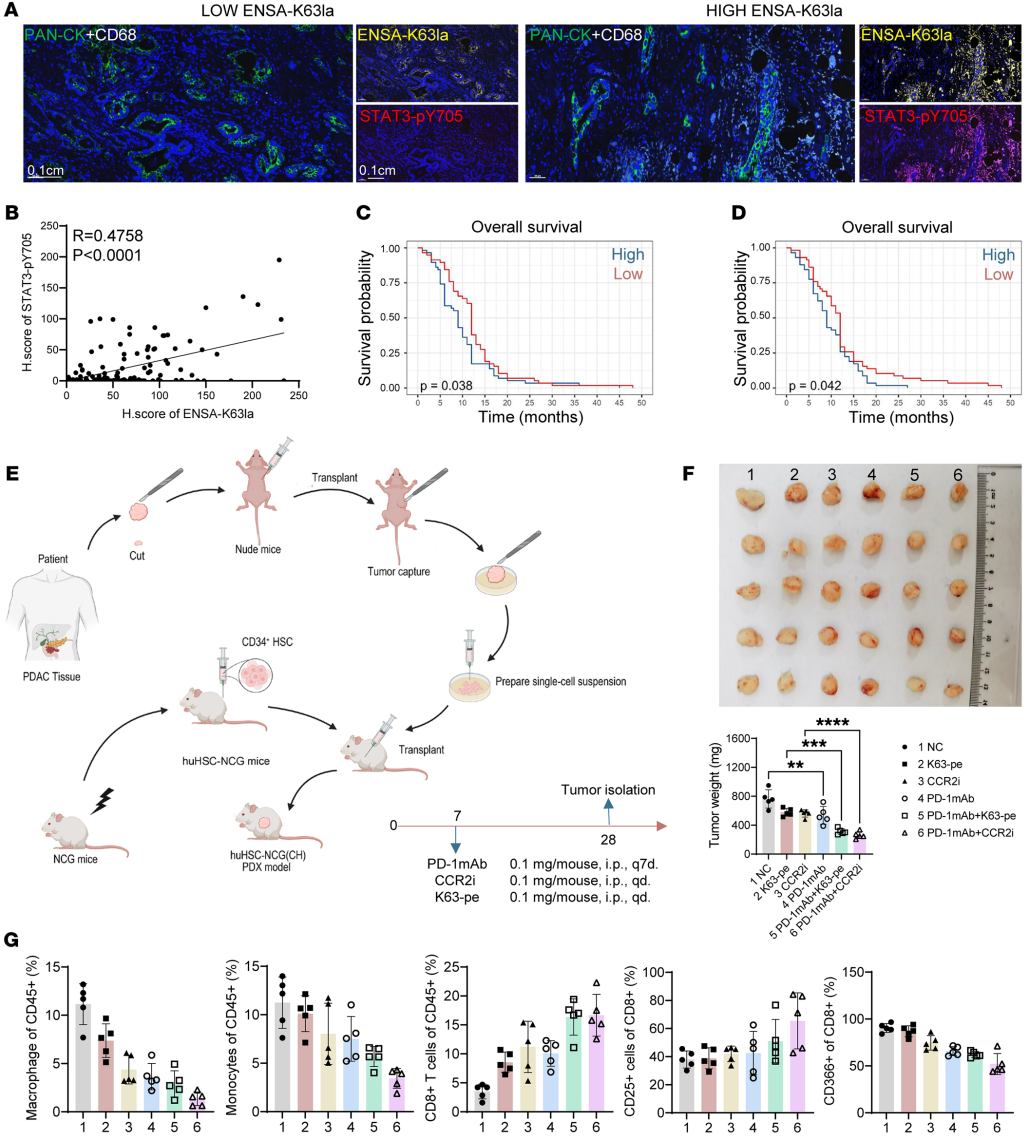
ENSA-K63la/STAT3-pY705/CCL2 axis is a therapeutic target for human PDAC. (**A**) Representative images of mIHC of human PDAC tumor tissues. (**B**) Simple linear regression was used to reveal correlation of ENSA-K63la and STAT3-pY705. Intact tissues with survival data were included in follow-up analysis (*n* = 116). (**C**) Overall survival analysis was performed based on maximum fluorescence intensity of ENSA-K63la. Statistical analyses were performed with log-rank test (*n* = 116). (**D**) Overall survival analysis was performed based on mean fluorescence intensity of STAT3-pY705. Statistical analyses were performed with log-rank test (*n* = 116). (**E**) Schematic diagram for generating humanized PDAC models. (**F**) Subcutaneous transplantation tumors and statistical analysis are shown. Statistical analyses were performed with 1-way ANOVA (*n* = 5). (**G**) Proportions of each cell type of immunocytes and each function marker in CD8^+^ T cells.

We further investigated the clinicopathological correlation of ENSA-K63la in PDAC using a tissue microarray of specific antibodies in mIHC assays. The results demonstrated that ENSA-K63la levels were positively correlated with STAT3-pY705 levels in human PDAC (Figure 7, A and B). Furthermore, high levels of ENSA-K63la and STAT3-pY705 were associated with poor overall survival of PDAC patients (Figure 7, C and D).

To assess the potential translational value of K63-peptide inhibitor and CCR2 inhibitor, a human CD34^+^ hematopoietic stem cell–transplanted (HSC-transplanted) NCG mouse model with patient-derived xenografts (PDX) was used (Figure 7E). Treatment with CCR2i or K63-pe-3 significantly decreased tumor growth and enhanced the efficacy of anti-PD-1 ICB therapy in the humanized PDX models (Figure 7F). Tumor immune profiling analysis revealed that treatment with CCR2i and K63-pe decreased macrophage and monocyte infiltration and increased CD8^+^ T cell infiltration (Figure 7G). In addition, activated CD8^+^ T cells (CD25^+^) were increased and exhausted CD8^+^ T cells (CD366^+^) were decreased with both the treatments (Figure 7G). Taken together, CCR2i or K63-pe is a promising therapeutic approach used in combination therapy with ICB in treating PDAC.

Since ENSA-K63-pe, CCR2i, and MRTX1133 can reshape the immune microenvironment of pancreatic cancer and enhance T cell infiltration and function, we tested whether these drugs have the ability to broadly sensitize tumors to immunotherapy (Figure 2, Supplemental Figure 4, Figure 5, Supplemental Figure 6, and Figure 7). We found that these drugs could sensitize tumors to anti–CTLA-4 immunotherapy as well, not just to anti-PD-1 therapy (Supplemental Figure 8A).

In conclusion, elevated glycolysis levels in PDAC promote ENSA-K63la, a crucial step that triggers STAT3/CCL2 signaling in tumor cells. Elevated CCL2 secretion by tumor cells facilitates tumor-associated TAM recruitment to the TME. Tumor-derived lactate drives transcriptional reprogramming (CCL2, ARG1, IL10, S100A9) in TAMs via the ENSA-K63la/STAT3-pY705 axis, promoting an immunosuppressive environment and immunotherapy resistance. Targeting mutant-KRAS, CCL2, or ENSA-K63la enhances the efficacy of ICB therapy in pancreatic tumor models. In addition, ^18^F-FDG uptake, pathological biopsy samples, Pan-Kla expression, and serum CCL2 levels in patients could predict clinical outcomes in immunotherapy.

## Discussion

The ORR of immunotherapy in PDAC is relatively low compared with that of other tumors (1). The unique genomic landscape and environmental features of pancreatic cancer result in different responses to immunotherapy. Identifying patients who are sensitive to anti-PD immunotherapy and developing combination therapies for those who may not respond is an urgent problem to be solved.

Elevated glycolysis alters the TME. In this study, we demonstrate that the glycolytic process and its derivative, lactylation modification, possess potential prognostic value in the context of immunochemotherapy. Utilizing ^18^F-FDG PET/CT to image glycolysis intensity, we reviewed a prospective cohort of pancreatic cancer patients who underwent ^18^F-FDG PET/CT prior to chemotherapy or immunochemotherapy. Patients were stratified into 2 groups based on SUVmax (35). Our findings indicate that patients with hyper-glycolysis (higher ^18^F-FDG uptake) exhibited a poor response to immunochemotherapy. While ^18^F-FDG PET/CT is typically employed to identify recurrence and metastasis, it is rarely used for initial diagnosis. Therefore, we considered paracentesis, which is more commonly employed in the initial diagnosis of pancreatic cancer. We assessed Pan-Kla expression in biopsy specimens using mIHC. A retrospective review of a previous RCT cohort revealed that patients with high Pan-Kla expression had poor survival outcomes following immunochemotherapy (3). Pan-Kla expression may serve as a predictor of immunotherapy efficacy, with an AUC of approximately 95.59.

We identified a glycolysis-dependent ENSA-K63la/SRC-pS12/STAT3-pY705 axis that promotes an immunosuppressive microenvironment. Based on this, we propose 3 potential targeted intervention strategies. First, targeting the aberrant glycolysis enhancements caused by KRAS mutations could be effective. Using the KRAS-G12D inhibitor MRTX1133 reduced glycolysis and tumor weight in vivo. Given that KRAS mutations are absent in normal cells, targeting mutant KRAS offers specificity and clinical potential. Second, ENSA-K63la could serve as a potential target. ENSA-K63 can undergo lactylation, inactivating PP2A, sustaining SRC-S12 phosphorylation, and promoting STAT3 constitutive activation. A cell-penetrating peptide designed to block ENSA-K63la could reduce STAT3-Y705 phosphorylation. This K63-peptide could potentially reshape the TME and enhance the sensitivity of pancreatic cancer to immunotherapy. Finally, pharmacologically targeting CCR2 might be a universal strategy for combination therapy. Our study found that serum CCL2 levels negatively correlate with overall survival and are associated with poor immunochemotherapy outcomes. Elevated tumor glycolysis attracts monocytes/macrophages via the ENSA-K63la/SRC-pS12/STAT3-pY705/CCL2 axis. Macrophages in the TME uptake lactate derived from the tumor, which further promotes the ENSA-K63la/SRC-pS12/STAT3-pY705 axis and increases CCL2 expression, enhancing the chemotactic effect on monocytes/macrophages. Additionally, STAT3 activation promotes the expression of CCL2, ARG1, S100A9, and IL10, contributing to an immunosuppressive microenvironment. Blocking the CCL2/CCR2 axis could reduce macrophage infiltration and inhibit the formation of protumor macrophages.

Radiology, pathology, serology, and genomics serve as pivotal pillars in achieving precision therapy. Techniques such as ^18^F-FDG PET/CT and Pan-Kla staining on biopsy samples allow for the identification of patients who may respond well to immunotherapy. Serological assessments can reveal individuals who might benefit from CCR2 inhibition. Additionally, genomic profiling can detect patients harboring specific KRAS mutations, making them ideal candidates for targeted therapies. By leveraging these advanced diagnostic tools, healthcare providers can tailor precise treatment plans to the unique genetic, metabolic, and molecular profiles of each patient, thereby optimizing therapeutic outcomes.

This study has several limitations. Although NALA supplementation can largely restore the downregulation of STAT3 phosphorylation caused by glycolysis inhibition, other factors in the glycolytic process may also influence STAT3 activation. Additionally, while ENSA-K63la is a key regulator of STAT3 activation, lactate may activate STAT3 phosphorylation through other pathways. In the future, we will further investigate the impact of glycolysis on the immune microenvironment of pancreatic cancer.

## Methods

### Sex as a biological variable.

We used male mice to perform animal experiments. Since estrogen could be a suppressive factor in tumor progression, male mice could be a universal model for cancer therapy. Both men and women were included in the clinical cohorts, there was no bias in the grouping, and there were consistent results for both men and women.

### Human tissues and patient cohorts.

Paraffin-embedded PDAC tissue array slides were created by Wuhan Servicebio Technology using in-house PDAC tissue specimens. Additionally, 4 patient cohorts were used in this research. Clinical data are shown in Supplemental Tables 1–4.

### PET/CT Imaging.

^18^F-FDG PET/CT scans were conducted using a PET/CT scanner (Siemens Healthineers). Image analysis followed the same methodology as previous studies (35). SUV was utilized to normalize tissue activity concentration, injected dose, and body weight. The SUVmax was used for analysis in this study.

### Cell culture.

The KPC cell line was a gift from the laboratory of Raghu Kalluri (Department of Cancer Biology at the University of Texas MD Anderson Cancer Center, Houston, Texas, USA). All other PDAC cell lines were purchased from ATCC.

BMDMs were obtained from C57BL/6 mice and cultured in RPMI medium containing M-CSF (40 ng/ml), 10% fetal bovine serum, and 1% penicillin and streptomycin at 37°C with 5% CO_2_. After approximately 3 days, once the cells were adherent, fresh RPMI complete medium containing KPC supernatant was added to induce TAMs, with a ratio of 1:1 for KPC supernatant to RPMI medium.

### Plasmid and cell transfection.

Hk2 short hairpin RNA was cloned into the pLKO.1-EGFP plasmid, while Ensa small guide RNA was cloned into the LentiCRISPRV2-GFP plasmid. Other plasmids used in this research were purchased from industry.

To create an *Ensa* knockout subclone, the LentiCRISPRV2-GFP plasmid was transiently transfected into the KPC cell line, which was then sorted into single cells using GFP cytometry and placed into 96-well plates. For stable transfection cell lines, lentivirus was used following the method described in previous studies (61). All transient and stable transfections were confirmed via Western blotting.

### Animal experiments.

Six-week-old male mice were each challenged by 1,000,000 KPC cells in a mixture of medium and Matrigel (1:1) in all experiments. For the orthotopic tumor model, KPC cells in 25 μl mixture was injected into the pancreas under anesthesia.

*HuCD34+HSC-NOD/ShiLtJGpt-Prkdc^em26Cd52^Il2rg^em26Cd22^/Gpt(CH)* mice were purchased from GemPharmatech Co. To generate PDX models, fresh tumor samples from patients during surgery were collected. A tumor fragment was inserted into the pocket created under the skin of a nude mice. Tumor growth was monitored, and once the tumor reached the desired size, the mouse was euthanized for tumor collection. Under sterile conditions, the tumor tissue was trimmed into small fragments (2–3 mm^3^) using sterile scissors or scalpels and then transplanted into NCG mice. Tumor growth was monitored and the mouse was euthanized for tumor collection. The samples were mechanically dissociated into small fragments using scissors and scalpels, then placed in DMEM supplemented with 2% FBS, collagenase IV (1 mg/ml), DNase (10 μg/ml), and EDTA (2 mM). They were incubated at 37°C with shaking at 90*g* for 30 minutes. Digestion was terminated by adding DMEM containing 10% FBS. The dissociated tissues were then filtered through a 40 μm cell strainer and washed with PBS. The tumor cells were resuspended in a mixture of PBS and Matrigel (1:1, 1,000,000 cells in 25 μl mixture) and injected into the flanks of *HuCD34+HSC-NOD/ShiLtJGpt-Prkdc^em26Cd52^Il2rg^em26Cd22^/Gpt(CH)* mice.

Doses of pharmacological treatment used in the study are as follows: anti–PD-1 mAb (BE0146, BioXcel, 100 μg/mouse, i.p., every t3 days), anti-CD8 mAb (BE0061, BioXcel, 100 μg/mouse, i.p., every 3 days), clodronate liposomes (40337ES08, Yeasen, i.p, once a week), MRTX1133 (HY-134813, MCE, 0.2 mg/mouse, i.p., once a day), PF-4136309 (HY-13245, MCE, 0.2 mg/mouse, i.p., every 2 days) and cell-penetrating peptide (0.2 mg/mouse, i.p., every 2 days). The mice were sacrificed, and the tumors were excised for weighing and further analysis. All of the mice that were alive were included in data analysis.

### Flow cytometry.

Sample pretreatment was the same as in previous studies (61). Samples were detected using a Fortessa flow cytometer (Becton Dickinson) and Cytek Aurora (Cytek Biosciences). Full spectrum flow cytometry was performed as guided (62). Data were analyzed using FlowJo software (Becton Dickinson, version 10.8.1).

### Western blotting and immunoprecipitation.

Cells and tissues were lysed using RIPA buffer containing a protease inhibitor cocktail and a phosphatase inhibitor cocktail. For immunoprecipitation, cell supernatants collected after treatment were incubated with anti-FLAG, anti-HA, or anti-MYC magnetic beads for 4 hours. All experiments were conducted following protocols from previous studies (61). The grayscale values of the WB bands were quantified using ImageJ (NIH). The relative expression was normalized to the control group.

### Quantitative real-time reverse transcription PCR analysis.

Cell total RNA was isolated and then reversed transcribed. Primers used are shown in Supplemental Table 7. The relative expression was normalized to that of ACTB/*Actb* (encoding β-actin) using the 2−ΔΔCt method.

### mIHC.

Multiplex IHC was performed using an Opal Polaris 7-Color Manual IHC Kit (NEL861001KT) following the provided protocol strictly. For follow-up quantitative analysis, InForm software (version 2.5; Akoya Biosciences) was used. Cells were adaptively segmented based on nuclear, cytoplasm and cell membrane markers. Cells were marked to develop a training classifier and then phenotype using machine learning. The proportion of each cell type in all cells was calculated. The histochemistry scores (H-score) of the total region were calculated. The mean fluorescence intensity of each cell type was calculated. For TMA, incomplete regions were excluded in analysis. Tumor regions where the proportion of tumor cells was less than 10% were excluded in analysis.

### ELISA Assay.

The concentration of CCL2 in serum or supernatant was quantified using an ELISA kit according to the manufacturer’s instruction. The numbers of the cells and the volume of the liquid were kept consistent.

### PP2A active enzyme quantification assay.

SRC-pS12 cell-penetrating peptide (HLYVSPWGG-SKPKDApSQRRRSL) was incubated in the KPC cell line for 24 hours before the test. The cellular PP2A Active Enzyme Quantification Assay Kit (JW50312.1, Chundubio) was used to perform this study.

### RNA-Seq analysis.

All samples were pretreated and analyzed by the Biomedical Big Data Center of the First Affiliated Hospital, School of Medicine, Zhejiang University. The raw data that passed quality control were subjected to differential expression and functional enrichment.

### Peptide synthesis.

All peptides were synthesized by Guoping Pharmaceutic Inc. The peptides used were all D isoforms, acetylated at the N-terminus, and amidated at the C-terminus. The HLYVSPWGG sequence was used to enhance cell penetration. Peptides were dissolved in PBS for use.

### Proteomics.

To identify protein lactylated, proteins from PDAC samples and KPC cell lines were isolated, trypsinized, and labeled with TMT. Enrichment was performed using anti-l-lactyllysine antibody–conjugated agarose beads.

To identify protein interaction, liquid chromatography–mass spectrometry (LC-MS) analysis was performed as previously reported (61).

### Bioinformatics analysis.

Considering the main differences focused on macrophages and T cells, we combined 4 public scRNA-Seq datasets (CRA001160, GSE205013, GSE212966, phs002371). After integration with scVI and annotation with typical markers (CD3D, CD3E for T cells; CD68, CD14, CD163, LYZ, MRC1, S100A9 for myeloid cells), 348,560 cells remained in primary tumor samples without any treatment (63). Next, myeloid and T cell subsets were further identified into 4 and 5 types, respectively. The glycolysis score of the epithelial subset was calculated using single-sample gene set enrichment analysis (ssGSEA), and patients were divided into 2 groups, glycolysis-high and glycolysis-low, based on the median average score (64). Grouped by patient classification, markers of macrophages and CD8^+^ T cells were identified using the FindAllMarkers function in Seurat (version 5.0.1) (65). Markers with pct.1 > 0.05, log2FC > 0.5, and adjusted *P* < 0.01 were considered potential signatures. Gene Ontology (GO) enrichment was performed using clusterProfiler (66).

To explore functional differences between the 2 identified classes, GSEA was conducted using hallmark gene sets from the human collection of MSigDB (gsea-msigdb.org) (67–69). Additionally, ssGSEA in the GSVA package was used to assess glycolysis levels with a manually collected gene set (SLC2A1, HK2, GPI, PFK, PGK, PKM, LDHA, ENO1, PKM, LDHA, SLC16A3) (70). The resulting matrix (glycolysis_scores) contains glycolysis enrichment scores for each sample, which can be further analyzed or visualized.

### Materials.

Main regents and detailed information involved in this study are shown in Supplemental Table 7.

### Statistics.

GraphPad Prism software (version 7.0; GraphPad Inc.) was used for statistical analyses. Data from biological replicates are presented as the mean ± SD. Differences between 2 groups were compared using 2-sided, 2-tailed Student’s *t* tests, while 1-way ANOVA was employed to analyze differences among 3 or more groups. Spearman’s rank correlation was used to compare 2 variables. The Kaplan-Meier method and the Gehan-Breslow-Wilcoxon test were used to analyze differences between survival curves. Receiver operating characterisic–AUC (ROC-AUC) curves were utilized to evaluate the efficiency of predictive models. Throughout the study, *P* < 0.05 was considered statistically significant.

### Study approval.

Human samples and clinical information were collected from the Department of Hepatobiliary and Pancreatic Surgery at the First Affiliated Hospital, School of Medicine, Zhejiang University. No prospective patient cohort was included in this study. All medical records and biological samples were obtained from previous clinical treatments. The study is exempt from informed consent. The study protocol received approval from the Institutional Review Board of the First Affiliated Hospital, School of Medicine, Zhejiang University (IIT20240588A). Animal experiments received approval from the Institutional Review Board of the First Affiliated Hospital, School of Medicine, Zhejiang University.

### Data availability.

Raw RNA-Seq data have been deposited in the National Genomics Data Center (GSA CRA018166). Sequencing data are part of the HTAN dbGaP under accession phs002371.v3.p1. These data are available through the HTAN DCC Portal (https://data.humantumoratlas.org/) under the HTAN WUSTL Atlas. This paper does not include any original code. Values for all data points in graphs are reported in the Supporting Data Values file.

## Author contributions

Kang Sun and X Zhang designed this research. Kang Sun, X Zhang, JH, X Li, M Ye, and Ke Sun supported the methodology of this research. Kang Sun, J Shi, SW, HL, DZ, SZ, LQ, M Yang, CL, ML, LH, J Song, NL, YJ, YC, SY, HY, LL, X Liu, JL, YM, JZ, QX, and X Zhi performed the experiments. Kang Sun, DZ, and JH visualized the results. XB, TL, X Zhang, and X Lu supervised this research. Kang Sun, X Zhang, XB, and X Lu wrote and revised this manuscript. Kang Sun, X Zhang, J Shi, and JH are co–first authors; authorship order reflects the degree to which authors drove key developments in the work.

## Supplementary Material

Supplemental data

Supporting data values

## Version 1 01/30/2025

In-Press Preview

## Version 2 04/01/2025

Electronic publication
