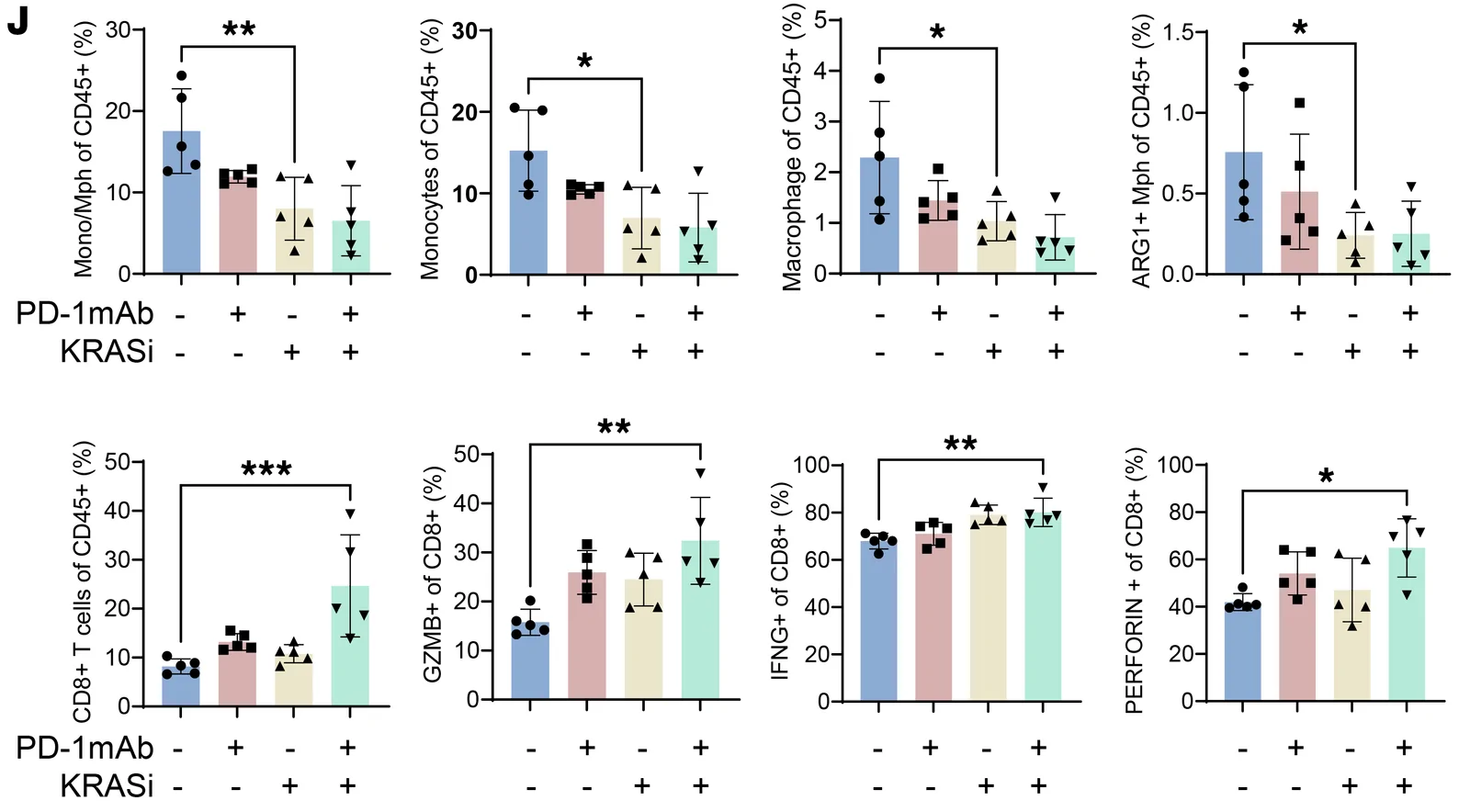# Corrigendum to Elevated protein lactylation promotes immunosuppressive microenvironment and therapeutic resistance in pancreatic ductal adenocarcinoma

**DOI:** 10.1172/JCI211838

**Published:** 2026-10-01

**Authors:** Kang Sun, Xiaozhen Zhang, Jiatao Shi, Jinyan Huang, Sicheng Wang, Xiang Li, Haixiang Lin, Danyang Zhao, Mao Ye, Sirui Zhang, Li Qiu, Minqi Yang, Chuyang Liao, Lihong He, Mengyi Lao, Jinyuan Song, Na Lu, Yongtao Ji, Hanshen Yang, Lingyue Liu, Xinyuan Liu, Yan Chen, Shicheng Yao, Qianhe Xu, Jieru Lin, Yan Mao, Jingxing Zhou, Xiao Zhi, Ke Sun, Xiongbin Lu, Xueli Bai, Tingbo Liang

Original citation: *J Clin Invest*. 2025;135(7):e187024. https://doi.org/10.1172/JCI187024

Citation for this corrigendum: *J Clin Invest*. 2026;136(19):e211838. https://doi.org/10.1172/JCI211838

After publication of this article, the authors became aware of the following errors. In Figure 2J, the graph reporting Mono/Mph of CD45+ (%) was inadvertently duplicated in the top two left panels. The second panel has been replaced to show Monocytes of CD45+ (%). The corrected figure is shown below.

For Supplemental Figure 7G, the data for one sample row (H-score, 56; Pan-Kla, 0.021; CCL2, 0.275; ARG1, 0.082; S100A9, 2.331) were inadvertently duplicated. These data have been deleted and the sample size corrected to n = 17. The recalculated Pearson’s correlations are: Pan-Kla H-score versus Pan-Kla MFI on macrophages, r = 0.9024, P < 0.0001; Pan-Kla MFI versus CCL2 MFI, r = 0.7986, P < 0.0001; Pan-Kla MFI versus ARG1 MFI, r = 0.8046, P < 0.0001; and Pan-Kla MFI versus S100A9 MFI, r = 0.4976, P = 0.0421. The direction and statistical interpretation of the correlations are unchanged.

The HTML and PDF versions of the article, as well as the Supplemental data and Supporting data values files, have been updated online.

The authors regret the errors.

## Figures and Tables

**Figure 2J:**