# Supplementary material for: Elevated protein lactylation promotes immunosuppressive microenvironment and therapeutic resistance in pancreatic ductal adenocarcinoma
Source: J Clin Invest. 2025 Jan 30;135(7):e187024. doi: 10.1172/JCI187024 (PMC11957693; doi:10.1172/JCI187024)
Supplement: Supplemental data [file jci-135-187024-s137.pdf]

Elevated protein lactylation promotes immunosuppressive microenvironment and therapeutic resistance in pancreatic ductal adenocarcinoma

Kang Sun, Xiaozhen Zhang, Jiatao Shi, Jinyan Huang, Sicheng Wang, Xiang Li, Haixiang Lin, Danyang Zhao, Mao Ye, Sirui Zhang, Li Qiu, Minqi Yang, Chuyang Liao, Lihong He, Mengyi Lao, Jinyuan Song, Na Lu, Yongtao Ji, Hanshen Yang, Linyue Liu, Xinyuan Liu, Yan Chen, Shicheng Yao, Qianhe Xu, Jieru Lin, Yan Mao, Jingxing Zhou, Ke Sun, Xiongbin Lu, Xueli Bai, Tingbo Liang

Supplemental information

Supplemental Figures and Legends

Supplementary Figure 1. Elevated lactylation is correlated with immunosuppressive TME in PDAC (related to Figure 1).

Supplementary Figure 2. Elevated lactylation is associated with

immunotherapy resistance in PDAC (related to Figure 2).

Supplementary Figure 3. Inhibiting glycolysis reduces the levels of CCL2 secreted by tumor (related to Figure 3).

Supplementary Figure 4. Inhibition of glycolysis process sensitized immunotherapy of PDAC (related to Figure 3).

Supplementary Figure 5. ENSA could be a regulator of STAT3 phosphorylation (related to Figure 4).

Supplementary Figure 6. Targeting ENSA-K63la activated immune microenvironment (related to Figure 5).

Supplementary Figure 7. Lactate accumulation reprograms TAMs by ENSA lactylation (Related to Figure 6)

Supplementary Figure 8. ENSA-K63-pe, CCR2i, and MRTX1133 enhance the sensitivity of pancreatic cancer to anti-PD1 or anti-CTLA4 immunotherapy (Related to Figure 7)

Supplemental Tables

Supplementary Table 1. Clinical information of cohort 1

Supplementary Table 2. Clinical information of cohort 2

Supplementary Table 3. Clinical information of cohort 3

Supplementary Table 4. Clinical information of cohort 4

Supplementary Table 5. Lactylated proteins in human PDAC samples and KPC cell lines

Supplementary Table 6. Proteins interacted with PPP2CA

Supplementary Table 7. Detailed information of reagent used in this research

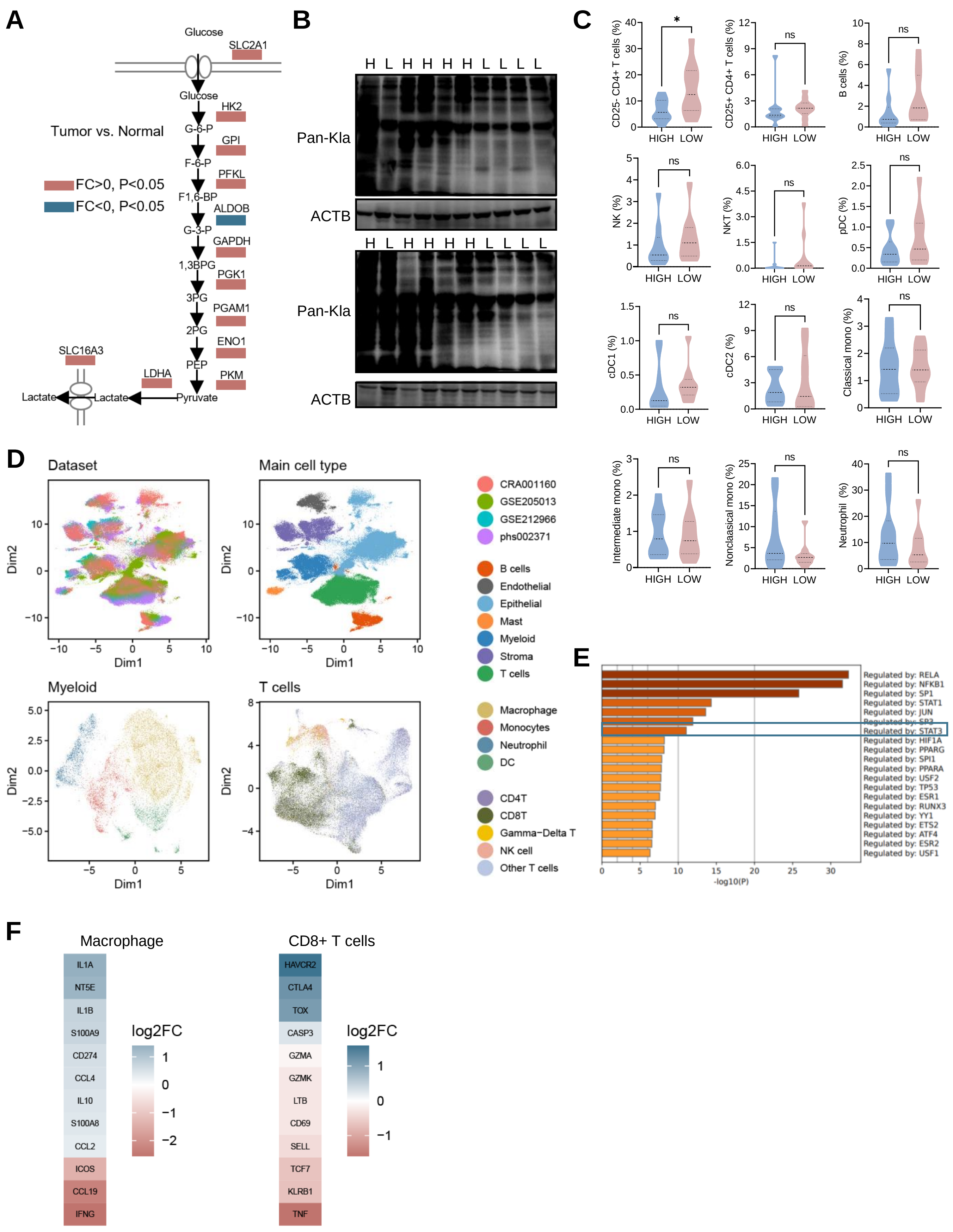
A
Glucose
SLC2A1
Glucose
HK2
G-6-P
GPI
F-6-P
PFKL
F1,6-BP
ALDOB
G-3-P
GAPDH
1,3BPG
PGK1
3PG
PGAM1
2PG
ENO1
PEP
PKM
Pyruvate
LDHA
Lactate
SLC16A3
Lactate
Tumor vs. Normal
FC>0, P<0.05
FC<0, P<0.05
B
H L H H H H L L L L
Pan-Kla
ACTB
H L H H H H L L L L
Pan-Kla
ACTB
C
CD25- CD4+ T cells (%)
CD25+ CD4+ T cells (%)
B cells (%)
NK (%)
NKT (%)
pDC (%)
cDC1 (%)
cDC2 (%)
Classical mono (%)
Intermediate mono (%)
Nonclaasical mono (%)
Neutrophil (%)
HIGH LOW
*
ns
D
Dataset
Main cell type
Myeloid
T cells
Dim1
Dim2
CRA001160
GSE205013
GSE212966
phs002371
B cells
Endothelial
Epithelial
Mast
Myeloid
Stroma
T cells
Macrophage
Monocytes
Neutrophil
DC
CD4T
CD8T
Gamma-Delta T
NK cell
Other T cells
E
Regulated by: RELA
Regulated by: NFKB1
Regulated by: SP1
Regulated by: STAT1
Regulated by: JUN
Regulated by: SP3
Regulated by: STAT3
Regulated by: HIF1A
Regulated by: PPARG
Regulated by: SPI1
Regulated by: PPARA
Regulated by: USF2
Regulated by: TP53
Regulated by: ESR1
Regulated by: RUNX3
Regulated by: YY1
Regulated by: ETS2
Regulated by: ATF4
Regulated by: ESR2
Regulated by: USF1
-log10(P)
F
Macrophage
IL1A
NT5E
IL1B
S100A9
CD274
CCL4
IL10
S100A8
CCL2
ICOS
CCL19
IFNG
log2FC
CD8+ T cells
HAVCR2
CTLA4
TOX
CASP3
GZMA
GZMK
LTB
CD69
SELL
TCF7
KLRB1
TNF
log2FC

Supplementary Figure 1 Elevated lactylation is correlated with immunosuppressive TME in PDAC (related to Figure 1)

(**A**) Flowchart of glycolysis process. Compared with normal, changes of glycolysis-related genes in tumor were marked in flowchart. TCGA-PAAD dataset was used for analysis.

(**B**) Frozen tissues of 20 fresh pancreatic tumor samples performed Western blotting analysis. Samples were divided into two groups according to Immunoblotting band intensity of Pan-Kla.

(**C**) 20 fresh pancreatic cancer samples were process into a single-cell suspension and performed flow cytometry analysis using Cytek. Samples were divided into two groups according to expression analysis in **B**. Statistical analyses were performed with Student's t test (n=20).

(**D**) Single-cell RNAseq data processing workflow. Four public single-cell RNA sequencing datasets (CRA001160, GSE205013, GSE212966, phs002371) were included in follow-up analysis. After integration with scVI and annotation with typical markers, eight main cell types and 348,560 cells remained in primary tumor samples without any treatment. Next, myeloid and T cell subsets were further identified into different cell types, respectively.

(**E**) Transcriptional factor analysis of single-cell macrophage expression profile. Top 3000 upregulated genes of macrophages from high glycolysis tumor were analyzed using matascape.

(**F**) Differential genes of CD8+ T cells and macrophages in single-cell expression profile. Patients were categorized into two groups based on the median of glycolysis scores in epithelial cells and extracted the transcriptomic data of macrophages and $CD8^+$ T cells from these patients. Log2 fold change of key genes related to tumor immunity was shown.

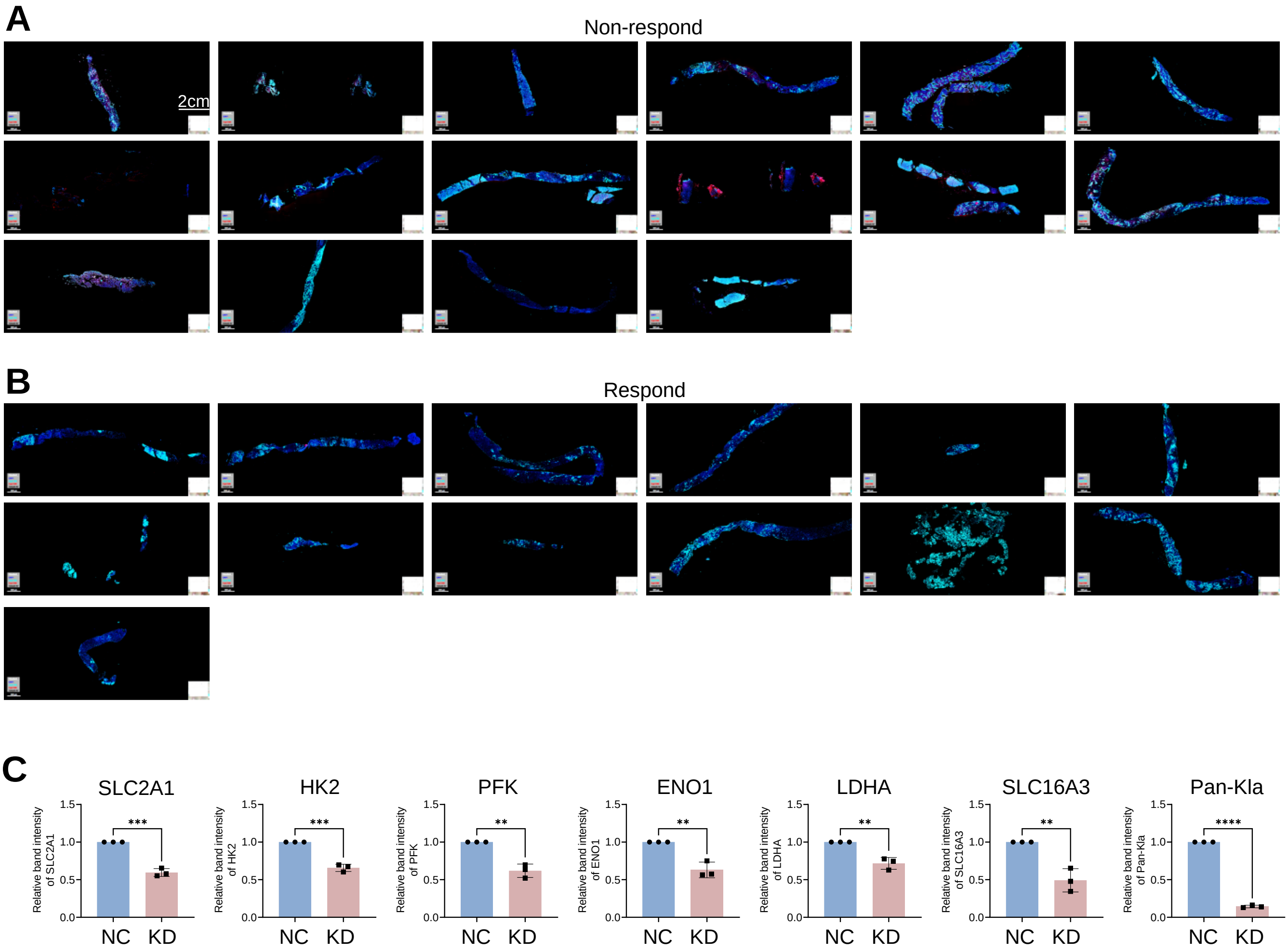

A
Non-respond
2cm
B
Respond
C
SLC2A1
HK2
PFK
ENO1
LDHA
SLC16A3
Pan-Kla
Relative band intensity of SLC2A1
Relative band intensity of HK2
Relative band intensity of PFK
Relative band intensity of ENO1
Relative band intensity of LDHA
Relative band intensity of SLC16A3
Relative band intensity of Pan-Kla
0.0
0.5
1.0
1.5
***
**
****
NC
KD

Supplementary Figure 2 Elevated lactylation is associated with immunotherapy resistance in PDAC (related to Figure 2)
(**A-B**) mIHC staining of pancreatic cancer biopsy specimens. PANCK (cyan) and Pan-Kla (red) were stained on 31 biopsy specimens. Images of patients not responded to immunochemotherapy (progressive disease and stable disease) (**A**) and responded to immunochemotherapy (partial respond and complete respond) (**B**) were shown. Scale bar=2cm.
(**C**) Relative band intensity analysis of **Figure 2G**

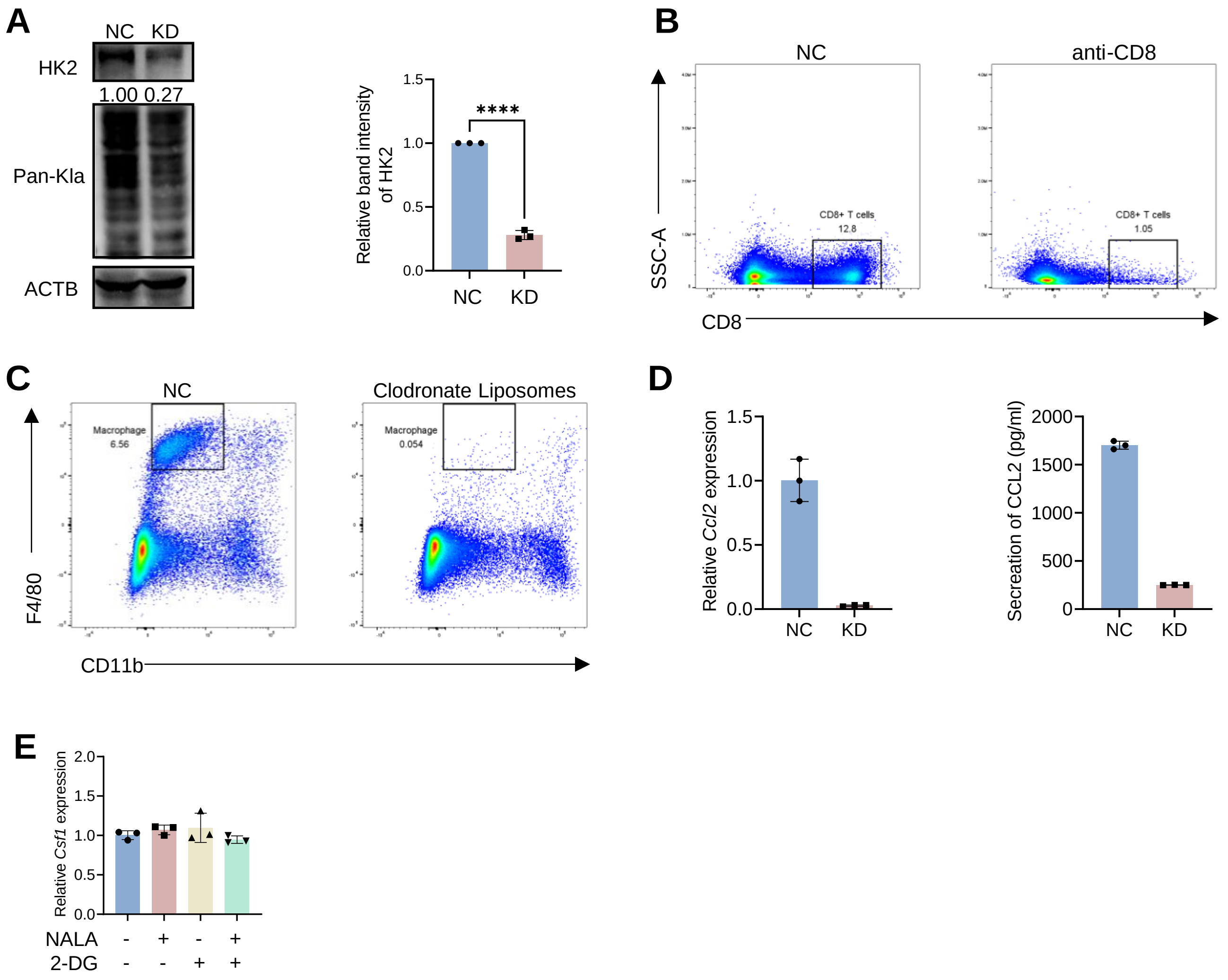

A
NC KD
HK2
1.00 0.27
Pan-Kla
ACTB
Relative band intensity of HK2
****
NC KD
B
NC
anti-CD8
SSC-A
CD8
CD8+ T cells
12.8
CD8+ T cells
1.05
C
NC
Clodronate Liposomes
Macrophage
6.56
Macrophage
0.054
F4/80
CD11b
D
Relative Ccl2 expression
NC KD
Secreation of CCL2 (pg/ml)
NC KD
E
Relative Csf1 expression
NALA - + - +
2-DG - - + +

Supplementary Figure 3 Inhibiting glycolysis reduces the levels of CCL2 secreted by tumor (related to Figure 3)

(**A**) *Hk*2-KD was verified by Western blotting analysis and Relative band intensity analysis was performed. Pan-Kla expression in *Hk*2-KD KPC cell line was decreased compared with *Hk*2-NC KPC cell line.

(**B**) The effect of anti-CD8mAb in vivo was proved by flow cytometry analysis. Anti-CD8a antibody was used to verify the effect of $CD8^+$ T cells clearance.

(**C**) The effect of Clodronate Liposomes was proved by flow cytometry analysis. Anti-CD11b and anti-F4/80 antibody was used to verify the effect of macrophage clearance.

(**D**) Relative *Ccl2* mRNA expression and CCL2 secretion tested by ELISA was shown with three technical replicates. *Hk*2-KD KPC cell line was compared with *Hk*2-NC KPC cell line.

(**E**) Relative *Csf1r* mRNA expression was shown with three technical replicates. KPC cells was treated by 2-DG (10mM, 24h) or NALA (40mM, 24h).

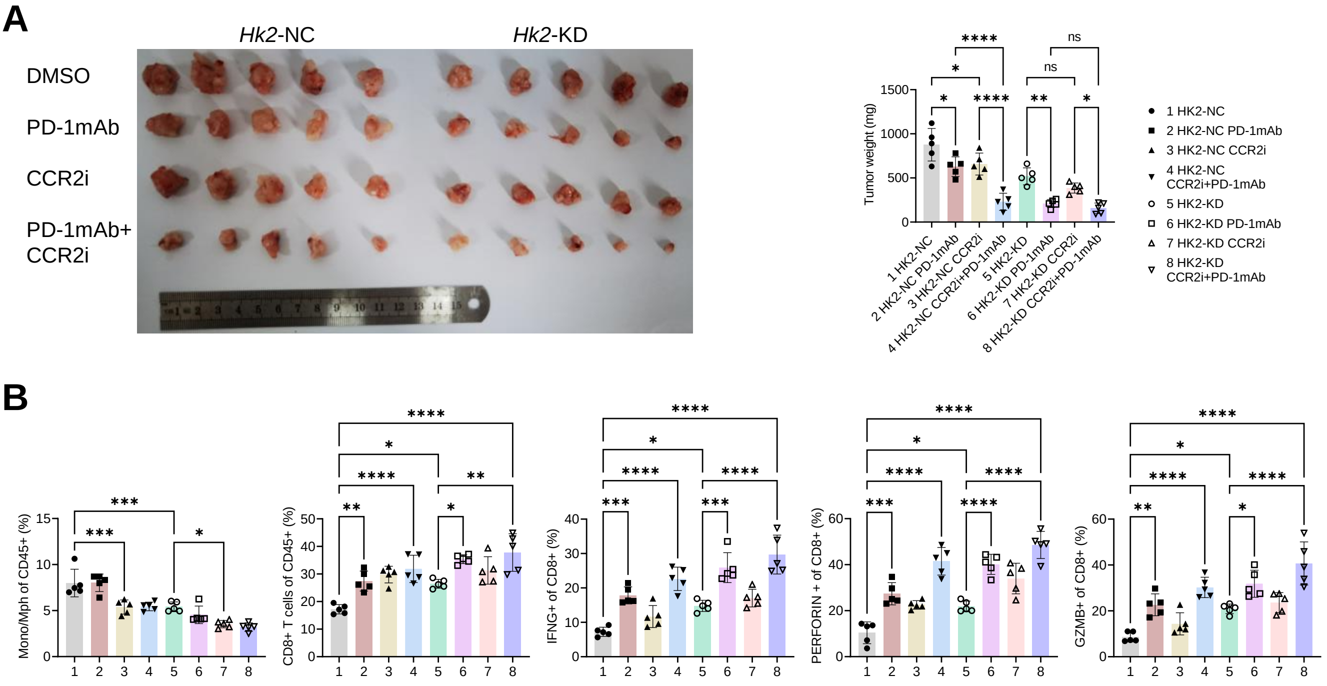
A
Hk2-NC
Hk2-KD
DMSO
PD-1mAb
CCR2i
PD-1mAb+
CCR2i
Tumor weight (mg)
1 HK2-NC
2 HK2-NC PD-1mAb
3 HK2-NC CCR2i
4 HK2-NC CCR2i+PD-1mAb
5 HK2-KD
6 HK2-KD PD-1mAb
7 HK2-KD CCR2i
8 HK2-KD CCR2i+PD-1mAb
ns
B
Mono/Mph of CD45+ (%)
CD8+ T cells of CD45+ (%)
IFNG+ of CD8+ (%)
PERFORIN + of CD8+ (%)
GZMB+ of CD8+ (%)

Supplementary Figure 4 Inhibition of glycolysis process sensitized immunotherapy of PDAC (related to Figure 3)

(**A**) Orthotopic transplantation tumors and statistical analysis were shown. *Hk*2-NC and *Hk*2-KD KPC orthotopic transplantation models (1000000 cells in 25μl matrigel mixture) were individually treated by anti-PD1mAb (100 μg/mouse, i.p., tid) or PF-4136309 (CCR2 inhibitor, 0.2 mg/mouse, i.p., qd). Statistical analyses were performed with One-way Anova (n=5).

(**B**) Proportions of each cell-types in immunocytes ($CD45^+$ live cells) and function of $CD8^+$ T cells. The immune micro-environment of orthotopic transplantation model with different treatments were compared. Statistical analyses were performed with One-way Anova (n=5).

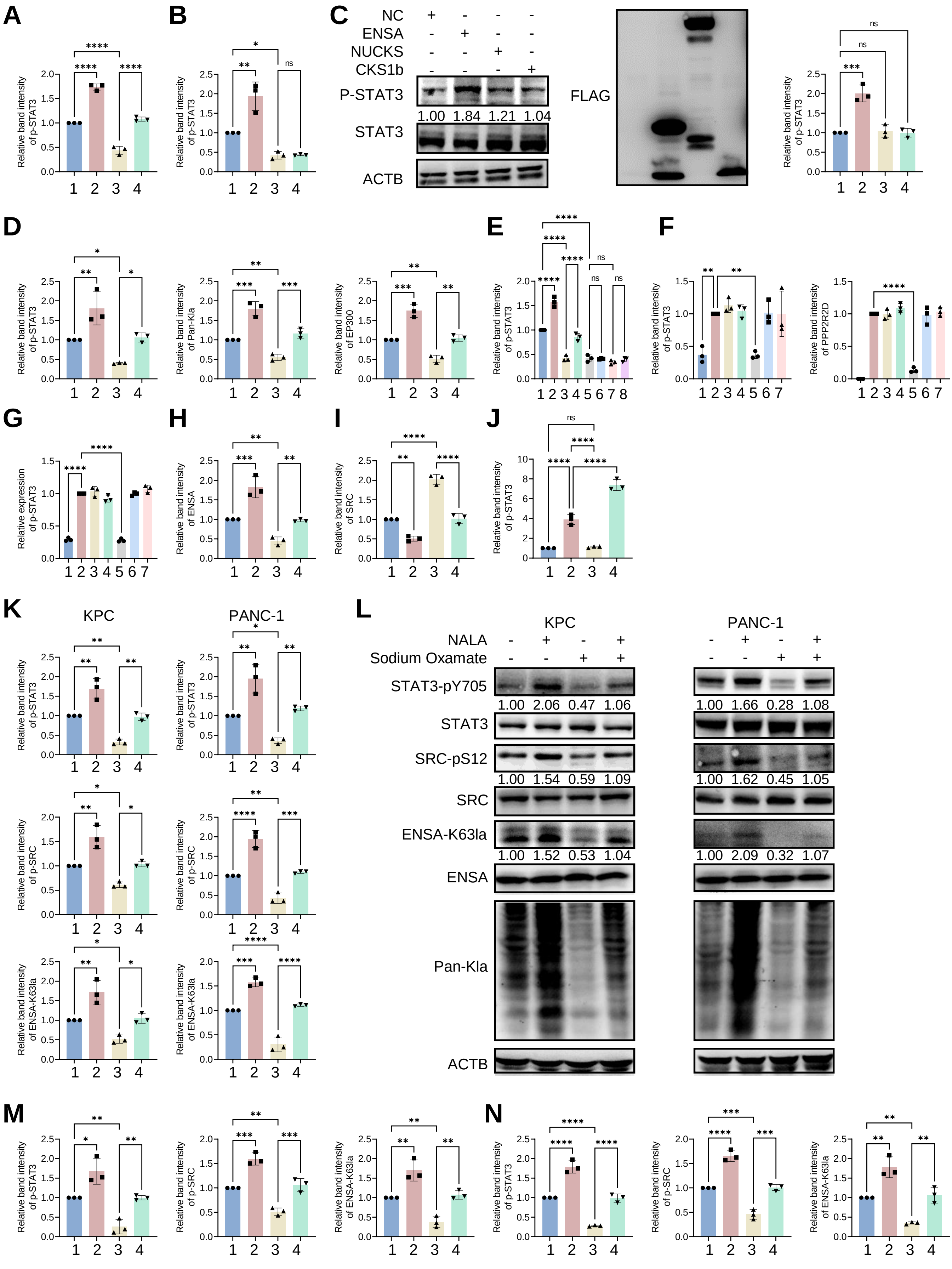
A
B
C
NC
ENSA
NUCKS
CKS1b
P-STAT3
STAT3
ACTB
1.00 1.84 1.21 1.04
FLAG
D
E
F
G
H
I
J
K
KPC
PANC-1
L
NALA
Sodium Oxamate
STAT3-pY705
1.00 2.06 0.47 1.06
1.00 1.66 0.28 1.08
SRC-pS12
1.00 1.54 0.59 1.09
1.00 1.62 0.45 1.05
SRC
ENSA-K63la
1.00 1.52 0.53 1.04
1.00 2.09 0.32 1.07
ENSA
Pan-Kla
M
N
Relative band intensity of p-STAT3
Relative band intensity of Pan-Kla
Relative band intensity of EP300
Relative band intensity of PPP2R2D
Relative expression of p-STAT3
Relative band intensity of ENSA
Relative band intensity of SRC
Relative band intensity of p-SRC
Relative band intensity of ENSA-K63la

Supplementary Figure 5 ENSA could be a regulator of STAT3 phosphorylation (related to Figure 4)

(A) Relative band intensity analysis of **Figure 4B**.

(**B**) Relative band intensity analysis of **Figure 4D**.

(**C**) Protein expression analysis of KPC cells with ENSA, NUCKS or CKS1b overexpression.

(**D**) Relative band intensity analysis of **Figure 4E**.

(**E**) Relative band intensity analysis of **Figure 4F**.

(**F**) Relative band intensity analysis of **Figure 4G**.

(**G**) The relative concentrations of p-STAT3 were measured using ELISA, and the results were normalized to the ENSA-WT group. 1: control, 2: ENSA-WT, 3: ENSA-K40R, 4: ENSA-K56R, 5: ENSA-K63R, 6: ENSA-K74R, 7: ENSA-K80R.

(**H**) Relative band intensity analysis of **Figure 4J**.

(**I**) Relative band intensity analysis of **Figure 4K**.

(**J**) Relative band intensity analysis of **Figure 4L**.

(**K**) Relative band intensity analysis of **Figure 4N**.

(**L**) Protein expression analysis of KPC and PANC-1 cells treated with Oxamate (20mM, 24h) or NALA (40mM, 24h).

(**M**) Relative band intensity analysis (KPC cells) of **Figure 4L**.

(**N**) Relative band intensity analysis (PANC-1 cells) of **Figure 4L**.

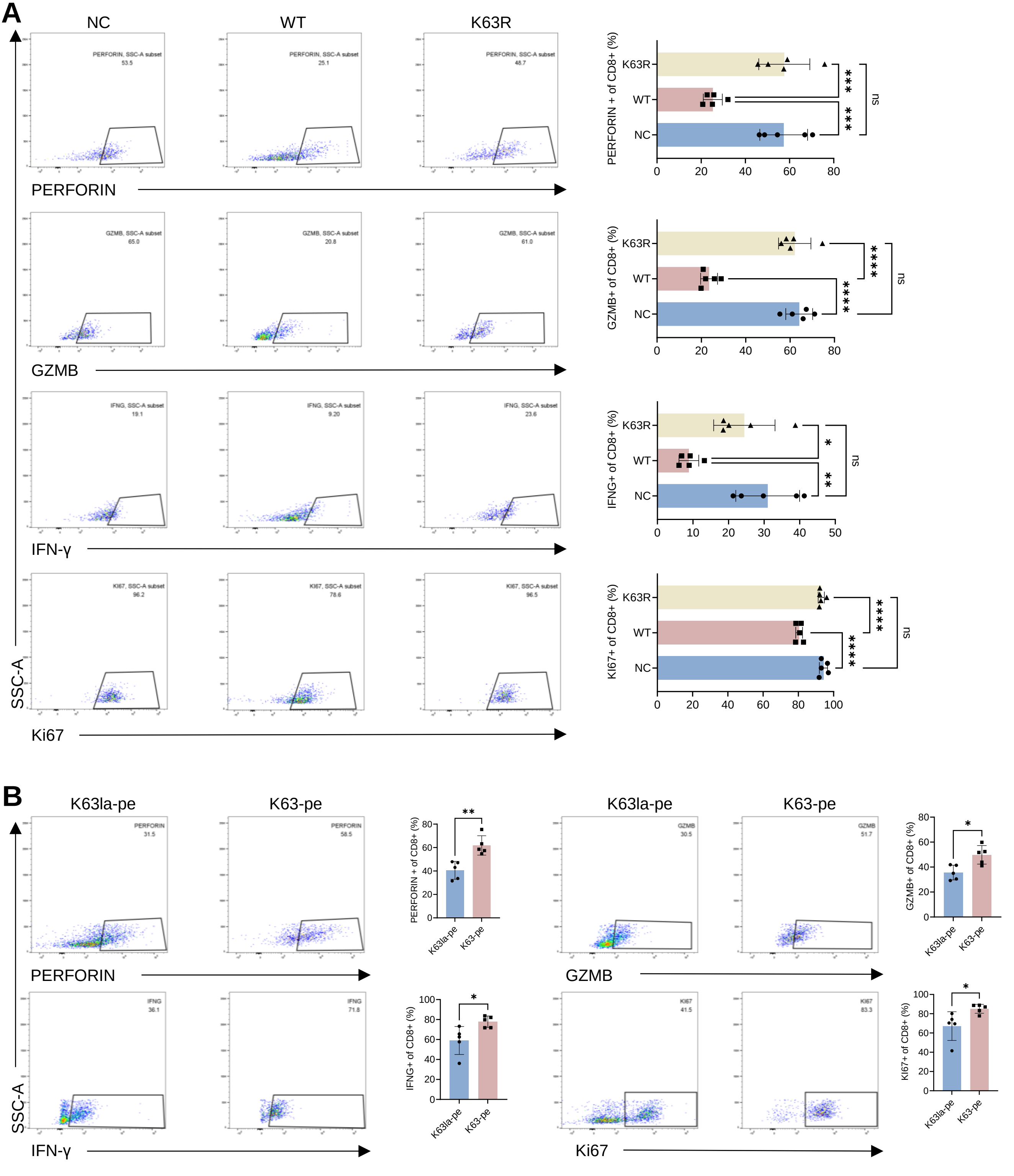
A
NC
WT
K63R
PERFORIN, SSC-A subset
53.5
PERFORIN, SSC-A subset
25.1
PERFORIN, SSC-A subset
48.7
PERFORIN
GZMB, SSC-A subset
65.0
GZMB, SSC-A subset
20.8
GZMB, SSC-A subset
61.0
GZMB
IFNG, SSC-A subset
19.1
IFNG, SSC-A subset
9.20
IFNG, SSC-A subset
23.6
IFN-γ
KI67, SSC-A subset
96.2
KI67, SSC-A subset
78.6
KI67, SSC-A subset
96.5
Ki67
SSC-A
PERFORIN + of CD8+ (%)
GZMB+ of CD8+ (%)
IFNG+ of CD8+ (%)
KI67+ of CD8+ (%)
K63R
WT
NC
***
ns
****
*
**
B
K63la-pe
K63-pe
PERFORIN
31.5
PERFORIN
58.5
GZMB
30.5
GZMB
51.7
IFNG
36.1
IFNG
71.8
KI67
41.5
KI67
83.3
PERFORIN
GZMB
IFN-γ
Ki67
SSC-A
PERFORIN + of CD8+ (%)
GZMB+ of CD8+ (%)
IFNG+ of CD8+ (%)
KI67+ of CD8+ (%)
K63la-pe
K63-pe
**
*

Supplementary Figure 6 Targeting ENSA-K63la activated immune microenvironment (related to Figure 5)

(**A**) Proportions of each functional marker in $CD8^+$ T cells. *Ensa*-KO KPC cells stably expressing vector control, ENSA-WT and ENSA-K63R (1,000,000 cells in 25μl matrigel mixture) were orthotopically transplanted into mice. The function of CD8+ T cells in tumor was tested by flow cytometry using Fortessa. Statistical analyses were performed with One-way Anova (n=5).

(**B**) Proportions of each functional marker in CD8+ T cells. The function of CD8+ T cells in KPC orthotopically transplanted tumor treated with K63la-pe control 3 or K63-pe inhibitor 3 were compared. Statistical analyses were performed with Student's t test (n=5).

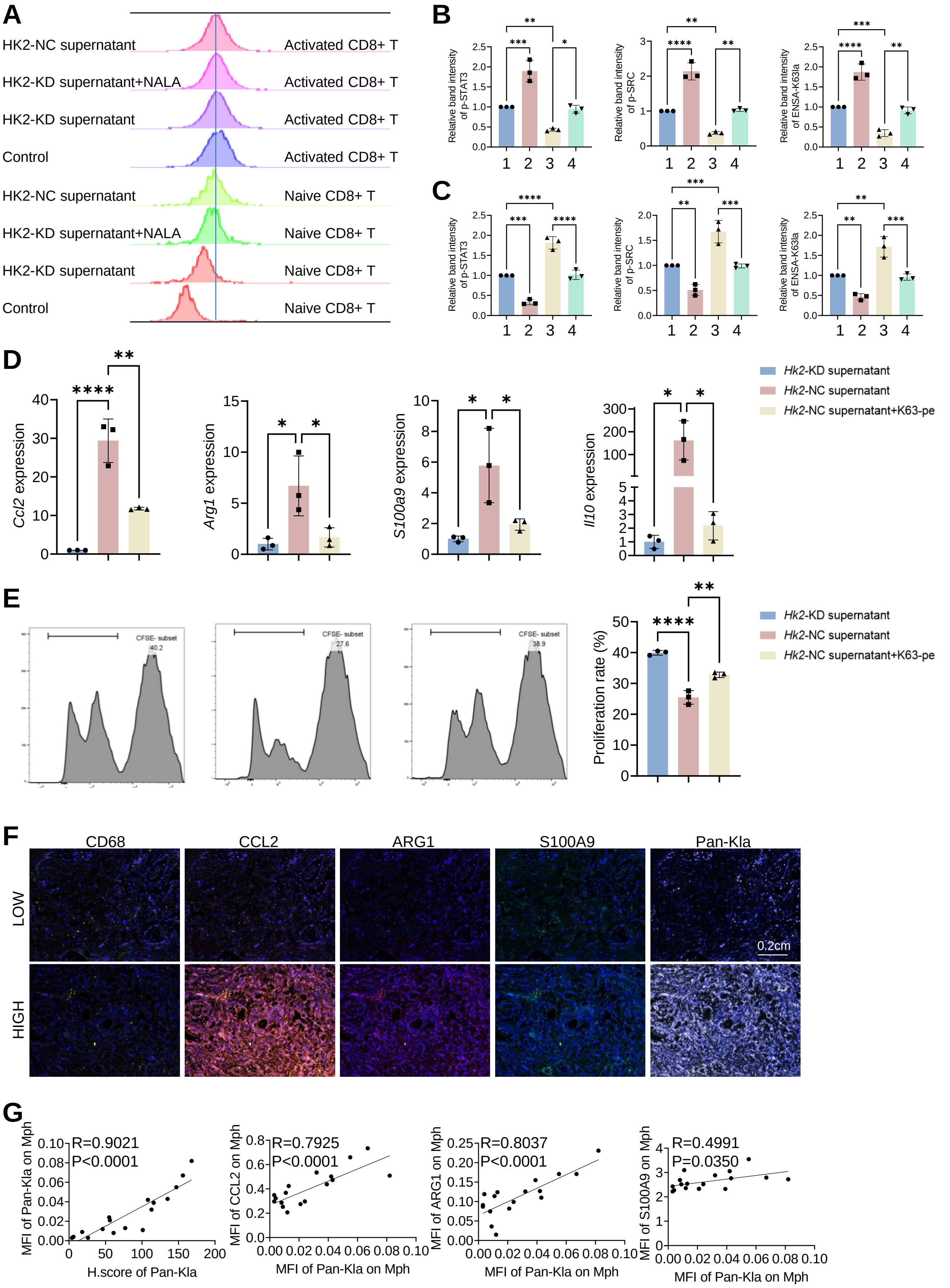
A
HK2-NC supernatant
HK2-KD supernatant+NALA
HK2-KD supernatant
Control
Activated CD8+ T
Naive CD8+ T
B
Relative band intensity of p-STAT3
Relative band intensity of p-SRC
Relative band intensity of ENSA-K63la
C
D
Ccl2 expression
Arg1 expression
S100a9 expression
Il10 expression
Hk2-KD supernatant
Hk2-NC supernatant
Hk2-NC supernatant+K63-pe
E
CFSE- subset
40.2
27.6
38.9
Proliferation rate (%)
F
CD68
CCL2
ARG1
S100A9
Pan-Kla
LOW
HIGH
0.2cm
G
R=0.9021
P<0.0001
MFI of Pan-Kla on Mph
H.score of Pan-Kla
R=0.7925
P<0.0001
MFI of CCL2 on Mph
R=0.8037
P<0.0001
MFI of ARG1 on Mph
R=0.4991
P=0.0350
MFI of S100A9 on Mph

Supplementary Figure 7 Lactate accumulation reprograms TAMs by ENSA lactylation (Related to Figure 6)

(**A**) Pan-Kla was tested using intracellular flow cytometry in naive $CD8^+$ T cells ($CD45^+CD3^+CD8^+CD25^-$) and activated $CD8^+$ T cells ($CD45^+CD3^+CD8^+CD25^+$) treated with conditioned media from control, Hk2-NC KPC cell cultures, Hk2-KD KPC cell cultures and Hk2-KD KPC cell cultures with 40 μM NALA for 24 hours

(**B**)Relative band intensity analysis of **Figure 6B**.

(**C**)Relative band intensity analysis of **Figure 6C**.

(**D**) Relative *Ccl2, Arg1, S100A9* and *Il10* mRNA expression was shown with three technical replicates. BMDM were pretreated with *Hk2*-NC and *Hk2*-KD KPC supernatant (1:1, 24h) and then treated with K63-pe inhibitor 3.

(**E**) $CD8^+$ T cells proliferation analysis. CFSE was used to analyze $CD8^+$ T cells proliferation. BMDM were pretreated with *Hk2*-NC and *Hk2*-KD KPC supernatant (1:1, 24h) and then treated with K63-pe inhibitor 3. CD8+ T cells were stained by CFSE (5 μM, 37℃, 20mins) and then cocultured with pretreated BMDM for two days.

(**F**) Representative images of mIHC of human PDAC paraffin sections. Anti-CD68, anti-ARG1, anti-S100A9, anti-CCL2 and anti-Pan-Kla antibodies were used for immunohistochemistry staining. Representative images of paraffin sections with high or low Pan-Kla expression were shown

(**G**) Simple linear regression was used to reveal correlation of CCL2, ARG1, S100A9 and Pan-Kla in human PDAC paraffin sections(n=18).

A

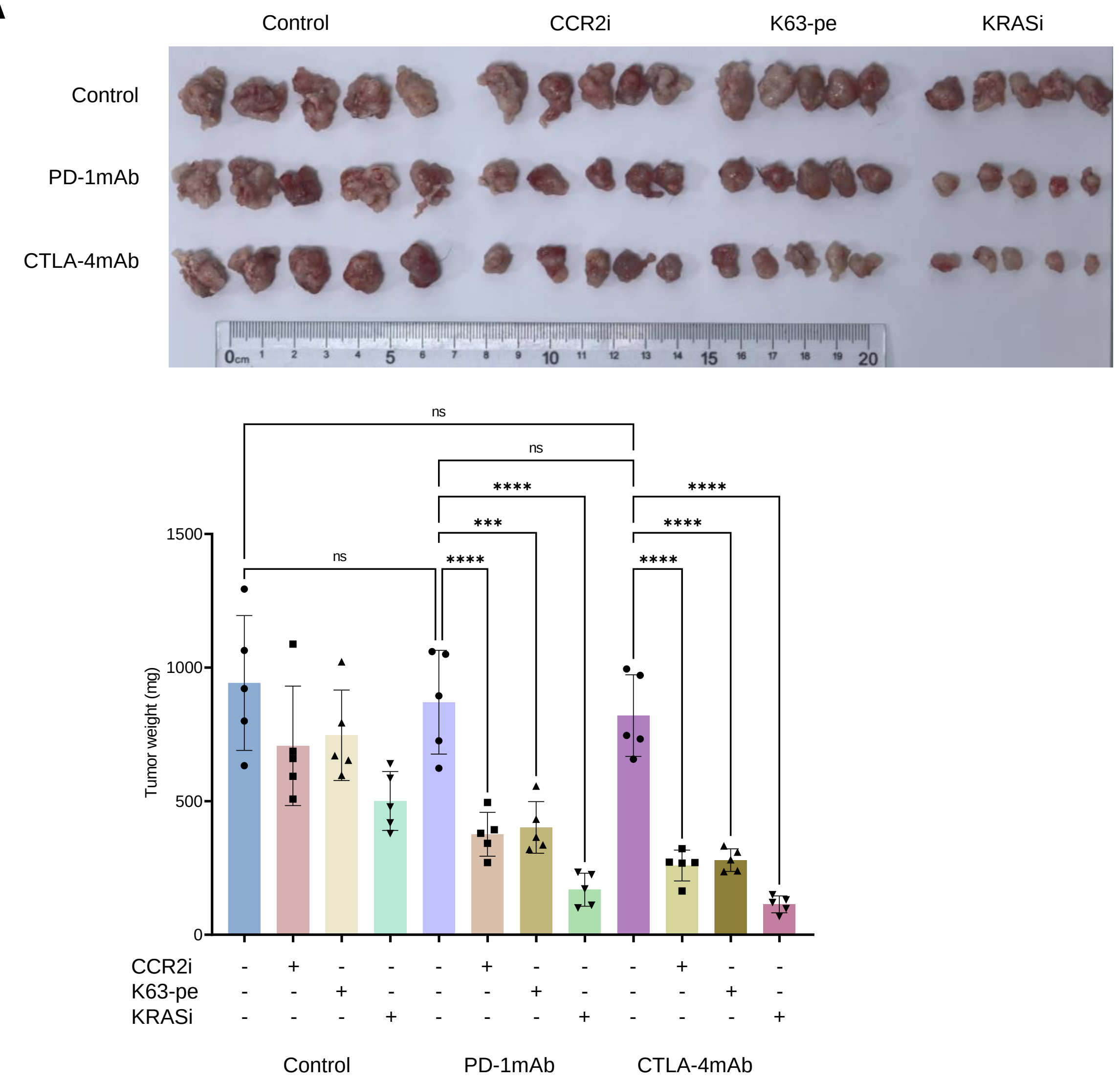
Control
CCR2i
K63-pe
KRASi
Control
PD-1mAb
CTLA-4mAb
ns
ns
****
***
****
****
****
****
ns
1500
1000
500
0
Tumor weight (mg)
CCR2i
K63-pe
KRASi
Control
PD-1mAb
CTLA-4mAb

Supplementary Figure 8. ENSA-K63-pe, CCR2i, and MRTX1133 enhance the sensitivity of pancreatic cancer to anti-PD1 or anti-CTLA4 immunotherapy (Related to Figure 7)

(A) Orthotopic transplantation tumors (1000000 cells in 25μl matrigel mixture) and statistical analysis were shown. KPC orthotopic transplantation mice (1,000,000 cells in 25μl matrigel mixture) were treated with anti-PD1 mAb (100 μg/mouse, i.p., tid), anti-CTLA-4 mAb (100 μg/mouse, i.p., tid), K63-pe inhibitor 3 (0.2 mg/mouse, i.p., qd), CCR2i (0.2 mg/mouse, i.p., qd) and MRTX1133 CCR2i (0.2 mg/mouse, i.p., qd). Drug treatment was initiated when tumors were palpable. Two days after the fifth injection of anti-PD1 mAb or anti-CTLA-4 mAb, the mice were euthanized, and the tumors were extracted. Tumor growth was analyzed with One-way Anova (n=5).

Supplementary Table 7 Detailed information of reagent used in this research

REAGENT or RESOURCE	SOURCE	IDENTIFIER
Antibodies		
BUV661 Rat Anti-Mouse CD4(GK1.5)	BD biosciences	612974
BUV737 Hamster Anti-Mouse CD11c(HL3)	BD biosciences	612796
BUV805 Rat Anti-Mouse CD8a(53-6.7)	BD biosciences	612898
Ms CD19 BUV563 1D3 50	BD biosciences	749028
BD Horizon™ BUV395 Rat Anti-CD11b	BD biosciences	563553
Brilliant Violet 421™ anti-mouse I-A/I-E Antibody	Biolegend	107632
Alexa Fluor® 700 anti-mouse CD45 Antibody	Biolegend	103128
Brilliant Violet 711™ anti-mouse CD206 (MMR) Antibody	Biolegend	141727
FITC anti-mouse Ly-6G Antibody	Biolegend	127606
APC/Fire™ 810 anti-mouse F4/80 Antibody	Biolegend	123166
Brilliant Violet 785™ anti-mouse CD25 Antibody	Biolegend	102051
APC anti-mouse Ly-6C Antibody	Biolegend	128016
Brilliant Violet 605™ anti-mouse Ly-6C Antibody	Biolegend	128036
Brilliant Violet 605™ anti-mouse IFN-γ Antibody	Biolegend	505839
Brilliant Violet 785™ anti-mouse CD45 Antibody	Biolegend	103149
FITC anti-mouse CD3ε Antibody	Biolegend	100204
PerCP/Cyanine5.5 anti-human/mouse Granzyme B Recombinant Antibody	Biolegend	372212
PE anti-mouse Perforin Antibody	Biolegend	154306
PE/Cyanine7 anti-mouse CD8a Antibody	Biolegend	100722
FOXP3 Monoclonal Antibody (FJK-16s), PE, eBioscience™	Thermo	12-5773-82
CD86 (B7-2) Monoclonal Antibody (GL1), PE-Cyanine5, eBioscience™	Thermo	15-0862-82
Arginase 1 Monoclonal Antibody (A1exF5), PE-Cyanine7, eBioscience™	Thermo	25-3697-82
Ki-67 Monoclonal Antibody (SolA15), Brilliant Ultra Violet™ 737, eBioscience™	Thermo	367-5698-82
PE/Cyanine7 anti-human IFN-γ Antibody	Biolegend	502528
PE anti-human Perforin Antibody	Biolegend	308106
APC/Cyanine7 anti-human CD3 Antibody	Biolegend	344818
FITC anti-human CD8	Biolegend	980908

PE anti-mouse/rat/human MCP-1 Antibody	Biolegend	505904
Brilliant Violet 421™ anti-human IL-10 Antibody	Biolegend	501422
Brilliant Violet 650™ anti-human CD4 Antibody	Biolegend	344692
APC/Cyanine7 anti-human CD14 Antibody	Biolegend	325620
BD Horizon™ Fixable Viability Stain 450	BD Bioscience	562247
LIVE/DEAD BLUE	Thermo	L23105
PE anti-STAT3 Phospho (Tyr705) Antibody	Biolegend	505910
APC anti-mouse/rat/human MCP-1 Antibody	Biolegend	329724
APC/Fire™ 810 anti-human CD3	Biolegend	344858
PerCP anti-human CD197 (CCR7)	Biolegend	353242
PerCP/Cyanine5.5 anti-human CD68	Biolegend	333814
Brilliant Violet 570™ anti-human HLA-DR	Biolegend	307638
Spark NIR™ 685 anti-human CD56 (NCAM)	Biolegend	362564
PE/Fire 810 anti-human CD19 Antibody	Biolegend	302287
PE/Cyanine5 anti-human CD15 (SSEA-1)	Biolegend	323014
BUV395 Mouse Anti-Human CD45(HI30)	BD Bioscience	563792
PE-Cy7 Mouse Anti-Human CD25(M-A251)	BD Bioscience	557741
BUV805 Mouse Anti-Human CD8(SK1)	BD Bioscience	612889
BUV615 Mouse Anti-Human CD45RA(HI100)	BD Bioscience	751555
BUV563 Mouse Anti-Human CD45RO(UCHL1)	BD Bioscience	748369
BV421 Mouse Anti-Human CD279 (PD-1)(EH12.1)	BD Bioscience	562516
CD11b RB545 M1/70 100ug	BD Bioscience	569255
Hu CD11c BUV661 B-LY6 25Tst	BD Bioscience	612968
BV786 Mouse Anti-Human CD141(1A4)	BD Bioscience	741006
Hu CD1c RY586 F10/21A3	BD Bioscience	753662
R718 Mouse Anti-Human CD123(9F5)	BD Bioscience	752032
BUV496 Mouse Anti-Human CD117(YB5.B8)	BD Bioscience	750780
BUV737 Mouse Anti-Human CD16(3G8)	BD Bioscience	612786
Recombinant Anti-CD68 Antibody [EPR20545] (ab213363)	Abcam	ab213363

Recombinant Anti-pan Cytokeratin Antibody [AE1/AE3 + 5D3] (ab86734)	Abcam	ab86734
Recombinant Anti-CD8 alpha Antibody [CAL66] (ab237709)	Abcam	ab237709
Recombinant Anti-F4/80 Antibody [SP115] (ab111101)	Abcam	ab111101
Recombinant Anti-CD14antibody [EPR3652] (ab133503)	Abcam	ab133503
Anti-DYKDDDDK Tag (D6W5B) Rabbit mAb	CST	14793S
Protein A/G magnetic beads	Selleck	B23202
Anti-FLAG magnetic beads	Selleck	B26101
Anti-MYC magnetic beads	Selleck	B26301
Anti-HA magnetic beads	Selleck	B26201
HA Tag Recombinant antibody	Proteintech Group	81290-1-RR
MYC tag Polyclonal antibody	Proteintech Group	16286-1-AP
c-SRC Polyclonal antibody	Proteintech Group	11097-1-AP
DyLight® 594 - Lightning-Link® (ab201801)	Abcam	ab201801
Arginase-1 (D4E3M™) XP® Rabbit mAb #93668	CST	#93668
S100A9 (D3U8M) Rabbit mAb #73425	CST	# 73425S
ENSA Polyclonal antibody	Proteintech Group	14518-1-AP
Recombinant Anti-ENSA antibody [EPR8008(2)] (ab180513)	Abcam	ab180513
Phospho-Stat3 (Tyr705) (D3A7) XP® Rabbit mAb #9145	CST	9145S
Recombinant Anti-MCP1 antibody [RM1100] (ab315478)	Abcam	ab315478
Recombinant Anti-MCP1 antibody [EPR27464-89] (ab308522)	Abcam	ab308522
Recombinant Anti-MCP1 antibody [EPR21025] (ab214819)	Abcam	Ab214819
Human MCP-1 ELISA Kit	Proteintech Group	KE00091
Mouse MCP-1 ELISA Kit	Proteintech Group	KE10006
Horseradish peroxidase labeled goat anti-rabbit antibody	Beyotime	A0208
Horseradish peroxidase labeled goat anti-mouse antibody	Beyotime	A0216
Recombinant Anti-Glucose Transporter GLUT1 antibody [EPR3915] (ab115730)	Abcam	AB115730
Recombinant Anti-CREBBP + KAT3B / p300 antibody [EPR23495-276] (ab275379)	Abcam	ab275379
Recombinant Anti-Hexokinase II antibody [EPR20839] (ab209847)	Abcam	ab209847
PPP2CA POLYCLONAL ANTIBODY	Proteintech Group	13482-1-AP

Recombinant Anti-PPP2R2D antibody [EPR13624] (ab181071)	Abcam	ab181071
Recombinant Anti-PKM antibody [EPR10138(B)] (ab150377)	Abcam	ab150377
Recombinant Anti-ENO1 antibody [EPR19758] (ab227978)	Abcam	ab227978
Recombinant Anti-SLC16A3/MCT 4 antibody [EPR28177-30] (ab308528)	Abcam	ab308528
LDHA-Specific Polyclonal antibody	Proteintech Group	19987-1-AP
Anti-L-Lactyl Lysine Rabbit mAb	PTM Bio	PTM-1401RM
Anti-L-Lactyl Lysine Rabbit pAb-BSA and Azide free	PTM Bio	PTM-1425
Anti-ENSA-K63la Rabbit mAb	PTM Bio	customized
Anti-SRC-pS12 Rabbit mAb	PTM Bio	customized
Bacterial and virus strains		
Stbl3	Beyotime	D1081
Biological samples		
Biopsy specimens	This paper	
Paraffin sections	This paper	
Serum	This paper	
Fresh tissue	This paper	
Chemicals, peptides, and recombinant proteins		
Lactate sodium (Synonyms: Lactic acid sodium)	MCE	HY-B2227B
MRTX1133	MCE	HY-134813
2-Deoxy-D-glucose	MCE	HY-13966
PF-4136309 (Synonyms: INCB8761)	MCE	HY-13245
A-485	MCE	HY-107455
STAT3-IN-11	MCE	HY-149007
2-NBDG	MCE	HY-116215
Recombinant Murine M-CSF	Beyotime	P6015
Clodronate Liposomes	Yeasen	40337ES08
Phosphatase inhibitor	Selleck	B15001
Protease inhibitor	Selleck	B14001
RIPA lysis buffer medium	Beyotime	P0013C

Western and IP lysis buffer	Beyotime	P0013
Src Mouse Pre-designed siRNA Set A	MCE	HY-RS13750
InVivoMAb anti-mouse PD-1 (CD279)	Bioxcell	#BE0146
InVivoMAb anti-mouse CD8α	Bioxcell	#BE0061
Toripalimab Injection	TopAlliance	300980001
K63-Peptide-#1 HLYVSPWGG-RLQKGQKYFDSGD	GuoPing Pharmaceutical	Customized
K63-Peptide-#2 HLYVSPWGG-KGQKYFDSGDYNM	GuoPing Pharmaceutical	Customized
K63-Peptide-#3 HLYVSPWGG-LMKRLQKGQKYFD	GuoPing Pharmaceutical	Customized
K63-Peptide-#4 HLYVSPWGG-QKYFDSGDYNMAK	GuoPing Pharmaceutical	Customized
K63-Peptide-#5 HLYVSPWGG-DFLMKRLQKGQKY	GuoPing Pharmaceutical	Customized
K63la-Peptide HLYVSPWGG-LMKRLQKGQK (la) YFD	GuoPing Pharmaceutical	Customized
pS12-Peptide HLYVSPWGG-SKPKDA (p) SQRRSL	GuoPing Pharmaceutical	Customized
Critical commercial assays		
Opal Polaris 7 Color IHC Detection Kits	Akoya	OP-000003
Cellular protein phosphatase 2A (PP2A) active enzyme quantification assay kit	Chundubio	JW50312.1
FastPure Cell/Tissue Total RNA Isolation Kit V2	Vazyme	RC112-01
PrimeScript RT reagent kit	Takara	RR047A
TB Green Premix Ex Taq™ II	Takara	RR820A
Deposited data		
Bulk RNA-seq	TCGA	PAAD
Single-cell RNA-seq	GEO	GSE205013; CRA001160; PHS002371; GSE212966
Experimental models: Cell lines		

KPC	Prof. Raghu Kalluri	
Panc02	Procell	CL-0736
PANC-1	ATCC	CRL-1469
293FT	Thermo	R70007
Experimental models: Organisms/strains		
C57BL/6JGpt	GemPharmatech	N000013
NOD/ShiLtJGpt-Rag1em28Cd94Il2rgem26Cd22/Gpt	GemPharmatech	T002289
huCD34+HSC-NOD/ShiLtJGpt-Prkdcem26Cd52Il2rgem26Cd22/Gpt(CH)	GemPharmatech	T037620
Genetic modified mice KTC (LSL-Kras (G12D/+); Tgfbr2 (flox/flox); p48 (Cre/+))	In-house breeding	
Oligonucleotides		
Ensa-sgrna-f-caccgTGACGAGCGAGGACTTTCTC	Sangon Biotech	customized
Ensa-sgrna-r-aaacGAGAAAGTCCTCGCTCGTCAC	Sangon Biotech	customized
Hk2-shrna-f-ccggGCGGTACAGAGAAAGGAGACTTTTCAAGAGAAAGTCTCCTTTCTCTGTACCGtttttt	Sangon Biotech	customized
Hk2-shrna-r-aattaaaaaaCGGTACAGAGAAAGGAGACTTTCTCTTGAAAAGTCTCCTTTCTCTGTACCGC*	Sangon Biotech	customized
Actb-F-GTGACGTTGACATCCGTAAAGA	Sangon Biotech	customized
Actb-R-GCCGGACTCATCGTACTCC	Sangon Biotech	customized
Ccl2-F-TAAAAACCTGGATCGGAACCAAA	Sangon Biotech	customized
Ccl2-R-GCATTAGCTTCAGATTTACGGGT	Sangon Biotech	customized
Arg1-F-CTCCAAGCCAAAGTCCTTAGAG	Sangon Biotech	customized
Arg1-R-GGAGCTGTCATTAGGGACATCA	Sangon Biotech	customized
S100a9-F-GCACAGTTGGCAACCTTTATG	Sangon Biotech	customized
S100a9-R-GCACAGTTGGCAACCTTTATG	Sangon Biotech	customized
Il10-F-GCACAGTTGGCAACCTTTATG	Sangon Biotech	customized
Il10-R-GCAGCTCTAGGAGCATGTGG	Sangon Biotech	customized
ACTB-F-CATGTACGTTGCTATCCAGGC	Sangon Biotech	customized
ACTB-R-CTCCTTAATGTCACGCACGAT	Sangon Biotech	customized

CCL2-F- CAGCCAGATGCAATCAATGCC	Sangon Biotech	customized
CCL2-R-TGGAATCCTGAACCCACTTCT	Sangon Biotech	customized
Recombinant DNA		
pSLenti-EF1-EGFP-P2A-Puro-CMV-MCS-3xFLAG-WPRE	Obio	GL107
pSLenti-EF1-EGFP-P2A-Puro-CMV-Ensa (WT)-3xFLAG-WPRE	Sangon Biotech	customized
pSLenti-EF1-EGFP-P2A-Puro-CMV-Ensa (K40R)-3xFLAG-WPRE	Sangon Biotech	customized
pSLenti-EF1-EGFP-P2A-Puro-CMV-Ensa (K40R)-3xFLAG-WPRE	Sangon Biotech	customized
pSLenti-EF1-EGFP-P2A-Puro-CMV-Ensa (K56R)-3xFLAG-WPRE	Sangon Biotech	customized
pSLenti-EF1-EGFP-P2A-Puro-CMV-Ensa (K56R)-3xFLAG-WPRE	Sangon Biotech	customized
pSLenti-EF1-EGFP-P2A-Puro-CMV-Ensa (K74R)-3xFLAG-WPRE	Sangon Biotech	customized
pSLenti-EF1-EGFP-P2A-Puro-CMV-Ensa (K80R)-3xFLAG-WPRE	Sangon Biotech	customized
pSLenti-EF1-EGFP-P2A-Puro-CMV-Cks1b-3xFLAG-WPRE	Sangon Biotech	customized
pSLenti-EF1-EGFP-P2A-Puro-CMV-Nucks-3xFLAG-WPRE	Sangon Biotech	customized
pSLenti-EF1-EGFP-P2A-Puro-CMV-Nucks-3xFLAG-WPRE	Sangon Biotech	customized
pSLenti-EF1-EGFP-P2A-Puro-CMV-SRC (WT)-2*MYC-WPRE	Sangon Biotech	customized
pSLenti-EF1-EGFP-P2A-Puro-CMV-Src (S12A)-2*MYC-WPRE	Sangon Biotech	customized
pSLenti-EF1-EGFP-P2A-Puro-CMV-Src (S12D)-2*MYC-WPRE	Sangon Biotech	customized
pSLenti-EF1-EGFP-P2A-Puro-CMV-HA-Ppp2ca-WPRE	Sangon Biotech	customized
pSLenti-EF1-EGFP-P2A-Puro-CMV-HA-Ppp2r2d-WPRE	Sangon Biotech	customized
LentiCRISPRV2-GFP	Shhebio	P2262
pLKO.1-EGFP	Shhebio	P3084
Software and algorithms		

Inform 2.5.0	Akoya	https://www.akoyabio.com/phenoimager/inform-tissue-finder/
Flowjo 10.8.1	Becton Dickinson	https://www.flowjo.com/
GraphPad Prism 7.0	GraphPad Inc	https://www.graphpad.com/

Supplementary Table 6 Protein interacted with PPP2CA

Accession	Sum PEP Score	Score Sequest HT: Sequest HT	# Peptides
Q92499	303.78	485.02	38
P07900	299.374	414.02	43
P08238	293.347	424.81	43
P11142	283.239	349.91	30
P10809	275.648	384.67	32
P0DMV9	266.34	356.59	27
O00571	252.659	325.48	31
P35908	246.694	297.98	30
P04264	243.735	304.34	28
Q00610	233.999	222.2	37
P13639	230.852	287.58	34
P07437	227.559	313.67	22
P06733	225.418	228.7	27
P14618	220.977	294.18	26
Q08211	215.482	224.51	36
P14625	213.99	223.55	34
P12956	211.752	172.98	30
P49327	206.707	171.81	42
P09874	206.273	157.99	33
P13010	203.451	168.78	22
Q9Y3I0	203.126	257.05	23
CON_P02769	202.542	240.86	30
Q13283	199.093	282.94	18
P68104	195.966	233.78	12
P11021	195.767	298.84	25
P11940	190.505	232.01	23
P68371	189.581	278.82	18
P06744	189.455	131.63	20
Q12906	188.674	163.66	24
P60709	187.81	258.29	20
Q14697	186.723	142.95	27
CON_P13645	186.366	296.91	27
P42704	184.416	111.7	38
P78371	179.648	168.44	23
P35579	175.07	122.3	32
P06576	173.829	167.47	18
P04406	173.212	219.08	16
Q9NQX3	170.864	158.39	24
P35580	169.426	126.77	31
P48643	167.676	114.09	25
Q99832	166.934	153.25	22
P12277	165.943	164.08	16
Q9BVA1	164.876	245.42	16
P11586	164.461	156.1	25
Q13885	163.91	244.6	16

P29401	159.892	113.49	19
P04350	159.47	254.61	16
Q13310	158.747	176.85	20
Q92841	156.636	218.06	28
Q14738	156.182	140.57	25
P07814	154.101	104.9	31
P34932	153.917	112.91	23
Q71U36	150.63	181.47	20
P07195	148.469	124	16
P50395	148.4	128.37	23
Q9UN86	146.448	122.64	15
Q7L2E3	145.366	127.72	26
P07237	143.816	132.99	22
O43707	142.721	111.58	25
P25705	142.509	140.09	20
P17844	142.372	173.46	25
P13797	141.7	136.93	22
P49368	141.599	170	19
P60842	141.068	170.92	22
Q9HCE1	137.949	93.22	23
P31939	137.259	151.24	23
P55072	136.086	109.21	22
Q05639	136.024	179.52	13
P09651	135.634	162.01	17
Q9BQE3	134.709	173.38	20
O75533	133.396	97.22	24
P67775	133.023	144.1	13
O60506	130.805	130.12	23
O43143	129.854	129.36	22
Q6PKG0	129.076	96.41	25
Q00839	126.37	122.81	18
P18206	126.134	104.95	27
P30153	125.303	168.5	20
P19338	124.532	193.88	20
P49588	123.986	114.26	21
P51659	122.91	111.17	18
CON_P35527	122.191	92.91	17
Q92900	121.868	99.64	25
P49591	121.305	100.54	18
P50990	120.295	153.55	22
Q14444	119.462	112.55	14
Q13813	119.225	71.51	22
P12814	117.259	110.02	20
Q7KZF4	117.046	102.75	20
P54136	116.961	105.37	25
P00558	116.875	127.96	18
P52272	116.826	126.33	18
[illegible]	[illegible]	[illegible]	[illegible]

P39023	116.417	156.01	11
P13667	116.413	146.38	22
P36578	115.176	127.88	17
P38646	115.171	127.81	18
P41252	115.121	87.4	26
P00338	115.029	123.1	20
P18669	114.948	99.78	11
P26641	114.887	101.68	15
Q92945	114.064	112.09	16
P68366	114.031	141.01	14
Q9Y5B9	113.911	86.28	20
P54886	113.221	86.11	13
O43390	113.197	107.87	23
Q9NYF8	112.1	69.99	16
P30101	111.62	90.55	21
P17987	111.486	107.95	17
Q9NR30	111.365	83.96	23
Q15393	111.355	84.39	20
P08758	110.328	99.42	20
Q9H0D6	110.236	93.62	20
Q16531	109.841	88.18	22
Q15084	109.794	104.19	12
P04075	109.062	91.36	14
P05455	108.974	81.52	19
P22626	107.933	166.58	16
Q9Y4L1	107.093	81.22	18
P49321	106.963	63.8	13
P50991	105.891	95.09	20
P23246	105.773	99.88	13
P08133	105.062	91.25	22
P26599	104.527	123.02	12
P41250	104.392	89.82	16
P12268	104.366	129.44	15
Q9BQG0	103.815	74.38	22
Q9Y262	103.598	73.95	17
Q12905	103.518	160.61	12
Q02790	102.848	88.66	19
Q9Y265	102.34	108.87	17
Q15029	102.033	120.09	18
P31943	101.102	118.37	13
P40926	100.63	111.72	14
P40227	100.609	96.38	16
O95202	100.471	67.68	15
Q9Y2W1	100.162	108.17	16
P23526	99.664	84.88	13
Q9Y490	99.533	61.67	18
P23396	99.118	140.85	13
[illegible]	[illegible]	[illegible]	[illegible]

P49411	97.555	80.64	13
P55786	97.497	92.16	18
P23528	97.423	73.89	13
P06748	97.069	86.54	8
O00231	97.022	90.28	18
O43242	96.768	75.98	18
P08865	96.583	93.3	14
Q9UMS4	96.139	77.91	12
Q9BUF5	96.001	149.47	13
Q13509	94.931	159.34	12
Q86VP6	94.069	85.21	22
P54577	93.805	105.85	20
P26639	93.647	86.52	19
Q02878	93.52	80.49	17
P16615	93.433	60.88	18
Q04637	92.87	71.35	20
P09960	92.552	79.63	17
P31948	92.497	101.47	18
Q9P2E9	92.398	60.35	17
P55060	91.795	69.11	17
P31150	91.633	74.96	17
P04843	91.309	72.38	14
P13647	90.993	103.3	19
P63244	90.369	83.8	14
Q6P2Q9	90.058	44.86	24
P60174	89.963	119.57	12
P27824	89.861	85.51	12
Q9P2J5	89.768	64.57	19
P31942	89.644	63.9	12
P62258	89.345	115.54	14
P62263	89.268	83.02	9
P34931	88.893	143.66	9
Q7Z417	88.759	72.35	13
P33993	88.675	62.64	15
P00918	88.359	72.39	12
P46777	87.691	72.58	11
Q13200	86.785	61.38	16
O43175	86.709	116.22	13
P62424	86.547	85.21	14
P56192	86.517	50.35	14
P62937	86.278	78.59	11
P27797	84.985	77.51	13
Q8WWM7	84.686	72.87	16
P23381	84.378	83.45	14
Q9NVP1	84.201	63	15
P62906	84.023	97.94	12
Q14974	83.544	71.7	15
[illegible]	[illegible]	[illegible]	[illegible]

P51610	83.251	47.36	16
P08670	83.191	86.18	15
P51991	83.105	76.23	9
Q5JSZ5	83.094	42.34	17
Q9BQ67	82.97	55.95	12
P38919	82.586	67.06	14
Q14240	82.584	92.95	15
P46940	82.532	44.67	18
O14980	82.175	80.01	15
P49915	81.511	59.41	17
Q15637	81.232	78.1	9
Q9P258	80.841	66.15	16
P55884	80.739	70.76	15
Q15459	80.708	56.76	14
P08195	80.422	73.17	14
P04844	80.392	57.94	11
P62195	80.217	83.94	13
P62701	80.195	81.86	14
P51665	80.12	47.75	7
P51114	79.882	64.42	12
Q9Y230	79.587	86.63	14
P27816	79.537	86.09	13
P43686	79.533	43.68	13
P53621	79.429	56.14	17
P62136	79.34	75.13	12
P26196	79.309	58.08	17
P02786	79.284	86.46	15
O75643	79.116	55.91	21
P06737	79.04	52.96	19
Q02880	78.942	45.84	16
P27695	78.365	49.24	11
Q14152	78.164	61.61	18
P05023	77.795	73.89	14
P30041	77.753	96.67	12
P61978	77.394	68.7	14
Q9NSE4	77.3	51.67	19
Q99613	76.906	63.7	16
Q9NTK5	76.882	56.48	15
P05198	76.763	60.19	15
P19474	76.462	68.78	15
O95793	76.453	41.06	14
Q13263	76.419	59.23	12
Q07065	76.337	55.27	15
Q9Y617	76.313	88.6	15
A6NNZ2	76.274	129.91	8
P08243	76.208	81.29	17
P07954	76.128	53.64	10
[illegible]	[illegible]	[illegible]	[illegible]

P54819	76.019	51.75	8
P36873	75.332	60.97	11
P51116	74.316	53.27	12
Q96QK1	74.267	42.62	15
P63104	74.228	112.52	11
Q13435	73.99	58.32	14
Q9Y266	73.98	43.12	15
Q09028	73.711	43.72	10
Q16658	73.677	54.02	13
Q16576	73.336	51.14	11
Q96AE4	73.306	63.14	15
Q8WUM4	73.279	54.65	15
P42285	72.919	35.03	16
P61221	72.799	56.47	12
P35232	72.374	80.41	10
O15226	72.309	54.76	17
O60271	72.229	41.21	15
Q9NZI8	72.147	76.34	13
O95831	72.075	70.65	12
Q9Y224	72.04	77.26	11
P61163	72.032	45.16	10
P54652	71.986	96.86	10
Q15366	71.882	99.92	8
P53618	71.876	37.43	12
P26038	71.865	67.51	16
Q9Y3F4	71.473	46.65	9
Q1KMD3	71.471	55.74	14
Q9BUJ2	71.272	51.14	10
P17812	71.216	58.16	12
Q15424	70.899	39.88	9
P52597	70.899	71.93	8
P11388	69.8	47.66	18
P09211	69.468	71.28	8
P62140	69.448	60.09	10
P62917	69.057	84.21	11
P25787	68.613	58.19	7
Q06830	68.399	65.66	11
P41091	68.156	47.02	12
O00567	68.091	43.51	13
O00410	67.959	51.54	13
P08708	67.876	83.9	10
P50454	67.64	43.98	8
Q9UHB9	67.585	44.36	14
P28838	67.579	51.9	13
Q14103	67.487	67.75	9
Q9UNM6	67.454	32.13	12
P26640	67.24	37.08	12
[illegible]	[illegible]	[illegible]	[illegible]

P53396	66.797	50.63	14
P12004	66.582	54.92	8
O95757	66.196	61.17	11
P30837	66.178	44.4	10
Q08945	66.017	51.72	13
P22087	65.89	61.06	10
P55084	65.857	56.83	14
P17174	65.634	78.09	10
Q16836	65.565	42.78	8
P09972	65.545	63.81	8
Q9UJV9	65.434	53.93	12
P78527	65.314	27.77	18
Q92804	65.303	47.62	8
P15170	65.261	63.34	10
CON_ENSEMBL:ENSBTAP00000031900	65.188	39.43	10
O00425	65.132	63.56	12
P11387	64.605	52.83	15
P22102	64.451	40.7	14
P46459	64.42	52.79	15
Q01082	64.372	29.61	16
Q9UQE7	64.238	28.3	16
P50570	64.1	29.45	17
P35241	63.838	68	17
Q9NTJ3	63.732	40.7	16
P00367	63.533	48.61	12
P20290	63.522	44.26	6
P31689	63.477	56.52	9
P12235	63.307	48.7	12
P62191	63.298	73.55	11
Q9NVI7	63.214	52.96	13
Q14166	63.101	50.18	10
P31153	62.886	48.25	11
P27708	62.805	30.48	13
O00232	62.694	39.23	12
Q96SB4	62.693	59.22	9
Q9BZZ5	62.63	38.51	12
P00505	62.4	111.56	12
P36871	62.395	35.45	13
Q8TEX9	62.353	37.61	14
P46781	62.336	78.66	16
P52209	62.261	53.31	12
Q2TAY7	62.181	31.22	11
Q13347	62.155	49.73	10
O15027	62.051	42.87	13
P22234	61.834	73.92	10
Q5T9A4	61.797	60.26	11
Q99729	61.678	82.36	11
[illegible]	[illegible]	[illegible]	[illegible]

P15311	61.301	65.11	15
Q9NUU7	61.279	38.21	9
Q14694	61.198	52.45	9
Q96PK6	61.037	55.02	13
P21796	60.988	51.09	10
Q14683	60.776	42.31	15
Q9BXJ9	60.64	35.67	13
P00491	60.517	74.29	9
Q9UQ35	60.419	47.82	12
Q9BVP2	60.36	52.45	12
P27348	60.076	70.76	9
P43243	59.999	40.97	12
Q5JSH3	59.987	33.03	12
P37802	59.909	63.98	8
P67809	59.637	42.45	9
P02545	59.625	30.66	12
P21333	59.529	29.74	12
Q13620	59.455	35.24	13
O14818	59.453	58.39	11
P49736	59.322	39.65	11
Q96A49	59.071	37.99	9
Q9NYU2	58.918	33.07	11
P62277	58.887	53.85	10
P25205	58.784	36.14	11
P30050	58.723	69.08	7
P30154	58.462	43.56	8
Q06210	58.319	34.59	12
O75874	58.299	25.75	11
Q15008	58.186	49.23	13
P61204	58.132	51.83	8
O95782	57.772	45.55	10
O00151	57.547	36.09	6
P35998	57.394	59.03	13
P42166	57.252	40.94	11
Q99623	57.078	51.49	10
P42025	56.93	40.37	8
P43304	56.704	37.37	10
P12081	56.666	57.05	11
P48634	56.524	38.01	9
Q8N1F7	56.52	36.4	12
Q15046	56.215	48.56	11
Q9Y295	56.197	32.46	11
P16989	56.141	50.67	8
P35637	56.123	69.67	7
P06493	56.075	42.48	11
Q13362	56	49.67	11
O95347	55.947	29.3	13
[illegible]	[illegible]	[illegible]	[illegible]

P49916	55.848	37.62	12
O60763	55.813	32.76	11
P62249	55.789	94.58	11
O14654	55.751	32.38	15
P60228	55.623	54.55	10
Q96CS3	55.614	36.11	8
O75534	55.488	36.2	12
O75319	55.487	32.37	11
P46087	55.342	46.36	12
P46060	55.337	35.09	13
Q9BQ52	55.333	39.39	9
P18124	55.309	61.02	13
O43172	55.201	26.91	9
P33176	55.185	54.04	11
O00429	55.148	42.55	12
P14866	55.142	43.66	8
Q01813	55.083	34.42	13
P68032	54.871	116.08	9
P15121	54.409	32.83	7
Q13838	54.358	57.66	11
P27635	54.329	64.49	10
P07355	54.27	39.76	10
Q9BUQ8	54.065	34.85	14
O94973	53.592	40.55	9
O43615	53.566	37.88	10
P63151	53.55	38.38	7
CON_Q0IIK2	53.507	39.8	11
P50995	53.45	37.04	12
P15880	53.398	72.68	10
Q96I24	53.391	39.97	11
O00303	53.218	49.18	9
Q13765	53.134	43.67	5
O76021	52.988	41.45	10
O75083	52.919	27.95	7
Q02978	52.813	39.76	8
P15924	52.701	26.86	12
Q96PZ0	52.684	20.35	10
P55209	52.66	40.42	8
Q14008	52.517	28.7	12
P49755	52.456	35.95	5
Q07955	52.453	85.03	10
P62826	52.376	65.59	6
P51398	52.049	29.26	10
P04259	52.046	73.55	11
P08237	52.029	34.93	11
P46783	51.847	94.29	7
P78347	51.749	27.74	15
[illegible]	[illegible]	[illegible]	[illegible]

P19367	51.676	30.69	13
O15371	51.574	46.83	10
O60832	51.495	49.68	10
Q92973	51.302	39.77	9
P02533	51.251	68.81	13
Q9H0A0	51.093	27.98	15
Q8NBS9	51.083	36.78	9
P34897	51.079	52.88	11
Q8IY81	51.062	26.58	9
Q9P035	50.945	36.12	7
Q6P2E9	50.928	25.65	9
Q9H6S0	50.845	35.88	12
Q99873	50.828	42.41	7
P84085	50.786	45.06	7
P04040	50.735	24.42	9
Q15813	50.722	30.56	8
Q9UQ80	50.639	62.86	10
Q15233	50.241	73.75	10
Q15181	50.209	49.15	7
Q7L014	50.192	35.88	12
O76094	50.166	31.91	8
P00387	50.121	50.07	7
Q9H9A6	50.118	28.44	12
P48047	50.022	34.3	8
Q12931	50.002	64.31	10
P52292	49.994	46.77	9
P33991	49.934	25.13	10
Q01581	49.748	28.55	9
P23284	49.635	57.72	9
P37837	49.591	35.56	12
Q96AG4	49.422	47.1	8
Q8N5C6	49.402	33.18	13
P30566	49.288	30.55	7
Q9UHX1	49.202	43.1	10
P15531	49.197	38.95	9
O43776	49.185	48.55	11
P10412	48.972	82.71	7
P17980	48.962	34.8	10
O00541	48.903	24.78	11
P13995	48.884	49.91	5
O43823	48.88	40.41	11
P16152	48.826	61.1	7
P07910	48.766	44.69	6
Q06203	48.747	41.13	10
P16435	48.726	42.51	10
O95429	48.707	38.98	9
Q9BZE4	48.624	47.99	11
[illegible]	[illegible]	[illegible]	[illegible]

Q9BTV4	48.326	36.44	7
Q15019	48.282	33.33	8
Q7L2H7	48.254	31.79	9
Q13162	48.252	45.51	7
Q10567	48.172	25.29	8
O75131	48.075	29.26	11
P52907	47.934	23.14	6
Q86UP2	47.901	20.39	8
Q86XP3	47.845	36.34	10
Q8N684	47.771	31.4	8
Q9NY33	47.723	30.89	9
P28074	47.697	29.28	7
Q96SI9	47.633	37.79	9
P20839	47.562	30.74	7
P39656	47.519	59.96	7
Q9H4A4	47.48	41.32	11
Q16555	47.412	38.48	9
CON_P34955	47.288	36.36	8
Q01518	47.249	40.99	10
P0DN79	47.08	32.81	9
P35606	47.035	31.16	10
Q9UNQ2	46.916	47.43	9
P32969	46.898	50.09	7
Q9Y285	46.726	32.42	9
P68400	46.679	37.37	7
Q9NZB2	46.465	25.72	11
P62753	46.433	61.05	7
P23921	46.379	39.47	8
O60264	46.354	33.34	13
Q06787	46.334	34.21	12
P35249	46.181	34.09	9
P08559	45.972	38.21	9
P62241	45.946	58.4	9
Q9GZL7	45.869	34.1	7
P05141	45.743	62.21	11
O60749	45.738	41.07	11
P05388	45.73	84.88	10
P51148	45.654	51.73	7
Q15365	45.544	34.25	6
P51812	45.453	30.3	9
P17858	45.435	29.45	9
Q14690	45.396	21.59	14
Q13616	45.358	30.4	12
P35573	45.281	19.77	12
O94788	45.159	41.1	8
P62750	45.021	57.76	8
P48444	45.009	55.24	9
[illegible]	[illegible]	[illegible]	[illegible]

O15067	44.872	24.25	9
Q14203	44.821	26.74	7
P62333	44.77	26.76	7
Q14157	44.667	33.41	7
Q9HB71	44.514	27	7
Q13155	44.465	36.04	7
P40429	44.425	68.53	9
Q969V3	44.414	23.9	8
Q14C86	44.31	27.7	10
P31040	44.25	39.84	7
P61106	44.185	42.17	8
P78344	44.177	31.73	12
O14776	44.154	29.62	15
O00299	44.04	41.3	8
Q14839	44.005	17.83	10
P36957	43.879	46.17	6
P62081	43.868	79.46	7
P08397	43.843	19.77	5
O95373	43.733	28.38	9
Q9Y277	43.725	30.66	7
P48637	43.7	27.79	11
O95433	43.685	23.66	9
P30519	43.681	31.06	6
P00492	43.668	32.92	7
Q14684	43.648	34.91	9
P00441	43.611	33.51	4
P32119	43.611	28.12	6
Q14498	43.591	48.32	7
P09661	43.232	39.91	9
P55795	43.195	34.34	8
Q16891	43.14	17.76	7
P49257	43.124	26.2	5
Q96DI7	43.095	41.35	7
Q8WXX5	43.076	20.62	7
P09012	42.865	22.25	4
Q13610	42.832	43.39	7
P46782	42.732	34.9	7
Q9Y383	42.661	26.81	7
P82650	42.546	22.53	8
O95861	42.5	31.26	9
Q9Y2R4	42.492	27.7	7
Q96GQ7	42.406	30.91	11
Q969Z0	42.392	23.11	7
P45880	42.377	53.88	8
P00390	42.306	34.35	8
O14979	42.291	50.07	7
O00148	42.274	49.98	9
[illegible]	[illegible]	[illegible]	[illegible]

Q8NE71	42.031	30.44	9
Q92598	41.982	30.38	7
Q15436	41.949	27.51	6
P28066	41.93	42.19	7
P07737	41.915	49.46	8
P21399	41.89	19.87	10
P62244	41.88	55.66	6
P18085	41.841	34.99	6
Q9BWD1	41.807	21.85	5
Q9H2Y7	41.685	28.99	7
Q9UHD1	41.653	26.24	4
Q9NUL7	41.64	24.52	7
O94905	41.536	32.13	7
O43148	41.493	25.77	7
Q52LJ0	41.48	22.53	8
P09936	41.376	21.23	7
Q15021	41.348	21.86	10
CON_ENSEMBL:ENSBTAP00000024146	41.309	20.96	10
Q9HDC9	41.247	52.36	8
Q07021	41.089	47.44	5
P63241	41.076	40.63	4
Q13895	41.039	23.94	9
Q93009	41.039	16.2	14
P49589	41.035	35.37	11
P10515	41.011	26.79	8
P34949	40.921	20.71	6
Q8TCS8	40.858	25.87	11
P26368	40.856	36.68	6
Q96HE7	40.825	19.36	9
Q96EY1	40.725	22.43	7
Q16850	40.673	24.56	8
Q14137	40.466	34.47	7
Q02543	40.4	40.18	9
P11216	40.393	29.8	10
O76003	40.244	37.62	9
Q06323	40.237	41.13	7
Q92688	40.195	50.65	6
Q99798	40.193	29.21	10
O00116	40.155	24.73	8
Q9Y2S7	40.141	27.97	8
Q9Y2L1	40.137	32.41	9
O00154	40.13	39.65	6
Q9NSD9	40.082	32.87	9
P51149	40.041	32.97	7
P78318	40.005	25.15	7
Q12972	40.005	24.87	5
Q00341	39.856	22.55	11
[illegible]	[illegible]	[illegible]	[illegible]

Q04837	39.8	40.14	8
O15042	39.745	22.27	8
Q15691	39.62	25.4	6
Q92616	39.584	14.88	12
Q9H6R4	39.583	15.43	5
P29373	39.538	32.53	6
P61247	39.527	53.38	9
P67936	39.46	22.8	8
Q9Y678	39.337	36.95	9
P11177	39.329	30.31	8
P61158	39.321	21.73	8
Q99460	39.293	29.56	8
P36776	39.267	15.43	8
Q8TDD1	39.19	24.53	10
Q9UBE0	39.163	21.67	6
Q8N766	39.159	20.36	7
Q9H0S4	39.087	39.09	8
Q14566	39.038	27.38	10
Q9Y2Z0	39.035	24.06	7
P14314	38.968	38.48	7
Q01780	38.919	25.25	8
O14929	38.889	21.46	7
P19784	38.874	22.15	8
Q9BWF3	38.747	31.25	6
Q99829	38.731	33.5	8
Q9Y6M1	38.688	41.45	9
P30084	38.633	24.21	7
P04181	38.624	35.95	8
P62805	38.47	58.09	5
O14744	38.427	34.73	8
O43852	38.355	31.53	5
Q14247	38.332	29.59	7
P04818	38.254	30.43	6
P22695	38.24	29.37	7
P54578	38.187	32.08	8
P45974	38.176	23.49	9
Q9H5H4	38.153	23.31	8
P06132	38.136	18.54	6
Q9NVA2	38.089	38.98	9
P08779	38.066	41	11
Q12874	38.033	21.68	9
Q13573	37.779	23.91	7
P40925	37.653	37.08	6
P47897	37.638	32.81	10
P43490	37.609	27.66	9
Q9UNZ2	37.583	32.25	6
P49419	37.511	17.25	9
[illegible]	[illegible]	[illegible]	[illegible]

Q13618	37.23	20.67	7
P62888	37.199	37.12	4
Q15645	37.19	26.44	8
P28331	37.135	26.94	8
Q9BQ39	37.124	33	9
Q99714	37.096	48.33	6
Q99497	37.061	43.55	6
CON_P02535-1	36.964	98.17	9
Q08J23	36.91	36.27	8
P11310	36.821	33.79	8
Q9Y450	36.778	22.89	8
Q9UKD2	36.694	23.95	6
Q04760	36.69	44.57	6
O96008	36.655	27.26	6
P09104	36.548	37.56	5
P36551	36.472	34.56	7
Q9UHI6	36.396	28.42	9
P39019	36.385	51.86	7
Q9BQ04	36.384	24.07	6
P17028	36.344	26.38	7
P38606	36.277	19.6	10
Q7Z2W4	36.255	26	7
Q53H12	36.207	26.59	5
P51572	36.171	28.21	6
Q8IXT5	36.085	16.52	10
Q09161	35.982	27.59	10
P41227	35.953	23.36	5
P57088	35.935	28.04	8
Q07666	35.859	28.16	6
P60900	35.654	49.7	6
Q9H6Z4	35.645	29.38	7
Q9NZL9	35.594	27.6	6
P09622	35.583	24.54	5
Q10713	35.579	17.36	6
P46778	35.493	30.67	4
Q16543	35.493	20.8	7
Q14651	35.326	32.75	7
P61313	35.293	44.86	6
P09429	35.258	42.89	4
P31946	35.194	44.96	6
P63010	35.187	20.76	8
P61086	35.123	17.47	6
Q02241	35.115	16.52	6
O14828	35.046	22.7	5
P21281	35.004	18.81	7
P43034	34.988	16.86	9
Q9H2U2	34.953	31.4	7
[illegible]	[illegible]	[illegible]	[illegible]

O15355	34.899	27.82	7
Q9Y696	34.874	11.2	5
Q9NNW5	34.868	16.88	9
Q6PI48	34.792	23.81	9
Q8N1G4	34.789	26.57	9
Q96CX2	34.771	37.35	7
P61019	34.713	47.05	5
P30043	34.7	17.32	4
P07099	34.658	21.27	10
P32929	34.592	25.33	7
Q16881	34.53	19.72	5
P62841	34.481	29.28	3
Q12996	34.456	16.95	7
Q9NX58	34.339	20.56	5
O75390	34.299	21.73	7
Q8N0Y2	34.249	21.12	5
Q13724	34.243	22.35	9
O43795	34.209	19.14	8
P62495	34.204	45.21	7
Q9UBT2	34.151	28.39	7
Q9H307	34.119	21.15	10
Q00688	34.107	23.38	6
Q16181	34.099	26.94	4
Q9GZR7	34.098	16.88	5
P30520	34.089	33.32	7
Q9UJZ1	34.027	22.61	5
Q01105	33.977	28.83	6
Q99733	33.844	26.4	6
Q9BXP5	33.802	26.94	8
P62269	33.705	49.15	10
P53985	33.651	19.16	5
O94874	33.631	16.97	8
P32322	33.555	32.78	6
Q9HCC0	33.476	17.69	7
P49590	33.391	25.41	9
P08621	33.381	22.62	5
O75153	33.249	17.22	9
P23258	33.223	15.04	6
Q9NQ29	33.164	22.46	6
P04083	33.152	19.81	6
Q15293	33.151	13.86	7
P68036	33.122	30.21	6
P43246	33.098	13.08	8
P20042	33.046	21.5	7
P61619	32.937	25.8	5
Q8NC51	32.925	39.35	8
P13489	32.879	18.69	6
[illegible]	[illegible]	[illegible]	[illegible]

Q5JTH9	32.712	12.1	10
Q15717	32.567	37.75	6
P42167	32.564	30.07	7
O14647	32.562	12.71	12
P55036	32.561	16.15	4
Q9BSD7	32.499	19.17	5
P35268	32.489	38.47	4
P10768	32.445	31.37	4
P30044	32.425	22.47	7
P31930	32.42	9.74	7
P15927	32.365	25.43	5
Q01844	32.254	17.75	5
O75396	32.25	22.01	6
O75489	32.214	26.95	7
Q9UBQ7	32.175	27.52	4
Q5JTZ9	32.141	27.7	9
P24752	32.11	33.78	7
Q99615	32.077	18.98	7
Q9UMX0	31.962	19.76	5
P38117	31.924	33.1	7
P05091	31.911	20.39	8
P47755	31.89	21.91	4
P62829	31.882	27.3	3
Q9BY44	31.863	23.16	7
Q14677	31.805	18.36	6
Q96G03	31.795	16.98	6
Q9UKV8	31.789	23.13	7
Q92769	31.784	20.43	6
P61224	31.694	31.5	6
P28370	31.636	23.13	9
P19623	31.621	29.31	5
P53041	31.608	19.71	7
P38432	31.598	28.31	5
P30419	31.541	31.28	4
Q86V81	31.464	27.9	4
O94906	31.452	17.59	9
P61011	31.384	19.86	7
Q05519	31.321	14.35	4
Q13011	31.277	35.26	5
O95336	31.239	30.84	7
Q08170	31.208	24.7	6
O95747	31.182	11.83	6
Q14320	31.158	19.94	5
P49720	30.968	32.84	5
Q8IUF8	30.951	18.47	5
Q9Y2X3	30.936	20.69	6
Q5BKZ1	30.893	31.56	7
[illegible]	[illegible]	[illegible]	[illegible]

Q9UKV3	30.584	16.43	10
Q13098	30.584	27.1	5
Q92552	30.583	22.95	7
P30876	30.47	12.88	7
Q04323	30.419	15.91	5
Q9H9T3	30.405	18.23	9
Q9BW92	30.388	28.13	7
P51003	30.293	24.36	7
P61981	30.29	36.68	6
Q6UB35	30.232	19.09	9
O15372	30.215	25.86	7
Q9Y3Z3	30.169	18.62	9
O76031	30.169	13.03	7
Q99848	30.144	23.98	5
P20618	30.077	24.23	5
P49721	30.076	28.09	5
P37108	30.06	22.92	5
Q07960	30.051	17.06	5
P0CG39	30.049	80.85	4
Q08257	30.044	27.13	4
Q13177	30.012	15.36	8
P61289	29.958	20.37	5
Q8NI27	29.905	16.76	7
Q6P158	29.893	29.27	8
Q5TFE4	29.887	11.49	8
Q10471	29.741	12.02	7
Q92734	29.717	21.68	3
Q96P16	29.646	15.52	5
P62314	29.61	37.99	3
P14923	29.599	12.49	5
P25788	29.579	38.42	5
P53999	29.578	38.33	5
P24539	29.565	21.74	5
Q9H078	29.499	14.61	7
P20700	29.45	24.02	7
Q9Y547	29.433	17.11	3
Q15435	29.335	11.37	8
O15347	29.293	18.93	4
P11172	29.292	35.97	6
O43791	29.278	23.39	4
P42126	29.273	25.89	7
Q9NW13	29.262	28.95	8
P55010	29.174	17.98	8
P43487	29.171	31.54	4
Q06124	29.157	24.39	5
P62910	29.103	25.84	5
P61026	29.087	47.25	5
[illegible]	[illegible]	[illegible]	[illegible]

Q12788	29.032	23.58	5
O00203	28.981	20.94	9
Q9H2U1	28.949	13.7	6
P46776	28.926	34.04	4
Q00325	28.922	55.72	7
Q13151	28.882	34.06	5
P49189	28.88	34.11	7
P56182	28.728	12.31	4
Q9UBX3	28.701	20.92	6
Q8TC12	28.666	21.91	5
P12955	28.655	24.08	7
P38159	28.64	27.41	6
P39748	28.613	27.61	6
P22059	28.571	23.68	4
O75663	28.529	21.46	8
O75347	28.528	24.09	4
Q96I99	28.499	16.82	7
O15160	28.473	20.41	6
P11908	28.401	17.13	6
O15144	28.391	21.83	7
Q16537	28.344	19.23	6
Q15006	28.333	29.09	5
O60256	28.201	21.4	7
O00264	28.191	27.21	5
P22061	28.183	10.66	6
P07384	28.167	11.8	8
CON_P08730-1	28.165	51.51	8
Q13596	28.162	17.7	8
Q96EY7	28.134	14.14	6
Q9UFN0	28.095	14.37	5
O95394	28.091	16.78	8
Q9Y606	28.084	18.82	5
P18621	28.081	33.95	5
Q53GS9	28.023	10.22	8
P07741	28.02	25.13	5
Q13243	27.992	31.86	5
Q9NW64	27.941	21.13	7
P46109	27.895	17.98	7
Q92979	27.852	10.27	6
O75436	27.85	16.65	6
P06280	27.825	21.26	6
Q99755	27.795	14.7	4
Q15287	27.788	23	4
P28070	27.771	12.44	4
A5YKK6	27.745	13.02	8
P60510	27.692	24.31	4
Q9Y2Z4	27.649	19.86	6
[illegible]	[illegible]	[illegible]	[illegible]

Q16630	27.623	14.11	4
P36507	27.586	7.37	6
Q13247	27.507	25.85	7
Q8N1N4	27.485	16.58	8
CON_P00761 SWISS-PROT:P00761	27.474	223.04	4
Q8NB90	27.46	33.49	6
P25786	27.436	15.45	5
Q96CN7	27.426	24.39	5
Q9NTJ5	27.418	21.82	8
P55265	27.41	20.53	7
P35251	27.364	21.32	7
Q9NQR4	27.347	16.82	5
P60891	27.338	16.86	6
P42224	27.228	21.24	6
Q15631	27.207	22.73	5
P51648	27.202	14.93	5
Q92667	27.199	13.6	6
O95573	27.113	14.39	7
Q9Y5A9	27.063	14.7	4
Q01469	27.06	19.14	5
Q9BXW7	27.043	15.99	5
Q9C0C9	27.035	17.94	6
P49748	27.005	18.8	6
Q16204	26.978	10.26	6
Q8WUA2	26.973	11.51	5
P28288	26.916	13.09	5
P62266	26.912	32.55	4
Q9H773	26.891	21.05	6
O00273	26.867	17.35	6
P52565	26.866	20.42	5
CON_ENSEMBL:ENSBTAP00000037665	26.86	15.94	6
Q53GQ0	26.855	16.09	5
Q9UHD8	26.85	22.34	7
P23368	26.806	14.38	6
O60701	26.757	18.01	6
Q13185	26.715	15.95	4
P05783	26.693	27.84	8
Q96T37	26.669	11.16	10
Q9NPH2	26.656	19.03	8
P26358	26.585	14.04	9
Q2NL82	26.583	35.42	5
P50213	26.583	12.95	5
P23588	26.581	15.13	4
Q99767	26.565	18.64	5
O43684	26.554	35.57	5
O14745	26.511	12.24	4
P48739	26.447	19.17	6
[illegible]	[illegible]	[illegible]	[illegible]

Q9UNS2	26.408	19.62	4
Q8N163	26.385	17.48	7
P35237	26.345	21.41	5
Q02413	26.344	9.5	5
Q9UKK9	26.317	22.89	5
P50502	26.308	36.43	4
Q13228	26.278	16.99	6
Q15427	26.268	22.82	4
Q9H9B4	26.235	28.16	6
O75369	26.187	16.94	8
Q9H583	26.171	11.97	8
Q8WVM8	26.132	19.55	4
O75569	26.11	15.97	4
Q9HC38	26.109	17.04	6
Q8N9T8	26.104	22.7	5
O43815	26.031	10.06	5
Q9BVJ6	26.013	14.89	6
CON_P12763	26.01	23.32	4
O00629	26.004	12.85	5
Q15464	25.96	9.5	6
Q8NI60	25.958	11.54	4
P55145	25.933	20.96	7
P49756	25.928	18.15	6
Q9Y3T9	25.924	21.84	7
O43681	25.796	24.76	5
P23919	25.761	20.55	7
P62280	25.746	28.93	7
Q96AC1	25.665	14.07	5
P51571	25.647	22.07	4
Q13057	25.64	15.08	5
P51570	25.583	14.66	3
Q8WXF1	25.579	20.72	6
P13861	25.578	18.71	5
Q96ST3	25.49	10.82	7
Q13123	25.459	13.04	6
P06753	25.429	12.87	6
Q14527	25.413	11.68	8
Q16513	25.409	14.41	6
O43290	25.394	16.78	7
P83731	25.364	17.63	5
P35250	25.327	9.51	6
O75821	25.284	16.41	4
Q9Y6A4	25.278	21.59	5
P62979	25.277	19.82	5
O43491	25.253	18.37	7
Q96IJ6	25.201	7.08	5
Q14258	25.189	16.04	6
[illegible]	[illegible]	[illegible]	[illegible]

Q9P287	25.139	22.08	4
Q9UBU9	25.111	18	7
Q9BRJ2	25.091	9.26	6
P31944	25.068	20.49	4
Q7Z2T5	25.065	8.89	5
P48556	25.041	23.67	6
Q8TCT9	25.038	6.7	5
P30040	25.033	21.79	7
P17612	25.022	16.2	6
Q9UJS0	25.006	18.62	7
Q9UM54	24.972	11.65	6
P29692	24.968	25.55	5
Q9NQ55	24.956	12.93	5
O60488	24.932	22.34	5
P17480	24.914	17.68	7
P11171	24.902	13.16	7
P52888	24.894	17.6	7
P48735	24.893	12.99	8
P98175	24.885	18.74	5
Q8N4V1	24.873	8.99	3
Q6DKI1	24.854	14.45	5
O75746	24.84	13.92	8
Q9Y6C9	24.832	18.99	6
P20340	24.823	24.19	5
P61962	24.808	21.09	3
Q9H845	24.807	15.57	6
P51858	24.803	13.74	4
Q9BPU6	24.722	10.74	4
Q15907	24.711	17.97	6
Q16629	24.55	41.11	5
Q9Y2R9	24.527	13.45	5
P54727	24.503	18.08	5
Q13547	24.436	13.54	4
Q9P015	24.434	11.84	6
P26583	24.388	10.65	4
P12270	24.386	15.09	5
P31350	24.327	20.56	4
P28340	24.297	12.56	5
Q07020	24.282	46.17	6
Q9Y5M8	24.27	19.42	6
P43897	24.267	10.1	5
Q9H4M9	24.249	11.17	5
P78417	24.24	23.61	7
Q92615	24.195	14.72	6
CON_P02663	24.19	23.6	5
Q9BYT8	24.189	15.95	6
P13674	24.168	9.99	6
[illegible]	[illegible]	[illegible]	[illegible]

Q9GZT8	24.097	29.94	5
Q9H8H2	24.075	14.48	6
Q9NZT2	24.071	15.25	5
Q7Z460	23.998	9.23	5
Q13867	23.995	12.7	3
P26373	23.975	38.92	6
P82673	23.917	22.26	4
P49903	23.916	7.42	4
Q13423	23.888	12.08	6
Q9BVI4	23.879	13.6	7
Q5JWF2	23.85	22.97	7
P57721	23.76	21.43	4
P42696	23.753	11.58	7
Q68E01	23.752	14.94	3
Q86UE4	23.735	12.53	5
P19525	23.729	14.16	4
Q8NEY8	23.728	18.91	4
Q9BV44	23.687	13.56	6
O14737	23.66	15.99	4
Q12849	23.658	10.28	4
P18031	23.646	17.07	6
P41240	23.645	8.89	7
Q9BQA1	23.641	22.08	5
Q9NVV4	23.636	11.45	5
Q96M27	23.595	14.13	4
P63173	23.585	29.42	5
Q9BPW8	23.584	16.53	5
Q9UHG3	23.57	13.92	6
O14617	23.567	6.59	7
Q9NZ45	23.563	10.85	4
Q9NR31	23.56	15.85	4
P51532	23.539	7.46	5
Q15437	23.531	22.46	6
Q9ULC4	23.478	8.89	4
P22033	23.451	12.45	6
O43719	23.413	22.15	4
Q13428	23.411	17.52	5
Q9Y520	23.408	25.9	6
Q9BVK6	23.384	22.82	5
Q99590	23.353	10.46	6
Q7Z794	23.345	12.24	4
Q6NVY1	23.3	16.38	6
Q14558	23.263	7.58	4
P00568	23.227	17.86	6
Q13136	23.222	13.19	5
Q96P70	23.201	15.86	5
Q96KG9	23.174	8.01	5
[illegible]	[illegible]	[illegible]	[illegible]

P36542	23.157	16.6	7
Q9Y3D9	23.151	23.94	5
Q13769	23.146	12.28	5
O00267	23.137	9.15	5
P33316	23.135	16.03	4
Q9H3U1	23.128	11.06	5
CON_P02754	23.124	28.71	4
Q7L576	23.107	18.45	6
Q09666	23.071	11.9	6
Q9H0C8	23.031	16.29	6
O75152	23.014	17.26	5
Q5T653	22.982	18.44	4
Q7Z739	22.979	20.93	4
O60313	22.974	11.25	7
Q9BXS5	22.969	10.9	6
Q8ND56	22.969	22.83	7
Q15555	22.966	14.8	4
Q13242	22.943	17.24	6
Q9NR45	22.942	18.18	3
O43809	22.902	25.05	4
Q9H2G2	22.897	10.37	4
O43747	22.821	10.27	7
Q96FW1	22.81	20.5	4
P60866	22.809	22.54	3
Q9UNF1	22.789	24.22	7
Q07157	22.709	7.93	6
P48147	22.676	9.75	6
O15294	22.593	7.41	6
Q14141	22.561	23.72	5
P61758	22.555	11.89	6
Q9H2P9	22.474	19.52	3
Q99961	22.449	22.38	3
Q86W50	22.442	10.89	4
Q9Y305	22.387	4.1	7
P13804	22.382	8.93	4
Q9Y6E2	22.376	12.55	6
O14950	22.375	20.93	6
Q9Y6G9	22.372	12.48	6
Q9P2R7	22.322	7.74	6
P05787	22.247	33.43	5
Q6NSI4	22.23	13.92	5
Q86Y56	22.215	8.23	5
O94925	22.158	8.47	4
O00442	22.153	12.93	3
Q9NUQ3	22.13	14.54	5
P33992	22.117	24.87	7
Q8WUD1	22.048	33.92	4
[illegible]	[illegible]	[illegible]	[illegible]

P04792	22.013	20.82	5
Q08209	22.009	13.01	6
Q15738	22	10.39	3
P02768	21.998	34.32	4
Q04917	21.992	35.53	4
Q7Z4W1	21.977	7.24	4
Q8WWY3	21.972	9.61	4
P13693	21.967	8.56	4
O15397	21.902	9.65	5
Q16851	21.897	13.29	6
P99999	21.896	18.36	3
Q9BZE1	21.884	11.06	6
Q66LE6	21.828	19.63	3
Q9UKN8	21.826	10.04	5
O60884	21.814	7.41	3
Q9UKG1	21.812	9.77	5
P10155	21.778	18.38	5
P52306	21.747	15.42	6
O95232	21.676	17.28	4
Q00534	21.666	12.58	5
Q9BTD8	21.654	11.13	4
O15460	21.624	13.58	3
Q9P289	21.623	14.88	5
Q6NUK1	21.577	21.08	7
Q7Z4Q2	21.568	16.76	5
P33240	21.563	18.06	5
O75937	21.547	14.83	4
Q9Y4W6	21.518	10.82	7
Q9UNH7	21.517	14.25	7
Q99575	21.512	17.46	4
Q9UBM7	21.51	13.27	4
O60812	21.478	29.29	5
Q15418	21.421	14.1	5
Q9GZZ1	21.412	13.8	5
Q9BX68	21.382	9.55	2
Q86TX2	21.378	12.89	6
Q9Y5P8	21.347	14.31	6
Q8IWX8	21.311	6.25	5
P23786	21.303	12.52	5
Q14011	21.298	14.99	3
P54709	21.285	11.64	5
Q02750	21.272	7.6	4
Q9Y3C1	21.271	13.07	5
O43488	21.245	18.98	4
Q6UXN9	21.245	16.52	4
Q6P1J9	21.237	9.62	6
P12532	21.19	15.59	6
[illegible]	[illegible]	[illegible]	[illegible]

Q9H7Z7	21.163	13.44	3
P62316	21.16	27.99	4
Q9UPN4	21.074	11.01	5
Q9UI10	21.021	9.44	3
Q8IX01	21.002	14.15	5
Q8NF37	20.995	7.44	5
P20073	20.943	18.09	5
O60568	20.927	14.27	5
Q92820	20.909	11.26	4
P24941	20.906	25.61	4
Q13404	20.844	20.51	4
Q7L1Q6	20.841	13.95	7
O43252	20.832	15.51	5
Q9NPI6	20.813	10.71	4
Q9BSH4	20.806	11.84	4
P50402	20.795	14.32	6
Q9NTZ6	20.794	9.57	7
Q01650	20.794	9.26	3
O43660	20.765	9.9	4
Q5RKV6	20.765	7.22	4
P14550	20.75	17.83	4
Q96EK5	20.744	8.58	4
Q7Z434	20.673	10.73	4
Q15386	20.658	7.1	8
Q16698	20.649	13.16	5
Q86TB9	20.629	11.02	5
P61006	20.568	44.32	4
Q5JRX3	20.544	12.81	4
Q9NUD5	20.54	8.97	3
P14735	20.524	10.56	8
Q96L92	20.487	8.71	6
P62851	20.475	54.51	4
P41567	20.459	10.23	4
Q9UKM9	20.439	15.49	6
Q9BS26	20.421	16.55	4
Q86TI2	20.416	10.59	5
Q9Y580	20.394	8.82	5
P29372	20.38	10.59	6
Q13523	20.377	5.11	4
P30046	20.372	7.56	4
O43169	20.365	25.75	3
Q9BT78	20.354	11.18	3
Q9H857	20.333	8.02	5
Q9Y3U8	20.285	28.76	3
Q9UBQ0	20.231	11.89	4
P25440	20.211	11.95	7
P51151	20.205	6.42	4
[illegible]	[illegible]	[illegible]	[illegible]

Q7L5N1	20.177	10.54	5
P31483	20.135	11.38	4
Q9UJA5	20.128	10.67	6
O15173	20.077	12.32	4
Q02818	20.075	9	5
Q9NQG5	20.072	11.13	4
Q96TA1	20.066	6.65	3
Q01433	20.06	15.19	5
Q9UH65	19.993	9.11	5
P25325	19.989	24.71	2
Q9NX63	19.98	10.16	4
P78540	19.891	10.65	3
P13798	19.888	13.84	4
Q9UHD2	19.859	6.5	5
Q8WTT2	19.855	13.54	3
Q07866	19.851	16.98	5
Q5T440	19.841	8.54	5
Q9NVN8	19.83	14.45	6
O43324	19.823	11.99	6
P24534	19.792	22.92	3
P06730	19.781	15.14	6
Q8TC07	19.767	11.64	5
Q9BU76	19.748	8.48	4
Q9H3N1	19.741	14.28	6
O00139	19.721	14.64	7
Q03701	19.72	7.17	5
P54132	19.711	6.81	6
Q9BY77	19.708	18.91	3
Q9Y5Y2	19.701	13.92	4
Q8IXB1	19.68	16.08	5
P78316	19.666	5.75	4
Q13126	19.66	10.92	4
O60888	19.658	10.45	2
Q9UIA9	19.657	8.06	5
O43399	19.65	8.37	4
Q8IXM3	19.639	12.28	5
P61586	19.634	26.64	4
P45954	19.626	12.89	6
A0A0B4J2D5	19.605	12.62	3
Q96BW5	19.568	9.93	4
Q9Y5K5	19.567	11.44	5
P61254	19.563	30.68	6
P30048	19.523	17.92	5
Q13617	19.516	9.36	5
Q6P3W7	19.509	5.73	4
Q96PU8	19.503	14.94	4
Q9GZT3	19.474	14.94	4
[illegible]	[illegible]	[illegible]	[illegible]

Q96RP9	19.429	18.9	5
Q16222	19.427	8.63	4
Q9Y5L0	19.426	9.91	4
P62899	19.403	17.54	4
Q9Y3E0	19.383	15.52	3
Q14696	19.322	7.61	5
Q13564	19.307	10.97	5
Q00403	19.303	11.86	4
Q04206	19.278	13.64	4
O43592	19.258	9.01	6
Q15286	19.194	39.8	4
Q13823	19.173	13.06	6
Q9NZW5	19.1	16.99	5
Q02809	19.084	9.38	6
P20810	19.084	5.83	4
Q16527	19.03	9.31	3
P55039	19.026	9.21	4
Q9BTE3	19.002	8.45	5
CON_A2A4G1	18.992	28.7	6
Q15269	18.975	15.36	5
Q96C36	18.965	7.47	3
Q9UBV8	18.915	4.63	5
O00767	18.911	18.04	3
P21266	18.901	6.95	6
P24666	18.87	11.41	3
Q8TBC4	18.84	11.43	3
O00411	18.834	11.11	4
P47985	18.807	14.17	4
Q9Y5X3	18.788	13.99	5
Q96I25	18.777	5.2	5
P17509	18.769	13.94	5
Q14669	18.748	7.24	5
Q9BSC4	18.704	10.69	5
Q8NBJ4	18.656	15.69	4
P00167	18.653	9.6	3
Q9UK61	18.549	10.96	7
O75439	18.521	9.43	3
Q5T8P6	18.514	9.24	5
Q15185	18.513	10.55	4
Q8WX92	18.499	15.21	5
Q92541	18.44	10.17	5
Q8N1G2	18.436	11.45	7
P49643	18.423	10.28	3
Q15369	18.411	3.99	3
Q9Y376	18.408	13.94	6
Q8TDN6	18.406	12.84	3
O00505	18.361	10.17	3
[illegible]	[illegible]	[illegible]	[illegible]

Q03252	18.329	15.37	5
P36404	18.319	7.59	4
Q04726	18.302	6.49	5
Q00535	18.3	7.87	4
Q13619	18.266	12.12	6
Q92930	18.254	39.22	4
Q5JTV8	18.236	12.93	4
P14324	18.204	11.64	5
P55327	18.198	8.22	2
O00743	18.193	10.24	4
Q16540	18.17	22.71	2
Q7L0Y3	18.156	10.71	6
Q9Y221	18.138	10.46	3
Q8TAT6	18.118	13.41	5
P17661	18.064	17.13	4
P61020	18.017	20.17	4
P28482	18.007	14.34	4
Q14165	17.998	13.25	4
O96019	17.978	16.41	3
Q9H6T3	17.976	6.98	5
Q9NYB0	17.965	5	2
Q13425	17.956	6.2	4
Q15056	17.954	23.56	5
Q6YP21	17.953	11.43	4
Q9BYJ9	17.915	14.46	4
Q9HC35	17.914	15.76	5
P54920	17.911	11.84	4
Q96RQ3	17.898	6.19	3
Q7Z406	17.88	11.99	5
CON_P02662	17.864	20.69	3
P10644	17.851	16.29	4
Q8IYB8	17.818	11.05	5
Q13740	17.804	3.48	4
O15234	17.788	11.64	4
P04632	17.781	12.46	4
Q6UN15	17.776	17.07	4
Q9NRN7	17.743	6.88	5
Q9UL25	17.738	13.57	4
Q14232	17.731	11.41	4
P55196	17.728	4.79	5
Q9Y3E5	17.669	10.83	2
Q9P0J0	17.664	10.57	4
Q92542	17.655	12.43	4
P18583	17.652	7	4
O60502	17.648	4.05	6
P54619	17.612	10.38	4
Q8IWS0	17.592	6.5	4
[illegible]	[illegible]	[illegible]	[illegible]

Q99956	17.578	11.39	3
Q16822	17.572	10.98	4
O43865	17.529	17.36	3
Q86W42	17.501	13.51	3
P29966	17.494	13.27	2
O60664	17.481	16.98	3
Q9NTM9	17.442	7.02	4
CON_Q9TTE1	17.42	10.14	4
P39687	17.414	23.96	5
P46821	17.412	7.41	3
P61353	17.394	17.48	4
Q8TBX8	17.353	5.87	3
P84098	17.344	14.82	3
Q9NVE7	17.343	6.79	5
Q9NRF8	17.325	15.8	4
Q13330	17.316	7.91	5
P13073	17.301	24.15	4
Q01085	17.293	17.06	4
Q96C86	17.293	3.88	5
P61160	17.286	20.68	3
Q12792	17.277	7.99	3
Q8TCJ2	17.24	2.22	5
Q969H8	17.233	12.32	3
P21127	17.198	14.9	6
Q96CW1	17.197	9.79	4
Q9H7H0	17.194	10.41	4
Q7Z2W9	17.173	12.88	3
Q12907	17.156	10.56	4
P52815	17.134	11.01	4
O14579	17.131	10.33	4
Q96AB3	17.118	10.13	3
Q9NQW7	17.097	9.57	5
P17655	17.096	5.38	6
Q8TEQ6	17.092	6.79	6
Q6IAA8	17.075	21.25	3
Q92733	17.074	10.11	4
Q96A33	17.045	11.42	4
Q99426	17.018	10.55	4
Q8N5F7	17.004	8.73	2
Q9Y2A7	16.989	14.1	3
Q9C0B1	16.983	7.78	4
P82933	16.962	10.62	4
O00499	16.939	6.8	2
Q9BV38	16.931	15.68	5
Q9Y5Q8	16.928	10.9	4
O75964	16.92	19.2	3
Q96GX9	16.917	10.3	2
[illegible]	[illegible]	[illegible]	[illegible]

P52434	16.888	9.34	2
Q9GZT9	16.885	15.68	3
Q7L523	16.878	8.52	3
Q92905	16.862	12.91	4
Q9Y281	16.856	19.35	4
P21912	16.85	4.69	4
Q9NZM5	16.843	7.21	4
P15586	16.828	13.63	4
Q8WYQ5	16.813	11.12	4
P78346	16.805	11.31	5
Q7L3T8	16.789	7.52	4
Q8NBJ5	16.788	11.52	5
O94992	16.785	7.64	3
Q9UBF2	16.779	12.15	5
Q32MZ4	16.777	9.89	4
Q15020	16.768	8.51	6
Q13642	16.764	23.72	3
Q6IN85	16.76	7.58	5
Q12797	16.749	8.11	5
O95340	16.746	7.35	4
Q5VTL8	16.722	9.29	3
CON_P07744	16.708	16.55	4
Q9NS69	16.692	7.9	2
O60306	16.687	8.38	4
P46926	16.667	14.32	4
Q9BTW9	16.662	5.91	5
Q92796	16.652	5.05	3
Q8N8S7	16.651	8.83	4
O75955	16.64	9.26	3
Q9H9J2	16.613	6.71	5
P60983	16.589	16.12	2
CON_P41361	16.573	7.28	2
P62987	16.569	14.13	5
P06865	16.569	13.37	5
O15260	16.532	12.47	4
P46063	16.519	4.23	6
Q6YN16	16.514	10.1	2
P33908	16.513	5.63	2
Q9BRX5	16.507	10.04	4
Q5H9R7	16.495	7.4	3
O94813	16.489	9.63	3
P61326	16.486	16.23	3
Q99436	16.486	12.79	4
P49770	16.472	11.17	3
P55263	16.471	6.54	6
Q9NVX2	16.461	5.1	4
Q16539	16.442	10.85	4
[illegible]	[illegible]	[illegible]	[illegible]

P61081	16.417	17.48	5
O60610	16.388	10.26	5
Q00577	16.388	13.05	2
P48730	16.377	7.44	4
Q9Y5K6	16.364	6.03	4
Q14739	16.349	11.69	4
Q9Y2Q9	16.325	14.08	2
O14735	16.324	12.63	3
P40222	16.29	8.91	4
Q16795	16.263	12.76	3
P36639	16.262	10.35	3
Q8IVS2	16.261	13.48	3
A1X283	16.256	10.87	6
Q13557	16.233	14.07	3
Q6NUQ4	16.202	5.56	5
Q9Y697	16.184	7.18	4
P30086	16.184	6.67	4
P52732	16.183	12.34	5
Q8N543	16.135	9.28	3
Q6KB66	16.12	13.2	4
Q9NRR4	16.094	6.65	3
P61513	16.085	20.6	2
P52788	16.077	8.02	3
O75832	16.067	7.28	2
Q04695	16.045	24.69	6
P46379	16.015	8.41	3
Q9Y4P3	16.007	11.9	4
Q9UNN5	15.991	6.85	5
Q9Y223	15.967	9.95	4
Q15024	15.937	11.02	2
Q15067	15.936	9.11	3
Q9UGN5	15.933	7.79	4
Q9H223	15.927	3.47	4
Q96QC0	15.923	8.97	4
Q9NVZ3	15.917	11.76	3
O95163	15.897	11.83	6
O00170	15.89	22.02	4
Q9BRA2	15.885	9.45	3
Q86U42	15.838	12.92	4
Q13561	15.83	6.23	3
Q5JPH6	15.829	10.44	4
Q15075	15.827	6.07	5
P81605	15.823	17.85	2
P63167	15.813	8.78	2
Q92974	15.794	10.7	5
P49674	15.778	8.08	4
Q9BSJ8	15.734	5.85	3
[illegible]	[illegible]	[illegible]	[illegible]

O75717	15.719	5.39	5
Q9UJW0	15.705	8.44	4
Q16718	15.685	9.31	2
Q96HS1	15.671	10.09	3
O75400	15.635	8.24	6
Q9NUQ8	15.623	12.37	3
Q02252	15.621	6.24	5
O75312	15.606	8.52	3
Q8N122	15.603	16.9	2
O75351	15.591	5.45	4
Q9Y394	15.577	6.08	2
O75822	15.558	9.14	4
Q9H000	15.555	13.48	3
O95376	15.55	7.18	4
P50914	15.549	33.74	3
O43847	15.545	5.84	5
Q6L8Q7	15.541	10.82	4
Q9NXF1	15.538	11.56	4
P61604	15.528	23.93	5
Q7Z478	15.479	11.05	4
Q99549	15.433	7.15	3
Q15750	15.421	9.43	3
Q96FZ2	15.419	8.14	5
P10599	15.409	13.67	3
Q9UBB9	15.403	5.25	4
Q9BV57	15.373	7.61	4
Q9NVJ2	15.368	9.35	4
O15127	15.364	7.97	2
Q9UPN9	15.362	7.3	4
Q9NPJ6	15.361	6.74	2
Q9H7D7	15.356	9.44	4
Q8IZL8	15.354	18.36	3
Q96SZ6	15.345	8.95	4
Q7Z6E9	15.344	10.24	4
Q13442	15.343	9.17	4
O15235	15.34	8.38	3
Q96TA2	15.337	5.81	3
Q9H8Y8	15.328	10.59	3
Q9UBB4	15.308	8.61	4
Q9HD45	15.294	6.73	4
Q5VW32	15.287	5.53	2
Q7Z4V5	15.277	7.06	2
O75494	15.268	15.22	4
O60508	15.255	5.82	4
Q0VDF9	15.254	12.37	4
Q13363	15.252	10.26	4
P00966	15.249	8.3	5
[illegible]	[illegible]	[illegible]	[illegible]

Q6P9B6	15.208	8.8	2
Q96RS6	15.208	10.69	3
P49959	15.192	9.95	4
O43837	15.171	11.96	3
Q96HR8	15.159	16.06	2
Q6IBS0	15.121	7.43	2
Q9H9Q2	15.076	6.1	2
Q9UNX4	15.075	12.23	5
O94808	15.073	10.19	4
Q9UIV1	15.072	5.39	3
Q9UL46	15.068	6.41	2
Q9NVS9	15.065	14.05	4
P11279	15.065	8.4	5
Q15397	15.057	6.42	2
P40937	15.044	14.82	5
Q8NI36	15.042	13.72	4
Q9NR12	15.042	9.18	4
P13984	15.03	7.3	4
Q9NZL4	15.026	16.74	3
Q9BYD6	15.016	6.24	4
P20338	15.004	18.9	3
O60493	14.993	7.01	5
Q14197	14.989	10.68	4
O60547	14.974	11.56	5
O43251	14.97	8.98	2
Q14019	14.964	8.64	3
Q96IX5	14.956	13.4	2
Q9BUR5	14.948	5.44	3
Q9NUL3	14.939	7.54	5
O95758	14.923	12.49	3
Q15382	14.922	6.8	4
P30085	14.901	4.26	4
Q15042	14.886	6.98	4
Q6FI81	14.885	9.17	4
CON_ENSEMBL:ENSBTAP00000013050	14.877	11.04	3
Q9P032	14.876	10.27	3
P43307	14.874	8.04	2
Q71UI9	14.838	20.59	4
Q9UNE7	14.813	7.86	4
O00165	14.778	9.42	4
Q96A35	14.778	7.54	3
Q96I59	14.76	2.21	4
P06396	14.707	5.47	3
Q13131	14.698	6.9	4
P57678	14.686	3.8	5
Q8TEA8	14.677	11.97	2
P08579	14.669	9.69	3
[illegible]	[illegible]	[illegible]	[illegible]

Q9UK59	14.63	6.02	3
Q9NPF4	14.583	11.33	4
Q9NX46	14.575	10.3	3
Q13451	14.574	9.17	4
P07686	14.571	18.02	5
Q15274	14.565	14.29	3
Q99627	14.551	5.74	3
Q14344	14.539	14.88	3
P47813	14.505	5.29	2
Q92878	14.5	5.92	5
P62854	14.488	16.22	2
Q9UG63	14.463	6.68	4
P61923	14.454	15.04	3
Q13190	14.424	16.84	2
P49005	14.406	6.29	3
Q13601	14.398	8.92	2
P54725	14.39	6.48	4
P52789	14.386	6.22	4
O43447	14.355	10.02	4
Q14676	14.351	9.16	3
Q9Y5S9	14.346	16.94	3
P52758	14.337	11.48	2
Q9Y3A5	14.334	5.58	6
P49458	14.326	21.75	3
O00186	14.322	8.24	4
O00487	14.315	15.31	4
Q96EP5	14.305	5.38	3
Q8WVX9	14.298	14.3	3
Q9NUJ1	14.282	8.34	4
Q8WVM0	14.278	9.54	4
Q12904	14.267	12.87	3
O14908	14.228	5.7	2
Q14108	14.208	9.8	4
Q9UL18	14.193	6.86	4
Q7L592	14.193	6.47	4
Q9BYD3	14.189	10.29	3
P51452	14.183	8	3
Q9NRK6	14.178	5.11	3
Q9BYD2	14.171	4.83	4
Q9P2I0	14.144	7.76	6
Q15003	14.122	15.83	3
A0A087WW87	14.108	76.26	2
P11233	14.104	8.16	3
P05556	14.103	5.17	5
Q13148	14.099	5.87	2
P56545	14.071	9.62	3
O95219	14.058	5.4	3
[illegible]	[illegible]	[illegible]	[illegible]

Q5VTE6	14.048	6.35	2
CON_P02666	14.032	13.3	2
Q9ULA0	14.024	13.94	4
Q7L9L4	14.018	4.61	3
P12931	14.006	10.3	5
P55212	14.005	7.6	3
Q15785	14.003	4.74	4
P85037	13.997	2.64	4
Q9NWQ4	13.985	4.55	4
P62304	13.982	11.99	2
Q5JXB2	13.969	11.4	4
Q5TC82	13.966	5.84	5
Q9UHD9	13.952	9.52	5
Q5JPE7	13.936	6.36	3
P49642	13.934	13.18	3
Q7Z4G4	13.919	6.64	2
P52701	13.919	8.91	3
P10398	13.902	8.92	3
P30049	13.901	11.66	2
Q8WW59	13.895	7.69	6
Q14966	13.86	12.96	4
Q66K74	13.856	8.54	3
O75208	13.84	6.02	2
Q86YP4	13.84	10.35	3
Q6PJT7	13.839	16.24	4
Q13206	13.822	9.74	3
Q9Y237	13.812	19.52	2
P53602	13.802	6.11	5
Q8NBJ7	13.8	6.76	4
Q96DH6	13.795	13.55	2
Q13492	13.792	4.35	4
Q3ZCQ8	13.788	15.43	3
P08240	13.754	3.89	5
O95749	13.74	5.05	3
O95563	13.734	5.67	3
O60716	13.706	13.66	5
Q9NX14	13.684	7.5	1
Q9H9Y2	13.68	4.43	3
P62942	13.675	13.36	2
P49137	13.67	4.98	4
Q13409	13.665	5.48	3
Q12986	13.662	3.97	4
A8MXV4	13.652	6.61	3
Q14CX7	13.636	6.59	2
Q5SW79	13.628	5.28	3
P21283	13.627	4.52	4
P30533	13.627	7.38	3
[illegible]	[illegible]	[illegible]	[illegible]

P35611	13.604	9.31	3
Q9NVH2	13.602	4.89	3
P48723	13.586	7.77	3
P60033	13.578	15.06	2
P61225	13.558	12.35	3
Q5T3I0	13.558	9.22	3
O43913	13.558	4.17	3
Q6P1M0	13.549	7.59	4
Q9BSV6	13.521	10.01	3
P13807	13.506	9.79	2
Q9UEY8	13.495	11.65	3
Q15758	13.494	10.57	3
Q8NDT2	13.492	6.91	4
Q14315	13.464	10.1	3
Q9Y5P6	13.461	6.17	4
O94826	13.441	10.72	5
Q9P275	13.441	7.98	3
Q9NPQ8	13.437	10.2	4
Q92922	13.427	13.72	4
P62820	13.414	39.75	3
P14174	13.41	34.1	3
Q6IA86	13.396	10.46	3
Q9UPN6	13.384	6.39	4
Q92917	13.369	11.96	3
P30740	13.353	5.47	4
O75381	13.335	2.74	1
O95801	13.324	10.19	3
P09543	13.321	8.09	4
Q9HAV4	13.307	10.75	3
P51809	13.302	11.39	3
Q8WV44	13.301	6.69	2
Q9Y4W2	13.298	9.61	3
P35659	13.277	7.3	4
Q96QV6	13.276	16.96	2
Q8WXD5	13.268	9.03	3
Q9BWU0	13.249	6.19	3
Q6DKJ4	13.241	3.09	3
P35613	13.232	22.7	3
Q9H7E9	13.221	6.73	2
P53004	13.194	3.61	5
O95292	13.19	14.48	2
Q9GZM5	13.189	4.3	1
P26885	13.145	6.77	2
Q9Y570	13.142	14.84	3
Q16777	13.112	16.33	3
Q9NR50	13.097	8.16	5
P46779	13.086	10.23	3
[illegible]	[illegible]	[illegible]	[illegible]

Q8NCN5	13.068	6.4	5
P62873	13.067	20.09	3
Q00013	13.058	13.14	3
Q9UBI9	13.051	6.3	3
Q15417	13.047	10.73	4
Q5JTY5	13.046	8.55	2
Q9HCD5	13.044	2.05	3
Q9H0H5	13.026	6.06	5
Q9NVH0	13.014	4.96	3
O15126	13.014	7.36	2
Q14160	13.011	5.18	3
Q08378	12.994	2.55	5
Q9BUL8	12.952	2.94	3
Q15031	12.938	4.02	4
Q86UW6	12.928	4.29	4
Q9H444	12.908	12.11	3
Q9Y2T2	12.896	4.39	5
Q9NXG2	12.895	9.18	3
P48729	12.894	11.4	4
Q96FV9	12.876	8.5	3
Q04727	12.873	6.38	4
Q9NPD8	12.862	8.95	4
P60660	12.852	10.51	3
Q5SSJ5	12.847	7.55	4
Q9UBI1	12.831	5.73	2
Q8IX18	12.821	5.53	2
Q9BRJ6	12.818	3.52	2
O15400	12.814	4.81	2
Q9NRG9	12.81	8.91	3
P52594	12.8	3.77	1
Q96KA5	12.793	13.68	3
Q15836	12.78	16.13	1
O95372	12.747	6.1	1
Q9UHR5	12.724	7.99	3
Q9NYV4	12.718	10.18	4
Q96J01	12.716	2.02	3
O95905	12.715	3.47	2
Q9UII4	12.68	6.04	3
P82921	12.666	7.13	3
Q8TD19	12.654	5.5	4
P35080	12.652	7.49	3
P27144	12.636	7.17	4
A6NDU8	12.608	6.58	1
O43765	12.507	7.46	4
Q9Y2U8	12.506	7.21	3
Q14692	12.484	5.76	5
Q96FX7	12.449	5.7	2
[illegible]	[illegible]	[illegible]	[illegible]

A4D1E9	12.368	6.56	4
P28161	12.333	4.33	4
Q9UI26	12.322	6.19	2
P09525	12.315	0	4
Q9UBS4	12.307	10.3	3
Q5VTR2	12.284	6.83	3
Q9UGP8	12.279	7.77	4
Q92696	12.258	3.7	5
Q9Y3B4	12.249	6.33	2
Q5D862	12.237	12.64	2
Q69YN2	12.222	11.43	5
P62318	12.204	15.63	2
Q9NYK5	12.19	4.36	4
P49406	12.186	4.62	4
O14733	12.17	5.1	3
Q9Y6Y8	12.169	7.11	4
Q8TAQ2	12.165	5.22	2
Q8WVC0	12.146	5.44	2
P13051	12.13	4.98	4
Q9UPP1	12.123	6.17	4
Q9Y512	12.123	6.99	3
Q15102	12.105	10.9	4
O43929	12.103	4.26	3
Q13576	12.08	6.29	4
P23193	12.074	7.6	2
Q8IVD9	12.042	10.49	3
Q96SU4	12.042	9	3
P36915	12.019	2.53	4
O60762	12.007	9.82	3
Q9BY67	12.005	5.21	3
Q9P0L0	12.005	20.77	2
Q13405	12.005	5.24	4
P84103	11.999	20.81	3
P35270	11.991	5.19	2
Q86U06	11.99	6.02	4
P98179	11.986	4.79	1
Q9Y3C4	11.965	12.14	1
Q9BRG1	11.958	5.62	4
P11766	11.952	10.36	4
Q9C0C2	11.933	2.38	3
Q9UIC8	11.931	5.59	4
Q92747	11.916	8.41	5
Q9NVI1	11.908	4.58	3
Q9HAN9	11.907	12.3	3
O75179	11.896	7.14	3
Q9Y6V7	11.882	14.06	3
Q9UBQ5	11.882	8.06	3
[illegible]	[illegible]	[illegible]	[illegible]

Q8N6R0	11.857	6.01	5
O75306	11.85	11.24	5
Q99614	11.841	7.92	3
Q9Y2I1	11.838	7.33	2
Q7Z4H3	11.831	5.08	3
Q15126	11.813	4.7	3
P82912	11.799	15.15	2
P11047	11.795	11.13	2
Q5TC12	11.785	6.54	3
Q6SJ96	11.781	3.73	1
P63220	11.771	2.87	1
P60953	11.748	11.52	2
Q9NRZ9	11.748	3.96	3
Q5VYK3	11.718	3.62	4
P42765	11.669	2.69	3
Q7Z3B4	11.656	6.32	3
Q9ULV4	11.651	6.07	2
Q12926	11.644	8.87	3
Q9Y5Z4	11.617	8.35	2
Q15257	11.612	7.29	4
Q9BTY7	11.605	3.95	3
O15269	11.596	5.3	2
O75190	11.592	10.03	2
Q9BWS9	11.571	1.77	3
Q5T6V5	11.557	5.42	3
Q8WUA4	11.513	3.65	3
Q15291	11.502	4.65	3
Q9NUP9	11.496	4.52	3
Q9BZF1	11.49	5.21	3
Q9NWK9	11.48	3.54	4
O94766	11.474	5.26	2
P0CG08	11.469	5.22	2
Q9NWY4	11.466	11.11	3
O60841	11.465	7	3
P42677	11.461	8.88	2
Q05048	11.447	7.45	4
Q8IV08	11.437	8.86	4
Q6UWP8	11.435	4.64	1
Q9Y3D7	11.435	11.56	2
Q14168	11.43	6.06	3
Q8NHQ9	11.421	4.59	3
P04080	11.419	5.55	2
Q8NCW5	11.414	4.97	3
P36543	11.396	17.3	3
Q9UKF6	11.393	3.83	4
Q99471	11.374	15.79	2
Q9H4L4	11.371	3.98	3
[illegible]	[illegible]	[illegible]	[illegible]

Q96D46	11.366	5.09	4
Q96KB5	11.352	9.51	4
P63279	11.312	10.6	4
P53992	11.282	4.23	4
Q9BRP4	11.258	8.98	3
P40763	11.256	5.03	2
P58546	11.252	8.4	1
Q71DI3	11.246	4.14	2
Q8TCD5	11.237	5.36	3
Q9NUB1	11.204	4.79	3
P53365	11.198	3.64	3
Q9NRY5	11.197	3.73	2
O43432	11.194	5.09	3
O15382	11.187	7.13	4
Q9Y5B8	11.186	11.74	3
Q9BYB4	11.165	3.55	4
Q9HAB8	11.128	9.94	3
Q9NPA0	11.127	4.9	3
P50583	11.126	8.25	2
Q96QD8	11.124	4.88	1
Q8WWC4	11.121	7.32	3
Q5VWZ2	11.114	7.62	3
P14859	11.1	6.16	2
Q9NX20	11.098	10.04	2
Q8WU79	11.091	9.35	3
Q96N66	11.078	2.78	2
Q969Q0	11.062	7.88	2
Q12899	11.057	6.25	3
Q92530	11.051	15.82	2
O15143	11.05	6.47	2
Q99598	11.03	5.66	4
Q9HAZ1	11.015	4.4	2
Q8WUX9	10.999	6.49	3
P50453	10.975	8.84	3
Q32P28	10.965	6.03	3
Q9UN81	10.952	5.59	3
Q8IXI1	10.944	5.1	3
O00178	10.929	6.46	3
Q9NQT4	10.929	4.64	3
Q9H2W6	10.924	6.63	2
P27361	10.921	8.25	4
P22307	10.92	5.94	3
Q9BQG2	10.9	4.99	1
Q9UKX7	10.891	5.51	2
Q08752	10.883	5.86	3
Q8TED0	10.882	8.95	3
Q96T88	10.858	1.71	3
[illegible]	[illegible]	[illegible]	[illegible]

P62847	10.851	6.8	2
Q86X55	10.847	4.6	3
Q2M389	10.841	5.36	3
P35658	10.833	5.32	2
Q9NP79	10.811	7.64	3
Q9BUI4	10.805	6.7	3
P35914	10.801	5.05	2
Q99797	10.788	4.99	3
Q86XZ4	10.776	2.94	2
Q9H6R0	10.769	7.62	4
P49247	10.768	9.11	2
Q5T280	10.755	8.79	2
Q86VN1	10.752	4.9	3
Q9H0U3	10.734	6.03	3
Q9H9Y6	10.727	4.06	2
O14964	10.726	5.49	2
O60573	10.705	5.62	2
Q9Y4Z0	10.689	2.07	3
Q92890	10.687	14.13	3
Q13546	10.68	3.99	3
P05089	10.658	4.3	5
O95218	10.657	2.65	2
Q7L5D6	10.626	4.04	3
P42766	10.625	21.31	3
Q96K17	10.622	5.94	2
Q07812	10.615	4.32	1
P62312	10.613	11.82	3
P53007	10.585	9.86	4
O14880	10.582	5.09	2
Q9Y399	10.581	3.79	4
Q9GZP4	10.563	4.72	5
Q9NXW2	10.552	4.01	3
Q13868	10.545	12.18	2
Q66K14	10.542	6.02	3
P36405	10.522	5.19	4
Q9UNP9	10.522	6.57	2
Q99986	10.52	3.9	4
P42785	10.516	2.96	2
Q86WJ1	10.508	8.15	3
Q8WVY7	10.5	7.77	3
Q9BRX8	10.498	8.68	2
Q14241	10.496	4.57	2
O14874	10.494	3.8	1
Q9H0W8	10.486	4.36	1
Q9BZF3	10.48	6.47	3
O75880	10.478	6.81	2
Q9UMY4	10.474	5.76	3
[illegible]	[illegible]	[illegible]	[illegible]

Q9NYL9	10.462	5.47	2
O94979	10.462	7.76	4
Q9BZI7	10.442	6.35	3
Q9BRZ2	10.43	4.79	3
P0DP25	10.428	3.27	1
Q9NRX4	10.416	4.13	1
Q9BQ75	10.409	6.78	3
O75688	10.407	4.05	3
Q9NUQ9	10.398	17.68	2
Q6NXE6	10.391	4.2	3
Q13685	10.389	3.54	2
Q9BV20	10.369	5.62	2
Q9Y263	10.35	4.81	3
Q9H3P2	10.341	5.02	3
Q9P031	10.336	2.72	3
O43156	10.331	6.16	2
Q2T9J0	10.328	4.99	2
O95817	10.327	10.54	2
Q9Y4E8	10.327	6.46	4
Q6NYC1	10.326	6.42	4
O75531	10.313	13.7	2
P55735	10.298	7.56	2
Q9H2M9	10.297	5.94	4
Q96P11	10.289	6.22	3
O95777	10.275	7.76	2
O43395	10.264	6.45	3
O75792	10.252	9.91	2
Q9NVR7	10.241	1.71	5
P25398	10.23	14.13	3
Q9UGI8	10.216	4.41	4
P56537	10.196	1.95	2
Q9UHY7	10.193	5.41	3
Q02040	10.182	7.21	3
Q8WVC6	10.149	2.87	3
P51153	10.139	22.82	2
O15381	10.127	2.08	4
CON_REFSEQ:XP_001252647	10.121	3.39	4
Q15050	10.112	5.02	3
Q9UM00	10.109	8.08	2
O00764	10.104	6.99	3
P78362	10.092	6.43	3
P42771	10.083	2.29	2
P63208	10.082	10.17	3
O43237	10.066	4.43	2
Q8TB03	10.06	9.75	2
P41223	10.055	2.97	3
P49354	10.04	6.53	3
[illegible]	[illegible]	[illegible]	[illegible]

Q00765	10.027	7.85	2
Q9UGY1	10.026	4.55	2
P49902	10.009	4.84	3
P51970	10.007	7.47	3
P78406	10	5.84	3
Q99611	9.995	4.05	2
P37198	9.994	6.13	2
Q9BRK5	9.985	8.27	3
P82979	9.968	16.06	3
Q13112	9.964	4.41	2
Q8IU81	9.962	4.94	2
P59998	9.959	9.29	3
P20336	9.949	15.66	3
Q8IY67	9.945	6.03	2
Q9NXX6	9.932	4.44	3
P07339	9.929	6.01	5
Q9C0B0	9.928	2.83	1
Q96GD0	9.923	4.91	2
Q14562	9.914	4.79	3
Q8NFF5	9.878	7.05	2
Q9BZX2	9.857	7.04	3
Q15629	9.845	6.04	2
Q9BSJ2	9.84	5.48	2
P30825	9.839	11.18	2
Q9Y2R5	9.836	6.05	2
Q9Y6B6	9.836	4.84	3
O95295	9.835	3.26	2
P78310	9.827	5.18	3
P78330	9.822	8.58	3
Q9UGV2	9.822	9.38	3
Q05682	9.816	4.64	2
Q15005	9.816	5.37	3
Q6AI08	9.809	2.75	2
Q7L3S4	9.802	7.72	3
Q96C19	9.796	5.31	2
Q92830	9.793	4.68	1
Q06265	9.792	11.55	2
Q9BWN1	9.79	6.33	3
Q96DG6	9.765	8.56	3
Q8WWH5	9.763	4.92	2
Q9BVL4	9.757	2.83	3
Q9NX62	9.75	3.34	2
Q96T76	9.743	7.01	2
Q68EM7	9.729	3.32	3
O00625	9.7	4.46	2
P13716	9.681	3.07	2
Q9UJU6	9.677	5.36	2
[illegible]	[illegible]	[illegible]	[illegible]

Q9Y5V3	9.665	12.41	3
Q13033	9.652	3.29	3
Q13907	9.652	13.69	2
Q9Y2B0	9.643	5.02	2
Q9Y4P1	9.636	7.82	1
P49593	9.635	6.92	2
O75340	9.633	3.36	2
Q93034	9.625	2.56	3
Q13043	9.618	3.16	2
O75718	9.604	3.02	3
Q12789	9.599	0	4
Q99717	9.599	2.32	3
Q96DB5	9.587	4.44	2
Q9UPT5	9.587	4.49	3
A2RTX5	9.57	11.15	4
P29144	9.562	6.71	2
O15213	9.553	3.83	3
Q15942	9.542	6.51	1
Q8NBF2	9.532	4.55	3
Q9UDY4	9.528	4.99	2
Q96S44	9.52	5.02	2
O14646	9.512	4.46	5
Q9Y3B8	9.51	5.82	3
P10301	9.5	6.79	2
P62306	9.496	3.01	2
Q9NXH9	9.491	3.21	4
Q9HCB6	9.484	6.2	4
Q96GM8	9.471	7.05	2
Q13595	9.426	11.17	3
P40938	9.415	4.71	2
Q96KR1	9.414	5.58	2
Q9H814	9.411	2.21	2
O60678	9.41	5.24	2
Q13153	9.406	6.12	4
P35269	9.405	6.94	3
Q92621	9.403	2.49	2
Q9NVH1	9.397	2.3	2
Q5SY16	9.396	4.89	2
Q15404	9.379	5.54	2
Q9UNK0	9.376	3.7	2
Q8IXI2	9.374	5.1	2
P62857	9.368	7.05	2
P49821	9.366	4	4
Q9NNW7	9.361	5.84	3
Q16762	9.36	6.92	2
Q9H7B2	9.35	4.65	2
Q9BVT8	9.341	2.94	2
[illegible]	[illegible]	[illegible]	[illegible]

P82930	9.326	6.52	2
Q8WXF0	9.323	8.79	2
Q92785	9.307	5.33	2
Q9ULW3	9.304	2.21	3
Q9UJT0	9.285	2.94	2
Q9ULX6	9.276	11.88	1
P31949	9.274	4.46	2
P30626	9.274	4.59	3
O94776	9.271	0	4
Q96GC5	9.267	4.6	2
Q13416	9.266	0	2
Q86V21	9.257	4.64	3
Q9UQB8	9.246	2.34	4
Q96F86	9.242	4.56	4
Q9NQ48	9.209	4.57	2
P01889	9.185	1.99	3
Q9Y320	9.171	2.22	3
O75368	9.168	4.34	2
P01111	9.154	4.26	2
Q92620	9.152	5.91	2
O00469	9.138	2.61	2
Q7Z7H5	9.133	3.05	2
Q5QNW6	9.13	8.5	3
P51649	9.127	4.99	2
Q6IQ22	9.123	15.18	2
P50750	9.086	6.09	3
Q9H5Q4	9.085	5.71	4
Q9GZS1	9.065	6.85	2
Q3KQU3	9.065	8.71	2
P63096	9.063	14.41	2
O60231	9.056	4.69	2
P49006	9.039	5.83	1
P53350	9.034	4.01	3
O00233	9.025	4.66	2
Q8IY63	9.015	9.07	4
Q9Y314	9.005	5.14	2
Q9NRR5	9.002	8.48	2
P34896	8.985	9.14	2
O60869	8.984	2.91	2
Q9BVG4	8.981	4.45	3
Q9BW91	8.976	7.11	2
Q9Y5J1	8.975	5.76	3
Q9BTT0	8.968	6.92	2
Q9UNW1	8.967	3.01	3
O60907	8.965	2.56	2
Q96LB3	8.965	5.22	3
P46977	8.963	7.27	3
[illegible]	[illegible]	[illegible]	[illegible]

Q05655	8.957	3.42	3
P15735	8.941	4.54	2
P19447	8.94	5.66	3
Q9P003	8.901	2.81	1
O43759	8.893	5.2	2
Q9UBP6	8.888	4.15	2
Q96DV4	8.885	6.21	3
O14974	8.874	4.17	3
O43818	8.855	4.35	3
Q14BN4	8.85	2.06	3
Q96EL3	8.845	5.2	2
P40855	8.844	0	2
Q9BUT1	8.825	3.75	2
Q9H0N0	8.804	4.87	1
Q9HD33	8.782	8.33	2
P50579	8.781	6.56	3
Q9UFC0	8.772	2.14	2
Q9UP83	8.769	4.32	2
Q8N6M0	8.766	3.89	2
Q6P996	8.76	4.82	2
Q53F19	8.753	0	2
Q9NP73	8.751	6.5	3
Q9Y2H1	8.739	1.94	2
Q15070	8.716	1.83	3
Q96DT6	8.71	3.73	1
Q9NR28	8.706	3.26	2
Q8NFH3	8.7	7.09	2
CON_A2I7N0	8.695	3.88	3
P57737	8.687	3.24	2
O00461	8.681	2.09	1
Q9BRX2	8.658	2.7	3
O43464	8.656	5.13	2
P62330	8.637	6.17	2
Q9UHQ9	8.634	7.48	2
O15145	8.61	6.46	2
Q9UBC2	8.597	4.98	4
Q96T51	8.596	7.78	3
P09001	8.589	11.16	2
O43813	8.588	6.1	2
O00488	8.576	5.9	2
Q8NHP6	8.568	3.68	3
P62861	8.553	12.18	3
Q9Y4X5	8.553	2.64	3
Q9NRW7	8.549	4.36	3
Q5BJH7	8.542	6.54	2
P54646	8.52	4.95	2
Q96S66	8.519	2.4	2
[illegible]	[illegible]	[illegible]	[illegible]

Q9NYY8	8.509	4.25	3
P53680	8.502	8.57	2
Q6ZN17	8.502	4.71	2
Q3MHD2	8.489	5.01	1
P10253	8.482	2.28	2
Q10570	8.472	7.05	4
Q9HCN4	8.472	5.18	2
Q16644	8.47	1.6	3
Q9NQH7	8.469	5.79	2
Q6P4A7	8.459	0	4
Q8NHP8	8.457	4.73	2
Q9Y276	8.448	1.8	3
Q96JJ7	8.435	4.72	3
O00257	8.43	1.98	3
Q9Y673	8.426	2.91	2
Q86UL3	8.424	4.68	2
Q96AY3	8.42	3.17	1
P28702	8.418	9.39	2
Q9NW82	8.394	3.76	3
Q8N7H5	8.388	3.87	2
Q9UH62	8.384	5.51	2
Q53T59	8.38	4.64	2
Q9Y4R8	8.379	1.71	4
Q96ME1	8.378	4.15	3
O95819	8.378	5.99	3
O94913	8.377	3.57	1
P05026	8.362	2.83	3
P04637	8.361	4.52	3
Q9UMY1	8.358	5.49	2
P08754	8.358	14.57	2
Q9NT62	8.335	3.97	1
Q96CT7	8.32	7.63	3
Q9NWH9	8.299	5.19	3
P55769	8.291	7.12	2
Q9Y2P8	8.281	2.05	3
Q9UBW8	8.28	2.03	2
Q15041	8.27	5.01	2
O75391	8.239	5.97	2
Q9BUK6	8.233	0	3
Q9Y3A6	8.23	2.63	2
Q8WU90	8.22	4.63	2
O95816	8.21	12.51	2
Q9UN37	8.2	3.92	3
Q4J6C6	8.191	5.02	3
O94842	8.177	4.29	2
Q7Z6Z7	8.164	7.92	3
Q9H6Y2	8.164	3.58	1
[illegible]	[illegible]	[illegible]	[illegible]

O60524	8.151	4.56	3
P19404	8.136	6.85	2
Q96C23	8.136	2.98	1
Q9BPX3	8.122	6.19	2
Q99816	8.121	4.11	2
Q96MX6	8.115	4.04	2
Q9BYN8	8.113	5.68	2
Q8IUX4	8.094	2.39	3
Q9NTG7	8.092	4.66	2
P40616	8.092	15.15	2
Q8NBX0	8.089	4.72	2
Q9BWG6	8.088	2.02	3
Q3LXA3	8.084	7.51	1
Q9H3K2	8.079	5.91	3
Q8IXM2	8.07	6.26	1
O75694	8.057	5.93	3
Q9HB07	8.051	6.42	3
O95409	8.048	4.39	2
Q9NZ32	8.048	5.9	1
Q9UI30	8.037	3.34	2
Q9HBH5	8.036	5.06	2
Q4G0J3	8.034	4.09	3
O75608	8.033	2.48	3
Q969G3	8.021	8.91	1
P02794	8.02	5.89	3
P27986	8.01	4.1	1
P10321	8.007	2.96	2
P04921	7.997	2.8	1
Q96JB3	7.994	15.07	2
Q96KP1	7.971	2.57	3
Q9HB40	7.961	6	2
Q9H1I8	7.942	3.56	3
Q71RC2	7.934	4.33	2
O43502	7.926	1.94	3
Q9UKZ1	7.904	3.82	1
Q9UBD5	7.904	2.86	2
Q969S3	7.894	5.8	2
Q9NQC3	7.894	5	2
Q9BX40	7.889	4.23	2
Q96FZ7	7.887	4.95	1
Q9UHJ6	7.858	3.04	1
Q4G148	7.857	0	3
Q9Y3B9	7.845	2.85	2
P16383	7.838	2.13	3
Q7Z7H8	7.832	4.97	2
P11802	7.832	8.16	3
Q9H269	7.827	2.48	2
[illegible]	[illegible]	[illegible]	[illegible]

P36969	7.814	9.27	1
Q9NPD3	7.805	7.15	1
Q7L2J0	7.797	2.39	2
Q96BP3	7.794	4.17	2
P53597	7.79	9.23	1
Q9UBB6	7.785	4.47	2
Q96QU8	7.782	2.09	2
P61077	7.78	11.68	2
P55957	7.768	2.84	1
P49585	7.763	2.33	2
Q9H074	7.76	4.61	2
Q9H7S9	7.76	5.17	2
Q9NWT1	7.757	2.01	2
Q9BVQ7	7.755	3.48	3
Q13188	7.751	1.65	2
Q12765	7.743	5.15	2
Q5VIR6	7.742	3.91	2
P51784	7.738	6.23	3
Q00169	7.729	4.57	2
A1L0T0	7.713	2.64	2
Q8IZP0	7.702	4.16	3
P12694	7.693	3.44	1
Q7LBC6	7.672	5.55	3
P41214	7.666	5.82	2
P82914	7.663	4.42	3
O75223	7.649	4.33	2
O75251	7.634	6.98	2
Q9H3G5	7.627	8.83	2
Q9Y4C2	7.626	3.64	3
P47914	7.617	3.86	1
Q70UQ0	7.617	4.44	2
Q14194	7.613	9.67	2
O94888	7.609	2.4	3
Q9Y388	7.605	2.41	3
O75794	7.601	4.5	2
P33121	7.601	2.48	2
Q9BW19	7.6	6.58	2
P51553	7.591	3.89	1
P60468	7.583	4.42	2
Q7KZN9	7.581	3.45	3
P50613	7.581	4.21	2
Q8N726	7.58	4.11	2
Q9H1Y0	7.566	3.72	2
Q9Y6M9	7.565	4.16	2
Q969U7	7.547	4.33	3
P09601	7.544	0	2
O95486	7.539	4.32	3
[illegible]	[illegible]	[illegible]	[illegible]

P53367	7.526	5.15	2
Q53S58	7.526	2.07	2
O75940	7.526	7.72	2
O00159	7.504	3.79	2
O15131	7.5	3.91	2
Q9C005	7.498	3.9	1
P67870	7.478	2.81	2
P49750	7.466	5.1	3
P49407	7.466	4.18	1
A6NED2	7.441	4.55	2
O96013	7.441	5.35	2
Q92990	7.423	2.6	3
Q9H993	7.42	5.82	3
P04062	7.41	3.55	3
Q2M296	7.401	3.94	1
Q14139	7.4	3.76	2
Q9NXR1	7.392	3.41	1
Q53EL6	7.384	4.15	3
Q9NVH6	7.382	2.02	4
Q9NP77	7.378	3.27	1
O75935	7.377	4.39	2
Q8TED1	7.368	5.59	3
Q9Y5A6	7.337	2.86	1
Q96S59	7.336	2.12	1
Q9NQW6	7.333	4.79	2
Q14142	7.327	3.88	2
Q86VR2	7.321	0	2
Q9Y2G5	7.316	3.33	1
P60763	7.31	5.62	2
O95456	7.297	8.74	1
Q6P6C2	7.289	4.54	2
Q8IX12	7.287	5.52	1
P17482	7.28	5.54	2
Q9ULD0	7.277	1.99	3
Q9NWV4	7.268	2.58	1
CON_Q03247	7.262	2.18	2
Q709F0	7.262	3.99	2
Q9ULX3	7.256	6.95	3
P37268	7.241	2.2	3
Q9H9J4	7.238	4.72	2
P07203	7.232	3.42	3
Q9P2T1	7.217	4.01	2
P49773	7.214	6.18	1
Q9H008	7.206	0	1
Q29RF7	7.206	2.25	3
P60520	7.185	6.34	2
Q5JVF3	7.181	0	2
[illegible]	[illegible]	[illegible]	[illegible]

Q9UBN7	7.175	1.94	2
Q49AR2	7.156	2.5	2
P55210	7.153	2.41	2
Q15388	7.15	4.22	1
Q969N2	7.149	4.49	2
P49207	7.148	15.46	4
Q8TD30	7.144	4.26	2
P09669	7.142	3.67	3
P29558	7.137	7.01	1
Q8IZ81	7.136	7.2	2
Q9BQ61	7.124	4.16	2
O75909	7.124	2.27	2
Q9NZZ3	7.114	3.04	1
Q9H267	7.108	0	3
P62633	7.104	5.86	1
P67812	7.093	10	2
Q9Y3C6	7.082	5.14	2
Q4G0F5	7.076	4.18	2
O75419	7.073	2.15	2
P19174	7.064	4.22	2
Q9NRP0	7.061	8.94	1
Q15642	7.059	4.1	2
O43676	7.053	4.09	2
Q13158	7.047	2.63	1
Q9HAV7	7.046	3.93	3
Q4G176	7.044	2.64	2
Q9H3Z4	7.032	3.74	1
O94763	7.022	1.81	2
Q96QE5	7.01	1.67	3
O14949	7.008	4.36	2
Q96GA3	6.994	4.96	2
Q9UNF0	6.988	8.05	2
Q15059	6.983	4.54	2
Q9Y3B3	6.982	0	2
Q9Y6A5	6.978	2.81	2
O60942	6.974	1.82	2
A0FGR8	6.967	4.48	3
Q9H3P7	6.96	4.5	2
Q9Y6M7	6.957	1.85	2
P61421	6.956	3.37	3
Q9NVA1	6.955	3.29	2
Q9UJC3	6.931	1.88	2
Q01804	6.926	0	2
Q9HA77	6.923	0	2
Q9H089	6.923	3.72	3
Q13445	6.917	1.88	1
Q16778	6.891	8.77	2
[illegible]	[illegible]	[illegible]	[illegible]

O00161	6.877	3.18	1
O75695	6.862	4.09	2
Q9BRR6	6.855	4.41	3
Q8N3U4	6.851	2.4	3
Q9NX61	6.849	6.45	1
Q15428	6.847	2.48	2
O15344	6.839	3.44	2
Q9P0V9	6.836	2.83	1
P18754	6.83	5.72	3
O75915	6.829	14.04	2
P53990	6.81	3.71	3
Q9HD26	6.8	4.33	2
Q9BY43	6.796	2.6	1
Q96EY8	6.787	7.38	2
O96017	6.773	6.24	2
Q9H936	6.771	1.63	2
Q6UW68	6.767	3.48	1
O95478	6.746	5.03	2
Q9UHR4	6.742	2.33	2
P35221	6.738	2.12	2
Q8N2U0	6.735	6.68	1
Q969X5	6.732	6.31	2
Q96LR5	6.729	9.3	2
O95249	6.726	2.18	2
Q969M3	6.715	2.46	1
Q92485	6.712	5.88	1
Q13630	6.708	1.89	2
Q00796	6.7	3.31	2
Q7L099	6.7	2.07	2
Q14204	6.697	2.33	2
Q9NVU7	6.693	3.96	2
Q9NX40	6.689	4.82	2
P07197	6.676	8.38	2
Q5T6F2	6.669	6.03	1
O95169	6.654	3.39	2
Q9H488	6.653	2.94	2
Q01968	6.651	4.48	2
Q9H2D1	6.642	5.96	1
Q9BZL6	6.642	4.99	2
Q6PI78	6.633	3.31	2
CON_P01030	6.62	1.82	3
Q9NX08	6.616	2.43	1
O94966	6.603	2.09	3
Q14691	6.603	3.52	1
O00400	6.593	0	1
Q9Y3C8	6.592	3.76	2
Q16611	6.59	4.1	2
[illegible]	[illegible]	[illegible]	[illegible]

P42357	6.587	2.46	1
Q9UKL0	6.58	2.23	2
Q8NCD3	6.577	2.05	2
Q14847	6.574	9.71	1
Q15527	6.572	2.58	1
Q9NXS2	6.571	2.62	2
O60925	6.569	4.23	2
P05067	6.551	3.01	1
Q8TCE6	6.537	2.68	1
P09497	6.534	5.23	3
Q12800	6.532	2.78	1
Q712K3	6.523	3.84	2
Q9NWS8	6.515	3.52	2
O15530	6.511	1.92	2
Q96DE0	6.509	2.06	2
O94921	6.506	6.28	2
Q9BR76	6.472	1.65	2
O95059	6.471	2.76	1
Q6NSZ9	6.458	3.99	3
O95602	6.436	2.4	1
O75879	6.434	2.8	2
Q9NR77	6.433	4.59	2
Q96ST2	6.432	2.68	2
Q9Y2Q5	6.431	3.08	1
Q92522	6.431	14.1	2
O60784	6.426	5.99	1
Q5JRA6	6.415	2.54	2
A6NDG6	6.407	3.52	2
Q15796	6.407	2.52	2
Q9BXS6	6.405	3.79	2
P78332	6.401	4.08	2
Q9H4A5	6.392	5.52	1
P20719	6.391	1.61	2
P78345	6.391	3.18	1
P52294	6.389	2.11	2
Q9HAD4	6.378	4.21	2
Q9UDY2	6.377	2.08	2
P53384	6.372	2.58	1
O14548	6.365	3.19	1
O14976	6.362	3.74	1
P06756	6.356	2.94	1
Q9UKY7	6.353	0	2
Q9NVT9	6.342	3.6	1
Q96LA8	6.326	4.59	2
P25311	6.323	2.43	1
Q53GS7	6.321	3.59	3
Q9Y2Q3	6.317	5.61	1
[illegible]	[illegible]	[illegible]	[illegible]

O43819	6.31	7.97	2
Q15043	6.309	0	1
P10606	6.306	2.34	2
Q8TAA5	6.301	2.75	2
Q99700	6.298	6.27	2
Q9BTE7	6.295	4.43	2
P41743	6.291	6.26	1
Q86X76	6.289	4.75	2
P12273	6.284	1.96	2
P35222	6.279	1.75	2
Q969J2	6.277	2.83	1
O15479	6.267	4.98	2
Q9GZZ9	6.266	2.14	2
O95985	6.263	0	2
A0MZ66	6.261	1.75	2
O00566	6.252	2.45	2
Q7L5Y9	6.245	1.84	2
P30613	6.239	14.9	2
Q9P000	6.228	2.51	1
Q9P2R3	6.227	2.08	2
Q96CQ1	6.208	2.68	1
Q8WVI0	6.204	3	1
Q9BXY0	6.204	2.56	1
Q8N3C0	6.2	3.76	2
Q8WXA9	6.193	8.96	1
Q9Y3A4	6.187	2.24	1
P15374	6.187	3.73	1
Q12872	6.174	1.94	2
Q6EEV6	6.17	5.27	1
Q96HY6	6.169	7.36	2
Q14254	6.169	2.06	2
Q9BT22	6.168	1.63	1
Q02127	6.165	3.47	2
Q9H6F5	6.155	6.56	2
Q13325	6.139	3.14	1
P53634	6.129	0	1
A4D1P6	6.128	5.18	3
Q8IY37	6.107	2.32	2
Q9H0R6	6.105	0	2
P57740	6.094	0	2
Q9H2C0	6.092	4.62	2
O75150	6.088	2.56	2
O75844	6.079	3.97	2
Q9UNL2	6.063	8.31	1
Q13505	6.037	2.67	1
Q15014	6.035	2.98	1
O60333	6.031	2.44	2
[illegible]	[illegible]	[illegible]	[illegible]

Q9Y6W5	6.021	2.96	1
Q9NQE9	6.02	1.76	2
P55011	5.996	1.83	2
O75521	5.995	4.46	1
Q7RTS7	5.994	4.38	2
Q96HA1	5.975	12.5	2
Q7Z4H8	5.974	2.03	2
P05165	5.971	2.44	1
Q96EK6	5.96	2.81	1
Q9Y3D3	5.949	4.92	2
E9PRG8	5.945	1.62	1
Q9P2B2	5.937	1.71	2
P46013	5.934	2.57	2
O00401	5.932	2.22	2
Q15036	5.931	2.16	2
Q9Y3I1	5.928	2.99	1
Q02952	5.922	3.07	1
Q9BQ69	5.915	0	1
Q96I51	5.896	3.07	2
Q92600	5.895	2.59	2
Q8N335	5.893	0	2
Q99747	5.89	0	2
P84090	5.86	6.14	1
O95671	5.86	4.06	2
Q32P41	5.842	1.88	3
P61626	5.827	3.08	1
Q6PD62	5.822	2.67	1
Q7LGA3	5.808	5.33	2
O75629	5.796	3.01	1
Q3ZAQ7	5.782	14.04	1
Q8WUY1	5.77	1.64	3
Q8IZ69	5.766	1.74	2
Q92572	5.753	3.13	1
Q9H0R4	5.744	1.95	2
Q9UDY8	5.744	1.76	1
Q9H5V9	5.741	7.1	1
Q9NV06	5.736	2.11	2
O43505	5.72	0	2
Q86TP1	5.705	4.2	2
P0CG12	5.704	6.46	2
Q53H82	5.703	0	3
Q92802	5.695	2.06	2
Q08379	5.688	2.54	1
Q00059	5.683	5.94	2
Q7RTS9	5.675	1.81	2
Q13427	5.669	2.69	2
Q9BWH2	5.664	3.09	1
[illegible]	[illegible]	[illegible]	[illegible]

Q9UNI6	5.662	2.13	2
L0R6Q1	5.647	2.59	2
CON_P01966	5.642	2.91	1
O43824	5.639	2.42	1
Q9NV88	5.634	2.02	2
Q9Y6I9	5.634	2.82	1
Q96S52	5.629	7.34	1
Q8IZ73	5.622	1.84	2
Q8N9F7	5.612	2.31	2
Q5T160	5.611	1.86	2
Q9UH17	5.609	0	2
P11441	5.608	0	3
Q9H9C1	5.607	2.3	1
Q92989	5.595	1.66	2
Q99720	5.591	2.31	1
Q9Y613	5.575	3.12	2
Q8NB49	5.573	1.76	2
Q9NSI2	5.563	4.47	1
Q9NQZ5	5.561	2.4	1
O43924	5.559	3.82	2
Q8N138	5.53	1.97	2
P49810	5.524	2.65	1
Q8WX93	5.505	2.38	1
Q7KZI7	5.489	0	2
Q9H9A5	5.475	2.96	1
Q9NWS0	5.474	3.19	1
Q9BTC0	5.471	3.09	1
Q9Y333	5.468	2.43	1
Q9Y5K8	5.468	3.07	1
P61009	5.463	2.71	1
P51668	5.458	2.05	2
P53701	5.451	3.56	2
P62308	5.449	2.84	1
O95104	5.442	6.16	2
P16930	5.439	1.95	2
Q96D05	5.439	5.02	1
O00458	5.433	1.61	2
Q15007	5.431	1.7	2
Q96MW1	5.426	1.95	2
O60934	5.424	1.95	3
Q14012	5.419	0	2
Q9NPA8	5.415	0	2
Q92597	5.413	2.26	1
Q8WYA6	5.412	0	2
Q15061	5.411	7.41	2
Q9UJC5	5.407	3.06	1
P09417	5.404	4.47	1
[illegible]	[illegible]	[illegible]	[illegible]

P60981	5.398	3.77	2
Q5ZPR3	5.396	3.1	1
O43264	5.394	1.85	2
Q8N5M4	5.391	2.4	1
Q66PJ3	5.383	2.71	1
Q9UHY1	5.38	9.57	1
P49790	5.375	1.91	2
Q8NC54	5.372	2.4	1
Q99707	5.371	2.37	2
P63165	5.362	2.13	2
P32242	5.362	3.69	1
Q9BUE0	5.359	6.35	2
O95400	5.359	2.31	1
Q99943	5.355	1.86	2
Q969T9	5.349	1.61	2
O75616	5.345	4.54	2
Q13526	5.344	4.09	2
P49840	5.335	2.13	2
Q96JB5	5.335	3.79	2
P68402	5.324	5.16	1
P21926	5.323	2.79	1
Q7L8L6	5.319	3.9	2
Q92889	5.319	3.6	1
Q9NPJ3	5.317	3.57	1
Q5T200	5.314	1.87	2
Q99442	5.31	3.53	2
P62891	5.308	6.76	1
Q96HC4	5.302	2.07	2
Q86UY8	5.3	5.8	1
Q9H446	5.296	2.37	2
Q92925	5.296	1.82	2
Q9NWU5	5.288	3.02	1
Q7KZ85	5.284	4.07	2
Q9H2J4	5.279	2.2	1
O95239	5.268	1.7	2
P63172	5.266	3.91	1
Q8NDX5	5.264	2.85	1
Q9Y3E7	5.254	1.88	1
Q96AX1	5.251	1.78	3
P37840	5.249	0	2
Q6P1X5	5.238	0	1
O15037	5.23	1.73	2
Q4G0N4	5.23	3.91	2
Q96EV2	5.23	2.19	2
Q8TEM1	5.228	2.61	1
O60563	5.223	2.53	1
Q9UI12	5.218	8.59	1
[illegible]	[illegible]	[illegible]	[illegible]

Q8NEY1	5.214	1.84	2
O43617	5.214	0	2
Q8NBM4	5.207	1.96	2
Q4V328	5.191	4.05	2
O60831	5.185	8.8	1
Q8TF09	5.183	6.28	1
Q96B26	5.176	2.66	1
Q14919	5.165	2.95	1
Q9HC07	5.161	2.34	1
P35612	5.153	1.62	2
Q9BRS2	5.151	0	2
O60216	5.146	2.65	1
Q13951	5.144	4.26	1
Q96GQ5	5.139	2.45	2
P42574	5.131	3.03	1
P52298	5.129	5.54	1
Q147X3	5.126	3.54	2
Q8TEU7	5.123	3.78	2
O75648	5.119	2.51	2
P29218	5.106	0	2
Q5UIP0	5.093	2.3	1
Q14331	5.092	2.83	1
O95551	5.088	2.1	2
Q96IU4	5.086	0	1
Q9Y2I8	5.069	1.77	1
Q5W111	5.068	2.04	1
Q8IWA0	5.059	1.97	3
Q86YN1	5.054	5.17	1
O94804	5.053	1.99	2
Q92888	5.049	0	2
O75348	5.048	2.92	1
Q96RE7	5.047	4.14	2
Q9NRG1	5.041	2.75	1
Q7Z5J4	5.035	2.17	1
Q8TDX7	5.031	2.2	2
Q16763	5.026	2.48	2
Q9BVC6	5.014	4.36	1
Q9BZG8	5.011	0	2
Q9BX10	5.01	2.32	1
Q7Z7F7	5.008	1.72	2
Q8NFH4	4.996	4.75	1
Q8NBN7	4.996	3.7	2
Q5C9Z4	4.995	2.69	1
Q15334	4.989	2.85	1
Q9UEG4	4.982	2.75	1
Q14118	4.976	0	1
P00846	4.971	12.2	1
[illegible]	[illegible]	[illegible]	[illegible]

P56134	4.949	5.9	1
Q9NY61	4.944	0	1
P48651	4.941	5.23	1
Q9BXK5	4.931	2.73	1
Q96T21	4.925	0	1
A0AVF1	4.919	1.98	1
Q9H6L4	4.917	2.6	1
Q96EY4	4.912	2.43	1
Q3KQV9	4.91	2.08	2
P48449	4.899	1.75	1
Q9BUB7	4.893	7.56	1
O43670	4.892	7.59	1
P53611	4.892	8.96	1
O95197	4.888	2.51	1
O95168	4.874	2.6	1
P49336	4.872	1.64	1
Q7Z4H7	4.868	1.71	2
Q92759	4.868	1.75	2
O43633	4.863	2.2	2
Q8N6H7	4.862	2.14	1
P18887	4.854	1.96	2
Q6GQQ9	4.845	2.53	1
Q8NC60	4.844	2.6	1
Q8WW12	4.841	2.58	1
P04066	4.837	3.02	1
O15504	4.83	2.85	1
P20674	4.83	2.14	2
Q6UXH1	4.82	1.89	1
P56211	4.819	1.87	1
P35030	4.819	2.23	1
Q8N108	4.814	3	1
Q8N3Z3	4.812	1.71	1
Q9ULM6	4.789	1.88	1
P48507	4.783	3.02	1
P02100	4.781	12.25	1
P05423	4.768	3.48	2
Q9NXF7	4.767	2.79	1
P56556	4.765	0	2
Q9Y2G9	4.763	0	1
Q9NTX5	4.757	2.77	1
Q9UHV9	4.756	0	2
Q9BZJ0	4.756	1.63	2
Q9H977	4.753	5.53	1
Q9P2K5	4.741	1.84	2
P31937	4.737	4.77	2
Q9UL40	4.734	1.65	1
Q12974	4.734	2.71	1
[illegible]	[illegible]	[illegible]	[illegible]

P12074	4.731	0	1
P51617	4.719	4.14	2
Q03426	4.717	0	1
O15305	4.713	1.74	3
Q8IWC1	4.711	1.72	2
Q96T58	4.707	1.67	3
P57772	4.681	2.66	1
Q8N3R9	4.678	2.25	1
O15270	4.676	4.02	2
Q12981	4.673	1.63	2
Q8NFV4	4.668	0	1
Q96H79	4.668	2.52	1
O43542	4.653	1.97	1
Q96N67	4.648	0	1
O43298	4.647	2.06	1
P50336	4.643	2.58	2
Q5R3I4	4.638	2.49	1
Q15018	4.637	1.9	1
P80303	4.626	4.37	1
P07902	4.621	2.19	1
Q96GK7	4.62	1.99	2
Q8NEJ9	4.619	0	1
Q9BW60	4.618	1.93	1
Q8IXH7	4.615	4.03	2
Q9H9P8	4.6	0	1
Q16563	4.599	13.24	1
Q9BTZ2	4.597	2.07	2
Q07002	4.597	7.85	2
Q9BTV5	4.595	2.24	1
Q96Q11	4.593	2.47	1
Q5VV42	4.591	2.38	1
Q9H910	4.587	0	1
P18077	4.587	3.99	2
Q9ULE6	4.585	2.22	1
Q8IYB5	4.584	2.17	1
Q27J81	4.568	2.85	1
Q92520	4.555	0	2
Q92995	4.551	1.97	2
Q9UBV2	4.55	2.89	1
Q8N983	4.535	1.77	1
P05771	4.531	0	1
Q68CZ6	4.53	2.03	1
Q14671	4.528	3.83	1
Q9P0T4	4.526	0	2
Q9HCS7	4.526	2.04	2
Q9HCK8	4.523	3.52	2
P58004	4.521	7.93	1
[illegible]	[illegible]	[illegible]	[illegible]

Q9UEE9	4.514	5.28	1
Q9H944	4.512	2.12	1
O60476	4.51	2.48	2
P24390	4.505	2.29	1
Q86YH6	4.505	2.49	1
Q96T60	4.503	0	2
Q9H4A3	4.499	2.06	2
CON_P15497	4.499	0	2
Q16560	4.498	1.92	1
Q86U90	4.493	1.91	1
Q5TBB1	4.491	2.5	1
CON_Q3SZR3	4.485	2.03	1
Q9BYD1	4.484	1.95	3
Q9H2H8	4.467	2.44	1
O43708	4.459	0	1
O15321	4.456	2.66	1
P86790	4.452	2.08	1
Q6UX04	4.446	3.3	1
Q9UHA3	4.444	1.9	1
Q6PJG6	4.443	2.26	1
Q8NB37	4.437	2.45	1
Q13541	4.431	0	1
Q8N9N7	4.426	0	1
Q6P2H3	4.415	1.72	1
Q7Z2Z2	4.405	5.25	1
Q8N1G0	4.404	1.63	1
Q99417	4.399	1.77	1
Q9UMX5	4.393	1.84	1
Q92783	4.389	3.99	1
Q99759	4.384	0	2
Q9H201	4.377	2.45	1
Q9NSK0	4.368	3.33	2
Q10469	4.365	2.02	2
P28799	4.364	2.27	1
Q13464	4.363	3.6	2
P61960	4.361	4.24	1
O15118	4.361	1.78	1
P08962	4.356	3.95	1
Q12768	4.353	2.05	1
P29992	4.345	1.86	2
Q92797	4.343	2.43	1
O95470	4.34	0	2
P04179	4.339	4.4	2
Q9NRL3	4.339	1.79	1
Q8N5K1	4.33	0	2
CON_P00711	4.32	2.06	1
P62310	4.314	5.9	1
[illegible]	[illegible]	[illegible]	[illegible]

Q9NTW7	4.294	8.24	1
Q9Y5N5	4.273	2.27	1
Q8IVV7	4.27	1.64	1
O43896	4.269	1.73	2
P20645	4.262	2.1	1
P27216	4.246	1.87	2
O75352	4.246	2.04	1
Q9NRV9	4.243	2.28	1
Q9Y6M4	4.238	0	2
A8CG34	4.226	1.68	2
P52564	4.223	4.92	1
Q96HR9	4.218	2.66	1
Q32NB8	4.216	0	1
Q9H7N4	4.208	2.5	1
Q8ND04	4.186	2.19	2
P54687	4.181	2.06	2
P05109	4.175	2.16	1
P56179	4.174	2.49	1
CON_P50448	4.17	2.68	1
P57081	4.168	1.71	1
Q9NZJ7	4.166	2.52	1
P11274	4.158	1.83	1
Q08380	4.152	0	2
Q96P63	4.149	2.03	2
P57105	4.137	0	1
P03905	4.136	1.71	1
P22570	4.134	0	1
P23443	4.124	0	2
Q9NVC6	4.117	2.37	1
O75146	4.114	1.75	1
Q9P0L2	4.107	0	2
Q99633	4.104	0	1
Q9NUQ2	4.098	0	1
Q9H3S7	4.097	1.74	1
Q06546	4.097	2.31	1
Q8WUW1	4.097	2.25	1
P61764	4.095	1.8	2
Q9Y3D8	4.092	2.13	1
Q9NWX5	4.085	2.21	1
Q9BVS4	4.072	2.08	1
Q7Z569	4.068	0	1
Q9UID3	4.058	0	2
Q9BUL9	4.04	0	1
Q969E2	4.04	6.24	1
Q9NQ88	4.034	2.61	1
P21953	4.03	0	1
Q86VI3	4.03	1.99	2
[illegible]	[illegible]	[illegible]	[illegible]

Q9NXE4	4.024	0	2
Q676U5	4.024	2.51	1
Q9H4M3	4.02	0	1
Q86UE8	4.017	0	2
Q92879	4.014	2.37	2
Q9BU61	4.01	2.16	1
Q9P0I2	4.008	1.83	2
Q99653	4.006	1.86	1
O15258	4.005	0	2
P07311	4.004	2.03	1
Q14119	4.002	0	1
P16278	4.001	0	1
P52756	3.997	2.28	1
Q8N1B4	3.997	2	2
P57086	3.988	2.09	1
Q587I9	3.987	2.67	1
O43402	3.987	3.07	1
Q9Y2W2	3.983	2.32	1
Q9UKJ3	3.979	1.81	1
O14556	3.971	1.97	1
Q6ZRQ5	3.971	0	1
Q9H981	3.968	0	1
Q15121	3.967	2.69	1
Q9Y6J9	3.964	0	2
Q6GMV3	3.96	2.46	1
Q8NHH9	3.956	1.7	1
Q9H9F9	3.952	1.62	1
Q7Z7A3	3.945	2.3	1
Q9BVC4	3.933	2.43	1
Q9BY41	3.928	1.81	1
Q9Y672	3.923	0	1
Q99543	3.92	1.92	2
Q14126	3.91	2.44	1
P52655	3.908	2.98	1
Q9UFW8	3.899	5.6	2
Q12846	3.899	2.52	1
O75116	3.897	1.82	2
Q99643	3.891	0	1
Q9NZD8	3.885	1.71	1
Q9NZQ3	3.883	2.41	1
Q8IUR6	3.88	0	1
P49914	3.876	3.31	1
Q9UNH6	3.871	0	1
O43760	3.865	3.71	2
O75787	3.858	2.58	1
Q92538	3.848	0	2
Q9NXW9	3.845	3.8	1
[illegible]	[illegible]	[illegible]	[illegible]

P46976	3.842	5.07	1
O15054	3.841	1.67	1
O14925	3.838	2.3	1
P49459	3.832	2.43	1
O60826	3.822	0	2
Q8TBQ9	3.817	2.48	1
O14656	3.814	0	1
Q5SWX8	3.814	2.47	1
Q2TAL8	3.806	2.53	1
P14678	3.798	8.89	1
Q6PCB5	3.794	1.71	1
Q15542	3.791	4.59	1
O14936	3.786	0	1
Q9Y343	3.786	2.02	1
Q9NVS2	3.784	2.2	1
O76024	3.783	0	1
Q5QJE6	3.783	1.66	1
P82663	3.783	3.62	1
Q92947	3.779	2.17	1
Q9BUN8	3.768	2.62	1
Q8N954	3.768	0	1
Q9BWE0	3.76	0	2
Q99447	3.75	2.78	1
Q15599	3.748	2.05	1
Q9BW27	3.744	1.98	1
Q15843	3.744	2.38	1
Q9Y2Y0	3.742	1.88	1
Q9HC21	3.74	1.99	1
Q9P0U4	3.74	1.87	1
Q14728	3.737	2.85	1
Q9UIQ6	3.733	4.27	1
Q96CN9	3.726	2.3	1
Q96QA5	3.726	0	1
Q7Z5K2	3.724	2.02	1
Q6ICG6	3.721	0	1
Q15208	3.721	0	1
Q8IWT0	3.707	2.57	1
Q96T23	3.703	0	1
O95363	3.701	2.16	1
O75044	3.7	1.71	1
Q8IUD2	3.699	2.19	1
O00268	3.69	0	1
O75683	3.688	2.1	2
Q96HY7	3.683	3.72	2
O00217	3.679	2.25	1
Q7L5N7	3.673	0	1
Q9NUP1	3.669	0	1
[illegible]	[illegible]	[illegible]	[illegible]

Q9NX24	3.663	1.72	1
O75600	3.658	2.64	1
Q9BYC9	3.658	1.95	2
Q96D71	3.656	2.35	1
Q5VZL5	3.655	0	1
Q96GW9	3.655	0	1
CON_P02668	3.653	2.24	1
Q9H501	3.646	1.94	1
Q96HW7	3.642	1.78	1
Q6P1R4	3.636	0	1
O95299	3.636	4.06	1
Q96BJ3	3.634	0	1
O75529	3.633	0	1
O60783	3.632	2.97	1
Q9NPL8	3.631	2	1
Q12824	3.626	2.66	1
Q14574	3.625	2.49	1
Q9UHB6	3.624	1.79	1
Q9Y496	3.624	3.79	1
Q96T17	3.619	2.7	1
Q9H7X3	3.619	0	1
Q8NDF8	3.616	1.81	1
Q8WVV9	3.615	1.99	1
P08651	3.605	2.49	2
Q96HU8	3.599	4.15	1
Q6PL24	3.598	1.78	1
Q06136	3.592	0	1
Q5VZE5	3.589	2.09	1
P31146	3.581	1.77	1
Q9Y2Z2	3.573	2.39	1
O14773	3.572	2.07	1
Q8TDP1	3.571	0	1
Q96CB9	3.57	3.75	2
O75127	3.562	0	1
P62341	3.553	3.83	1
P42694	3.552	2.18	2
P06732	3.546	10.39	1
Q6VN20	3.543	2.62	1
P23610	3.539	1.78	1
O00483	3.535	1.88	1
Q8N0X7	3.528	0	1
Q96EB1	3.523	2.36	1
Q6P587	3.522	0	2
Q96BR5	3.522	4.26	1
Q16637	3.522	2.37	1
Q96PV6	3.519	2.14	1
Q9HCG8	3.517	2.21	2
[illegible]	[illegible]	[illegible]	[illegible]

Q5FBB7	3.504	1.89	1
Q9Y4E1	3.503	0	1
O14531	3.502	0	2
Q96EE3	3.502	0	1
Q9HBI1	3.495	1.74	1
Q96ME7	3.493	2.02	1
P82664	3.49	2.17	1
CON_Q3T052	3.49	0	1
P46736	3.489	0	2
Q9BZD4	3.486	2.01	1
Q03111	3.483	0	1
Q9BV81	3.481	4.8	1
O95677	3.479	0	2
Q8N9N8	3.477	2.25	1
Q96QD9	3.476	1.91	1
Q9H7C9	3.475	2.4	1
P18074	3.47	0	1
Q6Y1H2	3.468	1.7	1
Q9UDX5	3.465	2.18	1
Q9NZJ4	3.464	2.26	1
Q96JM3	3.461	1.71	1
CON_Q2UVX4	3.457	1.94	1
A6NGN4	3.455	2.31	1
P49754	3.455	1.78	1
P36402	3.452	0	1
O15075	3.445	1.73	1
Q9NUW8	3.439	0	1
O94887	3.438	0	1
Q86WQ0	3.438	0	1
Q5JTJ3	3.433	2.52	1
Q9H329	3.431	2.44	1
O43396	3.428	1.64	2
P27105	3.426	0	1
Q9Y5Y5	3.424	2.05	1
P52657	3.423	2.01	1
Q9Y3L3	3.418	1.81	1
Q9UPQ3	3.416	2.11	1
Q9BQE4	3.412	2.73	1
P09132	3.41	0	1
Q9NXV2	3.402	3.75	1
Q15526	3.398	0	1
O75438	3.392	1.7	1
Q16740	3.392	1.98	1
Q08188	3.389	2.12	1
Q9BTY2	3.387	0	1
Q9UHW5	3.386	1.71	1
Q8WVD3	3.385	1.83	1
[illegible]	[illegible]	[illegible]	[illegible]

Q14657	3.383	2.4	1
Q9BYG5	3.37	1.7	1
P18615	3.364	0	1
Q9BQC3	3.362	0	1
Q9NV31	3.351	2.59	1
P50416	3.349	1.76	1
Q6IAN0	3.348	0	1
Q12948	3.348	2.08	1
Q8IV48	3.347	1.86	1
O95881	3.343	3.29	1
Q9NWU2	3.342	0	1
Q03154	3.341	0	1
Q13613	3.341	2.2	1
Q3ZCW2	3.336	0	1
Q9BSH5	3.335	0	2
Q13309	3.321	0	1
Q96B36	3.321	2.18	1
Q9BWH6	3.32	2.25	1
O96033	3.315	2.25	1
Q8N0U8	3.315	0	2
Q96IV0	3.31	2.04	1
Q6Y288	3.308	0	1
Q96A26	3.308	4.14	2
Q9P2X0	3.305	1.73	1
Q9Y296	3.303	1.68	2
Q86VM9	3.301	1.77	1
Q96CW5	3.3	1.81	1
P61601	3.297	2.27	1
Q9BSF4	3.295	0	1
Q13501	3.291	0	1
Q15904	3.291	2.2	1
O94817	3.276	0	1
O15116	3.276	2.27	1
Q9Y5P4	3.272	0	2
CON_P17690	3.267	2.15	1
Q9NUM4	3.266	3.83	1
Q9NPF0	3.264	1.68	1
O60343	3.262	2.13	1
Q9HAU5	3.261	0	2
P11166	3.26	2.03	1
Q6RW13	3.257	2.7	1
O95297	3.252	4.62	1
Q86Y07	3.25	0	1
Q9UHA4	3.244	2.32	1
Q9BTU6	3.244	0	1
Q9NP81	3.241	2.16	1
O14653	3.24	0	1
[illegible]	[illegible]	[illegible]	[illegible]

Q9Y657	3.234	2.12	1
Q6UWF7	3.233	6.01	1
Q13948	3.229	1.64	1
Q0PNE2	3.229	1.63	1
Q9UJ83	3.224	0	1
Q9Y287	3.223	2.14	1
Q8N5N7	3.217	1.73	1
Q15398	3.216	0	2
P40337	3.215	0	1
Q4LE39	3.214	0	1
Q8NEM2	3.212	1.88	1
P53582	3.204	1.81	1
Q14534	3.204	0	1
O15164	3.193	0	1
Q8NE86	3.184	0	2
Q9UGR2	3.183	1.93	1
Q5SGD2	3.181	1.63	1
P61970	3.179	2.42	1
Q13426	3.178	3.69	1
Q7Z6K5	3.177	2.42	1
Q13084	3.176	0	2
Q9BRL6	3.176	4.86	1
Q9Y217	3.173	0	1
Q9UJ41	3.168	1.82	1
Q5JU85	3.167	1.61	2
P51580	3.16	1.9	1
Q6P1N0	3.159	2.05	1
Q15276	3.158	1.68	1
A8MWY0	3.149	3.7	2
Q3SY69	3.148	1.71	1
P49356	3.145	2.25	1
Q7Z2K6	3.141	0	1
O00560	3.14	2.12	1
Q96BP2	3.136	2.42	1
Q96K58	3.132	2.12	1
Q5JVS0	3.128	2.09	1
P46108	3.126	1.6	2
O95140	3.125	1.66	1
O75947	3.124	2.38	1
Q9BWW4	3.122	0	1
Q86UA1	3.12	0	1
Q9BW83	3.118	1.87	1
Q9UNN8	3.114	2.18	1
Q6DKK2	3.114	0	1
Q6ZN55	3.107	0	1
Q8N4T8	3.105	0	1
Q9NWT8	3.103	0	1
Q6DD87	3.101	0	1

Q9HD20	3.1	1.82	1
P01040	3.098	0	1
P09455	3.097	0	2
P09884	3.097	0	1
Q9NP92	3.094	0	1
O76075	3.089	1.72	1
Q96EC8	3.086	1.71	1
Q9BXB4	3.084	1.61	1
Q969Y2	3.081	1.64	1
Q9NWX6	3.079	2.28	1
Q96AQ6	3.066	1.79	1
Q9P2N5	3.063	0	2
Q9HCU5	3.06	3.92	1
P11117	3.057	0	1
O95503	3.048	0	1
P35610	3.048	2.12	1
Q9NR46	3.046	0	1
Q9BTX1	3.039	1.83	1
P61457	3.039	0	1
Q9UGJ1	3.03	2.39	1
Q14145	3.026	2.65	1
Q8IYI6	3.02	2.33	1
Q9BUL5	3.01	1.9	1
Q2TAA5	2.999	0	1
Q9NYR9	2.99	2.21	1
O15318	2.989	0	1
Q9H2P0	2.985	2.38	1
P30260	2.985	1.66	1
P61803	2.978	2.27	1
Q9HAS0	2.966	0	2
Q9NY12	2.962	3.6	1
Q9BXR0	2.961	6.67	1
P22681	2.953	1.64	1
Q9BRD0	2.949	2.16	1
O14981	2.944	0	1
Q3SXM5	2.94	2.38	1
Q8N5I2	2.939	2.54	1
Q9BXW6	2.937	2.21	1
Q8N573	2.936	0	1
O75530	2.928	0	1
Q9GZS3	2.922	0	1
P83111	2.92	0	1
Q96S55	2.919	0	1
Q6YHU6	2.914	0	1
Q5T8D3	2.913	0	2
Q6PJI9	2.912	0	1
Q8IZV5	2.912	0	2
[illegible]	[illegible]	[illegible]	[illegible]

Q8WY22	2.905	2.67	1
O95707	2.902	4.2	1
Q8N4H5	2.901	0	1
Q13555	2.898	0	1
Q8N3X1	2.882	1.88	1
Q6ZXV5	2.88	0	1
O43583	2.878	0	1
O75063	2.878	1.89	1
Q15517	2.875	0	1
O14524	2.868	2.04	1
P17931	2.863	0	1
P25490	2.861	2.12	1
Q8NAF0	2.859	0	1
Q96I15	2.856	0	1
Q13888	2.85	2.59	1
P49759	2.848	0	1
Q14318	2.844	2.58	1
Q9NUT2	2.841	0	1
Q15828	2.838	0	1
Q8N3E9	2.837	0	1
Q9Y2J2	2.83	0	2
Q9NRS6	2.819	1.82	1
Q9UK45	2.814	4.22	1
Q8IXJ6	2.813	1.67	1
Q6P161	2.813	1.75	1
Q9GZU8	2.809	0	1
Q6NW34	2.806	1.66	1
Q6UWP7	2.805	1.6	1
P51946	2.797	2.68	1
O14757	2.788	0	1
Q9Y5A7	2.787	0	1
P23434	2.787	0	1
P38935	2.786	1.71	1
Q9BTE6	2.783	1.77	1
Q9UHN1	2.778	0	1
Q16775	2.765	0	1
Q96NY9	2.765	0	1
O95391	2.761	0	1
Q8N8N7	2.752	1.84	1
Q8TF50	2.749	3.66	1
O95352	2.748	0	1
Q9P0J1	2.736	0	1
Q96EA4	2.73	1.89	1
Q9NX76	2.729	1.68	1
P22830	2.729	0	1
P82675	2.725	1.8	1
Q5T2E6	2.721	1.99	1
[illegible]	[illegible]	[illegible]	[illegible]

Q14781	2.714	1.64	1
Q9H4I3	2.71	1.78	1
Q9H1D9	2.71	1.63	1
Q9Y639	2.703	0	1
Q7L7X3	2.702	2.19	1
O60551	2.7	2.14	1
Q9UM47	2.698	1.67	1
Q8WZA9	2.697	2.24	1
Q96RR1	2.694	2.14	1
Q5VW38	2.69	0	1
Q9NWB6	2.68	2.34	1
O75525	2.679	3.77	1
Q9NRG7	2.678	0	1
Q9NY27	2.673	0	1
Q15054	2.67	0	1
P08236	2.668	1.71	1
O76071	2.667	0	1
Q9UNT1	2.666	0	1
Q15120	2.659	0	2
P01859	2.655	1.7	1
Q8N0Z6	2.65	2.33	1
Q96C00	2.648	0	1
Q8N1F8	2.646	0	1
Q5XKP0	2.645	3.27	2
O75820	2.642	2.12	1
O14683	2.638	0	1
P07108	2.636	1.64	1
P42345	2.63	0	1
Q9UBT7	2.628	2.11	1
Q8N2K0	2.625	1.98	1
Q9Y2V7	2.624	0	1
O43318	2.621	0	1
P17706	2.621	1.89	1
Q8WVT3	2.619	0	1
O94855	2.617	0	1
Q9NQ50	2.612	0	1
Q6PJ69	2.61	1.7	1
P50897	2.609	0	1
Q8WTV0	2.609	1.62	1
Q9Y3B2	2.605	2.21	1
Q14790	2.603	0	1
Q5BJF2	2.588	0	1
P45985	2.587	0	1
Q16774	2.587	0	1
Q92643	2.585	1.78	1
P09110	2.585	2.6	1
Q9UBZ4	2.575	0	1
[illegible]	[illegible]	[illegible]	[illegible]

P35626	2.569	1.66	1
Q05397	2.568	0	1
O94830	2.567	0	1
Q9UHQ4	2.567	0	1
Q8N0V3	2.566	0	1
Q9H900	2.56	1.89	1
Q9BPY3	2.558	0	1
Q96G74	2.555	0	1
Q12959	2.555	1.64	1
Q9Y2H5	2.551	0	1
Q9UQN3	2.551	0	1
Q8N5M1	2.55	0	1
P85298	2.543	0	1
Q86WA8	2.542	1.86	1
Q8IU60	2.538	0	1
Q9NVM9	2.538	0	2
Q9UDW1	2.537	0	1
Q4KMP7	2.535	0	1
Q86TU7	2.534	0	1
P78383	2.531	0	1
Q6ZUX7	2.53	0	1
Q9NW08	2.527	1.98	1
Q5HYK3	2.514	1.71	1
Q12947	2.513	0	1
Q15773	2.511	2.17	1
O94760	2.511	0	1
Q86V88	2.506	1.83	1
O95071	2.506	1.99	1
Q4KWH8	2.505	2	1
Q9NW68	2.5	0	1
Q96IS3	2.496	1.67	1
P85299	2.489	0	1
P54105	2.489	1.94	1
Q9Y248	2.463	0	1
Q8NHV4	2.461	0	1
P16220	2.46	1.96	1
P00156	2.456	0	1
Q99584	2.453	0	1
P15954	2.452	0	1
Q9BW61	2.448	0	1
Q86UK7	2.443	0	1
Q9UIJ7	2.439	1.65	1
Q86V85	2.435	2.05	1
Q96AE7	2.427	0	1
P02792	2.417	1.9	1
Q8TB61	2.414	0	1
Q16595	2.408	0	1
[illegible]	[illegible]	[illegible]	[illegible]

Q8NFI3	2.398	0	1
Q6DCA0	2.397	0	1
Q12965	2.388	1.61	1
Q7RTV0	2.387	1.87	1
P02671	2.384	2.14	1
Q6NW29	2.377	0	1
Q6VEQ5	2.374	1.98	1
O75027	2.372	0	1
Q96JK2	2.366	0	1
Q69YN4	2.365	0	2
Q15814	2.362	0	1
O15541	2.357	2.26	1
Q8IZD4	2.356	1.91	1
CON_ENSEMBL:ENSBTAP00000018574	2.354	0	1
Q15125	2.352	3.8	1
Q5THJ4	2.344	0	1
Q9ULK4	2.342	0	1
P01857	2.338	1.8	1
Q92692	2.336	1.91	1
P11498	2.329	0	1
Q9P016	2.328	1.87	1
Q86WB0	2.324	0	1
Q8NCG7	2.318	2.01	1
P69905	2.315	0	1
P20020	2.31	0	1
Q8IYS1	2.308	0	1
Q9HBM6	2.306	1.78	1
Q8NBQ5	2.306	0	1
Q92665	2.301	0	1
Q9BRT9	2.299	0	1
A0A075B6R9	2.294	3.9	1
Q9H6E4	2.293	0	1
P54802	2.289	0	1
O00746	2.289	0	1
Q86X83	2.281	1.87	1
Q9Y366	2.28	0	1
Q96BK5	2.275	0	1
Q9NP72	2.254	0	1
Q6PK04	2.25	0	1
P60201	2.246	1.7	1
Q9BSR8	2.238	2.41	1
Q8WUM0	2.228	0	1
P62273	2.226	1.85	1
Q9NW07	2.223	0	1
Q96BN8	2.22	0	1
P42898	2.217	0	1
Q9P2K8	2.215	1.78	1
[illegible]	[illegible]	[illegible]	[illegible]

Q9HBE1	2.207	0	1
Q9H4A6	2.198	0	1
Q8WWI5	2.198	0	1
Q5T4S7	2.181	0	1
Q13144	2.174	0	1
Q9Y5U9	2.172	1.96	1
CON_Q3MHN5	2.169	0	1
Q8WZA0	2.169	0	1
Q05086	2.161	1.64	1
P62487	2.158	1.82	1
Q9UNS1	2.158	1.8	1
Q9NX02	2.156	0	1
Q9Y316	2.156	1.65	1
Q16134	2.155	0	1
Q9UJX3	2.154	0	1
Q96SL8	2.153	0	1
O43293	2.152	0	1
Q9NP66	2.151	0	1
Q9Y3P9	2.146	0	1
Q92558	2.145	0	1
Q9Y2X0	2.145	1.62	1
Q9H8Y5	2.142	1.62	1
P06400	2.14	0	1
Q13017	2.138	0	1
P53609	2.137	2.24	1
O95251	2.137	0	1
Q96NX9	2.13	2	1
Q9P2X3	2.122	0	1
P49848	2.116	0	2
Q8N2H4	2.114	0	1
O43347	2.106	0	1
Q96ER3	2.104	0	1
Q8TAE8	2.104	1.61	1
Q04446	2.101	2.02	1
Q9ULH0	2.097	1.81	1
O75147	2.096	0	1
P35226	2.095	0	1
Q96SB3	2.084	0	1
O95684	2.084	1.82	1
Q6PJW8	2.082	1.81	1
O95630	2.076	0	1
O75934	2.075	0	1
Q9H7L9	2.071	0	1
O14657	2.07	0	1
O60486	2.065	1.82	1
O00115	2.063	0	1
O60566	2.06	0	1
[illegible]	[illegible]	[illegible]	[illegible]

P83369	2.044	1.63	1
Q86TN4	2.043	0	1
Q9BT40	2.039	0	1
Q9BZH6	2.038	0	1
O14777	2.037	0	1
Q16656	2.036	0	1
Q8NDV7	2.035	1.96	1
O60870	2.035	0	1
O14965	2.027	0	1
P49366	2.025	0	1
Q08426	2.025	0	1
O95900	2.021	1.9	1
Q9Y597	2.021	1.73	1
O43598	2.017	0	1
Q5TA45	2.012	0	1
P37287	2.007	0	1
P02458	2.005	0	1
Q96E29	1.991	1.91	1
Q92581	1.981	1.79	1
O75330	1.981	0	1
P31321	1.978	0	1
Q3B7T1	1.976	0	1
Q6NTE8	1.971	0	1
Q6PIU2	1.97	0	1
Q96NB2	1.967	0	1
Q9UI43	1.963	0	1
O43427	1.958	0	1
B9A064	1.954	0	1
Q9P265	1.951	0	1
Q13901	1.95	0	1
A0A075B6S5	1.947	0	1
Q9BRT6	1.945	2.07	1
Q16512	1.935	1.71	1
P49760	1.924	0	1
O14893	1.922	1.79	1
Q9H5Z1	1.922	0	1
Q12888	1.922	1.61	1
Q9Y3S2	1.918	0	1
Q15154	1.916	2.06	1
Q86YQ8	1.912	0	1
Q5CZC0	1.909	0	1
Q8WVJ2	1.901	5.06	1
Q96C57	1.901	0	1
Q7L590	1.9	0	1
Q8IWT3	1.898	0	1
Q8IZQ5	1.888	0	1
Q9Y4F4	1.888	2.04	1
[illegible]	[illegible]	[illegible]	[illegible]

Q6P3X3	1.886	0	1
Q9UGM6	1.885	0	1
P34947	1.882	0	1
P51688	1.879	0	1
Q96DM3	1.874	0	1
Q9Y5X1	1.87	0	1
O14802	1.861	0	1
O94762	1.857	0	1
O00443	1.857	0	1
Q9NS86	1.855	0	1
Q9NRL2	1.852	0	1
Q9UL41	1.842	1.68	1
Q86XL3	1.841	0	1
O75452	1.841	0	1
CON_ENSEMBL:ENSBTAP00000024462	1.841	0	1
Q7Z628	1.84	0	1
Q9HCU9	1.838	0	1
Q96IZ0	1.838	1.65	1
Q99496	1.837	0	1
Q8WUY8	1.835	1.95	1
Q9H6V9	1.833	1.96	1
Q9BUE6	1.83	1.6	1
Q6P1A2	1.828	0	1
P00403	1.828	3.67	1
Q4KMQ2	1.824	0	1
Q9BVA0	1.823	0	1
Q8IYB7	1.814	0	1
Q86SZ2	1.805	0	1

Supplementary Table 5 Protein lactylated in human PDAC samples and KPC cell lines

Gene name	Human	Mouse
SMAP	YES	YES
LAD1	YES	YES
PSMA7	YES	YES
GIPC1	YES	YES
ARPC2	YES	YES
OGT	YES	YES
EIF3D	YES	YES
PHGDH	YES	YES
SART1	YES	YES
ENSA	YES	YES
PLIN3	YES	YES
SNX2	YES	YES
DKC1	YES	YES
EDF1	YES	YES
MACROH2A1	YES	YES
FLNB	YES	YES
NCOR1	YES	YES
SF3B1	YES	YES
RPS6KA4	YES	YES
EIF3G	YES	YES
RSL1D1	YES	YES
URI1	YES	YES
MTA2	YES	YES
PLPBP	YES	YES
ZRANB2	YES	YES
GLUD1	YES	YES
SOD1	YES	YES
GOT2	YES	YES
PGK1	YES	YES
LMNA	YES	YES
ALDOA	YES	YES
GAPDH	YES	YES
ALDH2	YES	YES
HMGN1	YES	YES
SLC25A5	YES	YES
HMGN2	YES	YES
RPLP2	YES	YES
SSB	YES	YES
PTMA	YES	YES
ATP5F1B	YES	YES
ENO1	YES	YES
NPM1	YES	YES
TPM3	YES	YES
ANXA2	YES	YES

HNRNPC	YES	YES
PFKM	YES	YES
HSP90AB1	YES	YES
VIM	YES	YES
ANXA5	YES	YES
RPSA	YES	YES
SNRPA	YES	YES
LGALS1	YES	YES
HMGB1	YES	YES
RBP1	YES	YES
DLD	YES	YES
HNRNPA1	YES	YES
SNRPA1	YES	YES
PARP1	YES	YES
CALM3	YES	YES
H1-4	YES	YES
HSPD1	YES	YES
HSPA5	YES	YES
HSPA8	YES	YES
PABPC1	YES	YES
SLC25A4	YES	YES
XRCC5	YES	YES
EEF2	YES	YES
PLS3	YES	YES
AKR1A1	YES	YES
PKM	YES	YES
HNRNPL	YES	YES
EZR	YES	YES
RPS2	YES	YES
H2AX	YES	YES
H1-5	YES	YES
H1-2	YES	YES
HMGA1	YES	YES
PFKL	YES	YES
LGALS3	YES	YES
RPL7	YES	YES
VCL	YES	YES
NCL	YES	YES
EIF2S2	YES	YES
CAST	YES	YES
PTMS	YES	YES
CSRP1	YES	YES
FLNA	YES	YES
SFPQ	YES	YES
RPS3	YES	YES
CFL1	YES	YES
EIF4B	YES	YES
ACAT1	YES	YES

RPS12	YES	YES
ATP5F1A	YES	YES
PSMA1	YES	YES
PSMA3	YES	YES
MSN	YES	YES
HMGB2	YES	YES
EEF1G	YES	YES
RPA1	YES	YES
APEX1	YES	YES
CALR	YES	YES
MAP4	YES	YES
CANX	YES	YES
TKT	YES	YES
EEF1D	YES	YES
RPL12	YES	YES
SDHA	YES	YES
STIP1	YES	YES
S100A11	YES	YES
H2BC3	YES	YES
HSPA4	YES	YES
CTNNA1	YES	YES
GTF2F1	YES	YES
MYH9	YES	YES
FUS	YES	YES
ATP5F1C	YES	YES
RPL4	YES	YES
TAGLN2	YES	YES
TALDO1	YES	YES
HSPA9	YES	YES
EIF4A3	YES	YES
RPS19	YES	YES
CCT6A	YES	YES
MDH2	YES	YES
HADHA	YES	YES
GARS1	YES	YES
TMPO	YES	YES
ACAA2	YES	YES
RPL35	YES	YES
MATR3	YES	YES
CBX5	YES	YES
NSF	YES	YES
RPL27A	YES	YES
RPL5	YES	YES
RPL28	YES	YES
RPS9	YES	YES
RPS10	YES	YES
IQGAP1	YES	YES
CAPZA2	YES	YES

RPL29	YES	YES
ATP5PO	YES	YES
IDH2	YES	YES
RPL34	YES	YES
TUFM	YES	YES
PCYT1A	YES	YES
ACADVL	YES	YES
HINT1	YES	YES
RANBP2	YES	YES
GATM	YES	YES
RPL14	YES	YES
MECP2	YES	YES
HCFC1	YES	YES
HDGF	YES	YES
UBE2E1	YES	YES
HNRNPM	YES	YES
HMGA2	YES	YES
CRIP2	YES	YES
NUP98	YES	YES
ACLY	YES	YES
SUCLG1	YES	YES
SUB1	YES	YES
YARS1	YES	YES
EIF5	YES	YES
VCP	YES	YES
ADK	YES	YES
EEFSEC	YES	YES
EIF4A1	YES	YES
RPS20	YES	YES
S100A10	YES	YES
DSTN	YES	YES
ACTR2	YES	YES
ABCE1	YES	YES
RPS3A	YES	YES
HSPE1	YES	YES
SUMO2	YES	YES
HNRNPK	YES	YES
PPP1CA	YES	YES
PPP1CB	YES	YES
RPS16	YES	YES
RPS11	YES	YES
TMSB4X	YES	YES
RPL7A	YES	YES
RPL23A	YES	YES
RPS6	YES	YES
RAN	YES	YES
RPS24	YES	YES
RPS25	YES	YES

RPS28	YES	YES
RPL31	YES	YES
PPIA	YES	YES
RPS27A	YES	YES
YWHAZ	YES	YES
RPS21	YES	YES
EIF5A	YES	YES
EEF1A1	YES	YES
GTF2I	YES	YES
GSTO1	YES	YES
SARNP	YES	YES
RPL24	YES	YES
RPL19	YES	YES
SLC25A3	YES	YES
HDLBP	YES	YES
FKBP3	YES	YES
HNRNPU	YES	YES
SPTBN1	YES	YES
CAP1	YES	YES
PFKP	YES	YES
RPL18A	YES	YES
TOP2B	YES	YES
PRDX1	YES	YES
DHX9	YES	YES
TP53BP1	YES	YES
ILF3	YES	YES
IK	YES	YES
CBX3	YES	YES
UBE2V1	YES	YES
NNT	YES	YES
TCOF1	YES	YES
PDAP1	YES	YES
HDAC1	YES	YES
CUL3	YES	YES
COTL1	YES	YES
HNRNPD	YES	YES
DPYSL3	YES	YES
CTTN	YES	YES
TRIM25	YES	YES
CHD4	YES	YES
LASP1	YES	YES
NOLC1	YES	YES
NUMA1	YES	YES
EIF4H	YES	YES
EEA1	YES	YES
PLEC	YES	YES
PTGES3	YES	YES
NONO	YES	YES

PCBP2	YES	YES
CNN3	YES	YES
SAFB	YES	YES
SF3A1	YES	YES
MAPRE1	YES	YES
CSRP2	YES	YES
DPYSL2	YES	YES
H2BC21	YES	YES
HNRNPUL2	YES	YES
PDS5A	YES	YES
ACSF3	YES	YES
RIF1	YES	YES
ARL6IP4	YES	YES
CIAPIN1	YES	YES
HIBCH	YES	YES
PRPF8	YES	YES
H3C15	YES	YES
DDX46	YES	YES
CYFIP1	YES	YES
KDM3B	YES	YES
HDGFL2	YES	YES
ARPIN	YES	YES
GATAD2A	YES	YES
ARFGAP2	YES	YES
SERBP1	YES	YES
ABCF1	YES	YES
MCPH1	YES	YES
THOC2	YES	YES
PCNP	YES	YES
ZFPM2	YES	YES
PSPC1	YES	YES
BBX	YES	YES
DDX1	YES	YES
ZNF592	YES	YES
MYO18A	YES	YES
DDX17	YES	YES
KHSRP	YES	YES
LPP	YES	YES
LRRC59	YES	YES
PDLIM5	YES	YES
RBM17	YES	YES
PNKP	YES	YES
CNN2	YES	YES
PHB2	YES	YES
HSD17B10	YES	YES
HNRNPAB	YES	YES
NAP1L4	YES	YES
ACO2	YES	YES

CCT7	YES	YES
H2BC14	YES	YES
PYM1	YES	YES
UTP23	YES	YES
STK31	YES	YES
NUCKS1	YES	YES
CHMP4B	YES	YES
CACYBP	YES	YES
PDLIM7	YES	YES
DDX21	YES	YES
NANS	YES	YES
OLA1	YES	YES
SEPTIN11	YES	YES
THUMPD1	YES	YES
THYN1	YES	YES
VAPA	YES	YES
RCC2	YES	YES
RRBP1	YES	YES
LARS1	YES	YES
SUCLA2	YES	YES
SAE1	YES	YES
HDAC6	YES	YES
CFDP1	YES	YES
TES	YES	YES
LIMA1	YES	YES
SEPTIN9	YES	YES
DBNL	YES	YES
STOML2	YES	YES
JPT1	YES	YES
RALY	YES	YES
CORO1C	YES	YES
NSFL1C	YES	YES
CDYL	YES	YES
TMA7	YES	YES
THRAP3	YES	YES
WBP11	YES	YES
NOP58	YES	YES
TLN1	YES	YES
NCOR2	YES	YES
POTEF	YES	NO
WASH4P	YES	NO
PSMD9	YES	NO
CLIC1	YES	NO
HMGN4	YES	NO
NCKAP5	YES	NO
PRMT5	YES	NO
NDC80	YES	NO
BACH1	YES	NO

ARPC1B	YES	NO
PGRMC2	YES	NO
LAMA5	YES	NO
ARPC5	YES	NO
DENR	YES	NO
ACTN4	YES	NO
LANCL1	YES	NO
SSX5	YES	NO
CTNND1	YES	NO
USO1	YES	NO
DNAJA2	YES	NO
ABCB7	YES	NO
HIP1R	YES	NO
KDM4A	YES	NO
SNRNP200	YES	NO
IDH1	YES	NO
NEURL1	YES	NO
SRP72	YES	NO
MYO1D	YES	NO
ERLIN2	YES	NO
OXSR1	YES	NO
EML2	YES	NO
AGR2	YES	NO
GAS8	YES	NO
ADH1B	YES	NO
ADH1C	YES	NO
LDHA	YES	NO
ALDH1A1	YES	NO
CYB5R3	YES	NO
GSR	YES	NO
CP	YES	NO
F13A1	YES	NO
PNP	YES	NO
HP	YES	NO
CA1	YES	NO
SPINK1	YES	NO
SERPINA1	YES	NO
SERPINA3	YES	NO
C3	YES	NO
KNG1	YES	NO
IGHG2	YES	NO
IGHA1	YES	NO
HBD	YES	NO
COL1A1	YES	NO
COL3A1	YES	NO
APOA1	YES	NO
APOA2	YES	NO
FGA	YES	NO

FGG	YES	NO
APCS	YES	NO
FN1	YES	NO
AHSG	YES	NO
TF	YES	NO
LTF	YES	NO
HPX	YES	NO
PLA2G1B	YES	NO
FUCA1	YES	NO
CSTB	YES	NO
AMY2A	YES	NO
HSPB1	YES	NO
RPN1	YES	NO
ATP1A1	YES	NO
RPLP0	YES	NO
REG1A	YES	NO
KRT18	YES	NO
GSN	YES	NO
GPI	YES	NO
H2BC11	YES	NO
DBI	YES	NO
LDHB	YES	NO
P4HB	YES	NO
H1-0	YES	NO
CTSD	YES	NO
TUBB	YES	NO
PRSS2	YES	NO
DCN	YES	NO
EPRS1	YES	NO
CTSB	YES	NO
HSP90AA1	YES	NO
RET	YES	NO
THBS1	YES	NO
COL1A2	YES	NO
ANXA6	YES	NO
CELA2A	YES	NO
SRPRA	YES	NO
GSTA1	YES	NO
COL4A2	YES	NO
CELA3B	YES	NO
CELA3A	YES	NO
ANXA4	YES	NO
ALDOC	YES	NO
HSPA1B	YES	NO
IGLC3	YES	NO
SCHIP1	YES	NO
TXN	YES	NO
LAMC1	YES	NO

PYGB	YES	NO
RALA	YES	NO
ADH5	YES	NO
COL11A1	YES	NO
COL6A1	YES	NO
COL6A2	YES	NO
COL6A3	YES	NO
SLC25A6	YES	NO
F5	YES	NO
ACTN1	YES	NO
MYL4	YES	NO
XRCC6	YES	NO
RNH1	YES	NO
MT1G	YES	NO
PDIA4	YES	NO
TPT1	YES	NO
ALAD	YES	NO
LCP1	YES	NO
PRKAR2A	YES	NO
MIF	YES	NO
PRKCSH	YES	NO
HCLS1	YES	NO
HSP90B1	YES	NO
UQCRB	YES	NO
CPA1	YES	NO
CPB1	YES	NO
AKR1B1	YES	NO
GSPT1	YES	NO
PGAM2	YES	NO
ELN	YES	NO
NME1	YES	NO
CBR1	YES	NO
PNLIP	YES	NO
DES	YES	NO
RPL17	YES	NO
ITIH1	YES	NO
CEL	YES	NO
TYMP	YES	NO
ANXA7	YES	NO
LMNB1	YES	NO
OGN	YES	NO
BGN	YES	NO
TGM2	YES	NO
OSBP	YES	NO
UBA1	YES	NO
FBLN1	YES	NO
PPIB	YES	NO
SP100	YES	NO

H2BC17	YES	NO
CPT2	YES	NO
LAMA2	YES	NO
EEF1B2	YES	NO
TNC	YES	NO
VARS1	YES	NO
CRABP2	YES	NO
PML	YES	NO
MARCKS	YES	NO
PEBP1	YES	NO
PDIA3	YES	NO
PPIF	YES	NO
SRI	YES	NO
SERPINB1	YES	NO
AKT1	YES	NO
PRDX2	YES	NO
RPL9	YES	NO
LSP1	YES	NO
PRSS3	YES	NO
RPL22	YES	NO
THBS2	YES	NO
FBN1	YES	NO
MYH10	YES	NO
DEK	YES	NO
MYH11	YES	NO
GLRX	YES	NO
ARL3	YES	NO
PGM1	YES	NO
SERPINF1	YES	NO
DLST	YES	NO
CAPG	YES	NO
NNMT	YES	NO
MDH1	YES	NO
GRM5	YES	NO
STAT1	YES	NO
SSR2	YES	NO
RECQL	YES	NO
STT3A	YES	NO
CAPZB	YES	NO
CPA2	YES	NO
AARS1	YES	NO
GZMK	YES	NO
HNMT	YES	NO
SERPINH1	YES	NO
ALDH5A1	YES	NO
LUM	YES	NO
PRELP	YES	NO
CNN1	YES	NO

PGD	YES	NO
ARHGDIB	YES	NO
COPB1	YES	NO
COPA	YES	NO
HSPA2	YES	NO
BCAT1	YES	NO
EMP2	YES	NO
ADAR	YES	NO
LGALS4	YES	NO
H2BC5	YES	NO
ZNF280A	YES	NO
TPI1	YES	NO
MYL6	YES	NO
CDC42	YES	NO
UBE2N	YES	NO
RHOA	YES	NO
SEC61A1	YES	NO
RPS7	YES	NO
SNRPE	YES	NO
RHOB	YES	NO
H4C1	YES	NO
RAB1A	YES	NO
RPL23	YES	NO
RAP1A	YES	NO
RPS15	YES	NO
RPL32	YES	NO
RAC1	YES	NO
SKP1	YES	NO
RACK1	YES	NO
ACTG1	YES	NO
TPM4	YES	NO
ACTA1	YES	NO
TUBA1B	YES	NO
TUBB4B	YES	NO
PAFAH1B2	YES	NO
HBB	YES	NO
HBA1	YES	NO
PRKDC	YES	NO
MT1X	YES	NO
ABAT	YES	NO
LACTB	YES	NO
FBLN2	YES	NO
HSPG2	YES	NO
ATP2C1	YES	NO
CLTC	YES	NO
FABP5	YES	NO
ANK2	YES	NO
TAGLN	YES	NO

RPL6	YES	NO
LMNB2	YES	NO
CFHR1	YES	NO
COL10A1	YES	NO
CALD1	YES	NO
REG3A	YES	NO
RUNX1T1	YES	NO
RPL18	YES	NO
CKAP4	YES	NO
KLC1	YES	NO
CYP4F3	YES	NO
FGL1	YES	NO
AHNAK	YES	NO
SYCN	YES	NO
AIMP1	YES	NO
TRAP1	YES	NO
PDIA2	YES	NO
PABPC4	YES	NO
ASAH1	YES	NO
EIF4EBP1	YES	NO
DCTN2	YES	NO
NAE1	YES	NO
SPTAN1	YES	NO
SNTB1	YES	NO
DOCK1	YES	NO
DYNC1H1	YES	NO
SMC1A	YES	NO
MVP	YES	NO
GOLGB1	YES	NO
POSTN	YES	NO
PDIA6	YES	NO
PPA1	YES	NO
RSU1	YES	NO
RGN	YES	NO
TGFBI	YES	NO
TPSAB1	YES	NO
MYLK	YES	NO
ADIRF	YES	NO
SMN1	YES	NO
IFI16	YES	NO
ATP2B3	YES	NO
IMMT	YES	NO
ARHGAP44	YES	NO
INF2	YES	NO
CHD9	YES	NO
TKFC	YES	NO
ZNF568	YES	NO
CERKL	YES	NO

PDCD4	YES	NO
CCDC178	YES	NO
H2BC18	YES	NO
HP1BP3	YES	NO
SPOUT1	YES	NO
SWT1	YES	NO
H3-7	YES	NO
ANKRD20A3P	YES	NO
JAKMIP3	YES	NO
MAP1S	YES	NO
CTRB2	YES	NO
TWF2	YES	NO
PPP1R2B	YES	NO
OLFM4	YES	NO
CHRDL2	YES	NO
RASSF6	YES	NO
SND1	YES	NO
CHMP1B	YES	NO
TRPM1	YES	NO
KTN1	YES	NO
CAND1	YES	NO
WDR5B	YES	NO
IQCA1	YES	NO
AHNAK2	YES	NO
DNAH10	YES	NO
SLF2	YES	NO
SUPV3L1	YES	NO
AGGF1	YES	NO
ARL14	YES	NO
METTL25	YES	NO
CCNYL1	YES	NO
EFHB	YES	NO
BCORP1	YES	NO
UNC13C	YES	NO
MROH1	YES	NO
GIPC2	YES	NO
PPP1R13L	YES	NO
ATG4A	YES	NO
H1-10	YES	NO
NCSTN	YES	NO
SNX19	YES	NO
DHX38	YES	NO
PRCC	YES	NO
TFG	YES	NO
RAD50	YES	NO
PBXIP1	YES	NO
DCPS	YES	NO
NMD3	YES	NO

ERP27	YES	NO
DUS3L	YES	NO
PPP1R16A	YES	NO
BTF3L4	YES	NO
ZNF512B	YES	NO
RPL10L	YES	NO
MCEE	YES	NO
GBP5	YES	NO
VPS13A	YES	NO
CDK5RAP2	YES	NO
SEC62	YES	NO
PARK7	YES	NO
LGMN	YES	NO
COL12A1	YES	NO
H2BC15	YES	NO
H2AC14	YES	NO
H2BC13	YES	NO
GORASP1	YES	NO
CCDC77	YES	NO
TXNDC17	YES	NO
LXN	YES	NO
ASPN	YES	NO
TANC1	YES	NO
C20orf27	YES	NO
COMMD4	YES	NO
RAB17	YES	NO
C11orf54	YES	NO
SH3BGRL3	YES	NO
UNC45A	YES	NO
GOLPH3L	YES	NO
RAD21L1	YES	NO
RHOF	YES	NO
EML4	YES	NO
MUC5B	YES	NO
SMURF1	YES	NO
POLD4	YES	NO
OSTC	YES	NO
HEBP1	YES	NO
FARSB	YES	NO
ATG3	YES	NO
TMLHE	YES	NO
PNPO	YES	NO
UGGT1	YES	NO
FAM120A	YES	NO
TAAR2	YES	NO
DNAH2	YES	NO
SPRR3	YES	NO
DNAJB11	YES	NO

SRP68	YES	NO
ZNF608	YES	NO
MAGED2	YES	NO
C19orf53	YES	NO
ANKRD26	YES	NO
PA2G4	YES	NO
FNDC3A	YES	NO
ATP8A1	YES	NO
WASHC2C	YES	NO
HYOU1	YES	NO
CEP83	YES	NO
PAXBP1	YES	NO
SRPRB	YES	NO
TSSC4	YES	NO
PSAT1	YES	NO
ALG5	YES	NO
COPG1	YES	NO
Pdlim1	NO	YES
Cgnl1	NO	YES
Map1b	NO	YES
Zbtb43	NO	YES
Spen	NO	YES
Acp6	NO	YES
Fkbp1a	NO	YES
Wdr70	NO	YES
Eef1b	NO	YES
Rnf40	NO	YES
Cdv3	NO	YES
Rbm28	NO	YES
Znf800	NO	YES
Rbpj	NO	YES
Utp18	NO	YES
Psmc1	NO	YES
Dgcr8	NO	YES
Ccna2	NO	YES
Cops7a	NO	YES
Wipi2	NO	YES
Chmp2a	NO	YES
Ppwd1	NO	YES
Dap	NO	YES
Nck2	NO	YES
Atf1	NO	YES
Ptpn1	NO	YES
Map2k3	NO	YES
Sf1	NO	YES
Ing1	NO	YES
Phax	NO	YES
Ppp1cc	NO	YES

Hltf	NO	YES
Cbx8	NO	YES
Smarcad1	NO	YES
Mcm3	NO	YES
Prpf4	NO	YES
Senp6	NO	YES
Strn	NO	YES
Gart	NO	YES
Actb	NO	YES
Ints12	NO	YES
Ddx18	NO	YES
Cct8	NO	YES
Xab2	NO	YES
Srfbp1	NO	YES
Aspm	NO	YES
Lin37	NO	YES
Znf516	NO	YES
Arid5b	NO	YES
Nufip2	NO	YES
Gtf2b	NO	YES
Fam32a	NO	YES
Kpna6	NO	YES
Rprd2	NO	YES
Pold3	NO	YES
Pom121	NO	YES
Znf280d	NO	YES
Trim28	NO	YES
Baiap2l1	NO	YES
Rbm5	NO	YES
Ahcyl1	NO	YES
Cdk11b	NO	YES
Syf2	NO	YES
Arfgap1	NO	YES
Usp10	NO	YES
Sdhb	NO	YES
Knop1	NO	YES
Api5	NO	YES
Ncoa6	NO	YES
Smarca1	NO	YES
Gtf3c1	NO	YES
Pum3	NO	YES
Irak3	NO	YES
Sh3glb2	NO	YES
Usp42	NO	YES
Hells	NO	YES
Tpx2	NO	YES
Exoc4	NO	YES
Scyl1	NO	YES

Ehmt1	NO	YES
Smchd1	NO	YES
Kifc1	NO	YES
Rps4x	NO	YES
Ifi204	NO	YES
Prpf31	NO	YES
Cct3	NO	YES
Trir	NO	YES
Tdrd3	NO	YES
S100a4	NO	YES
Mad2l1	NO	YES
Ccdc9	NO	YES
Atp5if1	NO	YES
Lap3	NO	YES
Lsm12	NO	YES
Pclaf	NO	YES
Slc25a11	NO	YES
Nup133	NO	YES
Tead2	NO	YES
Zgrf1	NO	YES
Mphosph8	NO	YES
Gmps	NO	YES
Ruvbl2	NO	YES
Baz2b	NO	YES
Casp3	NO	YES
Ppp4r3a	NO	YES
Eps8	NO	YES
Utp20	NO	YES
Emsy	NO	YES
Zbtb7b	NO	YES
Pus7	NO	YES
Ccdc12	NO	YES
Cwc22	NO	YES
Wdr77	NO	YES
Sf3b6	NO	YES
Ddhd1	NO	YES
Dnm1l	NO	YES
Htatsf1	NO	YES
Parg	NO	YES
Hdac2	NO	YES
Rps5	NO	YES
Rtn4	NO	YES
Elp2	NO	YES
Pola1	NO	YES
Bysl	NO	YES
Dpy30	NO	YES
Bzw1	NO	YES
Stmn1	NO	YES

Slk	NO	YES
Tra2b	NO	YES
Ywhae	NO	YES
Ctbp2	NO	YES
Irf2bp2	NO	YES
Psme3	NO	YES
Rpl30	NO	YES
Ugdh	NO	YES
Mix23	NO	YES
Dnttip1	NO	YES
Snrpg	NO	YES
Kpnb1	NO	YES
Mrgbp	NO	YES
Eloa	NO	YES
Racgap1	NO	YES
Phlda2	NO	YES
Mfap1b	NO	YES
Noc3l	NO	YES
Srrm1	NO	YES
Hnrnpf	NO	YES
Tle3	NO	YES
Abitram	NO	YES
Hbs1l	NO	YES
Dcaf5	NO	YES
Rcor3	NO	YES
Ppp1r8	NO	YES
Smarca4	NO	YES
Asz1	NO	YES
Rrp8	NO	YES
Rabl6	NO	YES
Setd5	NO	YES
Spin1	NO	YES
Kif23	NO	YES
Ncapd2	NO	YES
Acot9	NO	YES
Tardbp	NO	YES
Gen1	NO	YES
Nfya	NO	YES
Dnajb6	NO	YES
Utp14a	NO	YES
Katnal1	NO	YES
Prr12	NO	YES
Aacs	NO	YES
Krr1	NO	YES
Ylpm1	NO	YES
Ebna1bp2	NO	YES
Nat10	NO	YES
Cdk5rap1	NO	YES

Sra1	NO	YES
Cbl	NO	YES
Rpl26	NO	YES
Sgo1	NO	YES
Gpatch4	NO	YES
Plaa	NO	YES
Kif14	NO	YES
Git2	NO	YES
Ppp4r2	NO	YES
Brms1l	NO	YES
Cycs	NO	YES
Rps14	NO	YES
Mepce	NO	YES
Tcf3	NO	YES
Ppp1r11	NO	YES
Camk2g	NO	YES
Rbm27	NO	YES
Baz2a	NO	YES
Cep152	NO	YES
Slc7a6os	NO	YES
Pcgf1	NO	YES
Rfc4	NO	YES
Ado	NO	YES
Ebag9	NO	YES
Foxk2	NO	YES
Rbbp7	NO	YES
Larp7	NO	YES
Dst	NO	YES
Septin2	NO	YES
Cep55	NO	YES
Ints4	NO	YES
Pabpn1l	NO	YES
Trmt6	NO	YES
Trmt10a	NO	YES
Lyar	NO	YES
Eif3j1	NO	YES
Cpsf1	NO	YES
Zfand1	NO	YES
Pcmt1	NO	YES
Wdr76	NO	YES
Znf219	NO	YES
Hmgn5	NO	YES
Rnaseh2b	NO	YES
Men1	NO	YES
Kpna2	NO	YES
Rpl3	NO	YES
Atox1	NO	YES
Phip	NO	YES

Mga	NO	YES
Sorbs2	NO	YES
Ints1	NO	YES
Pla2g4a	NO	YES
Kpna4	NO	YES
Nudt21	NO	YES
Daxx	NO	YES
Gclm	NO	YES
Snw1	NO	YES
H2bu1	NO	YES
Mtrex	NO	YES
Fip1l1	NO	YES
Mphosph6	NO	YES
Nelfe	NO	YES
Mybpc3	NO	YES
Morc3	NO	YES
Tsr1	NO	YES
Sap30bp	NO	YES
Smc2	NO	YES
Rbm39	NO	YES
Snx9	NO	YES
Lig3	NO	YES
Acsf2	NO	YES
Tk1	NO	YES
Rbm25	NO	YES
Rpap1	NO	YES
Wdr75	NO	YES
Dtd1	NO	YES
St13	NO	YES
Iars2	NO	YES
Brd4	NO	YES
Bicra	NO	YES
Rfxap	NO	YES
Ubtf	NO	YES
Bche	NO	YES
Nudt5	NO	YES
Kif2c	NO	YES
Nipbl	NO	YES
Nt5e	NO	YES
Incenp	NO	YES
Uba52	NO	YES
Sh2d4a	NO	YES
Cul5	NO	YES
Rbm22	NO	YES
Smarca5	NO	YES
Nopchap1	NO	YES
Plk1	NO	YES
Thoc5	NO	YES

H2bc8	NO	YES
Fyb1	NO	YES
Kif20a	NO	YES
Trrap	NO	YES
Prkrip1	NO	YES
Aak1	NO	YES
Ap2a2	NO	YES
Ahsa2	NO	YES
Tasor2	NO	YES
Uba2	NO	YES
Yap1	NO	YES
Gigyf2	NO	YES
Rreb1	NO	YES
Sec16a	NO	YES
Sympk	NO	YES
Nup50	NO	YES
Bod1l	NO	YES
Shoc2	NO	YES
Coil	NO	YES
Triobp	NO	YES
Rpl35a	NO	YES
Csde1	NO	YES
Acp1	NO	YES
Pak2	NO	YES
Eif4g1	NO	YES
Pnn	NO	YES
Wapl	NO	YES
Carmil1	NO	YES
Gigyf1	NO	YES
Ddx31	NO	YES
Suclg2	NO	YES
Epb41	NO	YES
Mcm5	NO	YES
Gatad2b	NO	YES
Sars1	NO	YES
Trnau1ap	NO	YES
Vapb	NO	YES
Puf60	NO	YES
Dnajc17	NO	YES
Gpatch8	NO	YES
Irf2	NO	YES
U2surp	NO	YES
Mki67	NO	YES
Nkapl	NO	YES
Plcl2	NO	YES
Gnl2	NO	YES
Snf8	NO	YES
Znf207	NO	YES

Ddx52	NO	YES
Slc27a4	NO	YES
H2ac20	NO	YES
Cnot6l	NO	YES
Uhrf1	NO	YES
Senp3	NO	YES
Bag2	NO	YES
Khdrbs1	NO	YES
Bend3	NO	YES
Suds3	NO	YES
Ghitm	NO	YES
Camk2d	NO	YES
Dsn1	NO	YES
Slc25a24	NO	YES
Flywch2	NO	YES
Nfia	NO	YES
Wdhd1	NO	YES
Wac	NO	YES
Micall2	NO	YES
Chmp1b2	NO	YES
Aif1l	NO	YES
Dnajc8	NO	YES
Fen1	NO	YES
Hmg20b	NO	YES
H1-1	NO	YES
Maoa	NO	YES
Paat	NO	YES
Sfr1	NO	YES
Nol4l	NO	YES
Hmgb3	NO	YES
Slain2	NO	YES
Prc1	NO	YES
Ubap2l	NO	YES
Qrich1	NO	YES
Cnot3	NO	YES
Sin3b	NO	YES
Arhgef2	NO	YES
Knl1	NO	YES
Afdn	NO	YES
Prdx6	NO	YES
Atm	NO	YES
Mark2	NO	YES
Kif4	NO	YES
Nelfa	NO	YES
Qser1	NO	YES
Lage3	NO	YES
Pqbp1	NO	YES
Hpf1	NO	YES

Efhd2	NO	YES
Raver1	NO	YES
Ppip5k2	NO	YES
Rrs1	NO	YES
Mtdh	NO	YES
Dcun1d5	NO	YES
Cstf3	NO	YES
Rbbp6	NO	YES
H4f16	NO	YES
Vasp	NO	YES
Atp5pf	NO	YES
Eppk1	NO	YES
Wdr45b	NO	YES
Ech1	NO	YES
Akap2	NO	YES
Mybbp1a	NO	YES
Ankrd11	NO	YES
Fam83g	NO	YES
Shtn1	NO	YES
Gpalpp1	NO	YES
Rad51ap1	NO	YES
Gmfb	NO	YES
Pus10	NO	YES
Cobll1	NO	YES
Gmip	NO	YES
Csnk2b	NO	YES
Rpl13a	NO	YES
Surf2	NO	YES
Cux1	NO	YES
Rps13	NO	YES
Aktip	NO	YES
Poldip3	NO	YES
Top1	NO	YES
Hsph1	NO	YES
Arpp19	NO	YES
Chd1l	NO	YES
Cdk7	NO	YES
Inip	NO	YES
Tpd52l1	NO	YES
Sh3pxd2b	NO	YES
Phf10	NO	YES
Scaf1	NO	YES
Rpl27	NO	YES
Phactr4	NO	YES
Arf6	NO	YES
Wdr43	NO	YES
Topbp1	NO	YES
Eif5b	NO	YES

Brd3	NO	YES
Phf6	NO	YES
Rnmt	NO	YES
Alkbh5	NO	YES
Pdcd5	NO	YES
Ogdh	NO	YES
Nvl	NO	YES
Mbd3	NO	YES
Tpd52	NO	YES
Dr1	NO	YES
Prpf40a	NO	YES
Znf687	NO	YES
Dcaf1	NO	YES
Arl6ip5	NO	YES
Gmnn	NO	YES
Chtf18	NO	YES
Ncbp3	NO	YES
Rcor2	NO	YES
Morc2a	NO	YES
Dhx36	NO	YES
Tbce	NO	YES
Ino80d	NO	YES
Mtatp8	NO	YES
Prpf3	NO	YES
Nme2	NO	YES
Znf830	NO	YES
Pogz	NO	YES
Mkrn2	NO	YES
Mcrip2	NO	YES
Irf2bp1	NO	YES
Clint1	NO	YES
Trip12	NO	YES
Sde2	NO	YES
Cggbp1	NO	YES
rp9	NO	YES
Rtf1	NO	YES
Nudc	NO	YES
Crat	NO	YES
Timeless	NO	YES
Frg1	NO	YES
Eif2a	NO	YES
Shmt2	NO	YES
Oga	NO	YES
Jmjd1c	NO	YES
Akr1e2	NO	YES
H2aj	NO	YES
Ubxn1	NO	YES
Wrnip1	NO	YES

Pak1ip1	NO	YES
Rab3ip	NO	YES
Nepro	NO	YES
Kdm1b	NO	YES
Xrcc1	NO	YES
Sap30	NO	YES
Paf1	NO	YES
Usp4	NO	YES
Agfg1	NO	YES
Creb1	NO	YES
Dcp2	NO	YES
Obi1	NO	YES
Nemf	NO	YES
Ints10	NO	YES
Gemin5	NO	YES
Ptbp1	NO	YES
Diaph1	NO	YES
Nedd1	NO	YES
Zfr	NO	YES
Kif11	NO	YES
Fam207a	NO	YES
Rpl38	NO	YES
Rbm14	NO	YES
Cdkn2aip	NO	YES
Fam107b	NO	YES
Ube2v2	NO	YES
Nop16	NO	YES
Aqr	NO	YES
Hmbs	NO	YES
H2az2	NO	YES
Impa2	NO	YES
Chmp5	NO	YES
Pes1	NO	YES
Jpt2	NO	YES
Ep400	NO	YES
Srrm2	NO	YES
Avil	NO	YES
Rbm48	NO	YES
Sh3d19	NO	YES
Iars1	NO	YES
Baiap2	NO	YES
Safb2	NO	YES
Paics	NO	YES
Slc9a3r1	NO	YES
Aatf	NO	YES
Gpkow	NO	YES
Morc4	NO	YES
Rbm26	NO	YES

Tmsb10	NO	YES
Mafg	NO	YES
Pus1	NO	YES
Oxct1	NO	YES
Pafah1b1	NO	YES
Ppil2	NO	YES
Med1	NO	YES
Aldh18a1	NO	YES
Ppp1r14a	NO	YES
Zcchc10	NO	YES
Supt5h	NO	YES
Gtf3c4	NO	YES
Zfhx3	NO	YES
Ints3	NO	YES
Msh2	NO	YES
Rnf114	NO	YES
Magohb	NO	YES
Hist2h2bb	NO	YES
Pdcd11	NO	YES
Mcrs1	NO	YES
Arhgap5	NO	YES
Ccdc174	NO	YES
Mta1	NO	YES
Xrn2	NO	YES
Chmp1a	NO	YES
Myo5a	NO	YES
Prpf6	NO	YES
Kdm2b	NO	YES
Bap18	NO	YES
Kin	NO	YES
Foxj3	NO	YES
Trmt2a	NO	YES
Arhgef1	NO	YES
Lin9	NO	YES
Tcea1	NO	YES
Dnmt1	NO	YES
Msl2	NO	YES
Gls	NO	YES
Fasn	NO	YES
Uap1l1	NO	YES
Bud13	NO	YES
Rpp38	NO	YES
Map2k4	NO	YES
Znf609	NO	YES
Sart3	NO	YES
Crnkl1	NO	YES
Nfib	NO	YES
Ccdc130	NO	YES

Ppp1r12a	NO	YES
Znf787	NO	YES
Ncbp2	NO	YES
Smtn	NO	YES
Mecom	NO	YES
Brpf1	NO	YES
Plrg1	NO	YES
Rpap3	NO	YES
Prrc2c	NO	YES
Cmtr1	NO	YES
Nsun2	NO	YES
Psmc2	NO	YES
Ppp1r2	NO	YES
Leng8	NO	YES
Nfatc3	NO	YES
Cdyl2	NO	YES
Ccdc124	NO	YES
Eif3b	NO	YES
Pds5b	NO	YES
Cul4b	NO	YES
Ahctf1	NO	YES
Fra10ac1	NO	YES
Arid1b	NO	YES
Cpsf2	NO	YES
Bcorl1	NO	YES
Chaf1a	NO	YES
Eif4g2	NO	YES
Sumo3	NO	YES
Smc5	NO	YES
Stk4	NO	YES
Pdx1	NO	YES
Gins3	NO	YES
Snx5	NO	YES
Cd2bp2	NO	YES
Ccdc6	NO	YES
Khdc4	NO	YES
Bicral	NO	YES
Ehmt2	NO	YES
Prpf4b	NO	YES
Pcgf2	NO	YES
Supt16h	NO	YES
Fignl1	NO	YES
Anp32a	NO	YES
Aldh9a1	NO	YES
Rangap1	NO	YES
Nfic	NO	YES
Abcf2	NO	YES
Lsr	NO	YES

Heatr3	NO	YES
Spart	NO	YES
Rbm8a	NO	YES
Cavin1	NO	YES
Ckap5	NO	YES
Tppp3	NO	YES
Chmp3	NO	YES
Fzd7	NO	YES
Ccnt1	NO	YES
Iws1	NO	YES
Zmym2	NO	YES
Atrx	NO	YES
Fnbp4	NO	YES
Skt	NO	YES
Sgo2	NO	YES
Csnk1a1	NO	YES
Pinx1	NO	YES
Phf23	NO	YES
Cox4i1	NO	YES
Set	NO	YES
Pck2	NO	YES
Slc25a1	NO	YES
Rbm33	NO	YES
Mecr	NO	YES
Gtf3c2	NO	YES
Arl14ep	NO	YES
Zbtb7a	NO	YES
Parp3	NO	YES
Pkn1	NO	YES
Aff4	NO	YES
Mlh1	NO	YES
Mcu	NO	YES
Brd2	NO	YES
Necap1	NO	YES
Gtf2h1	NO	YES
Dis3	NO	YES
Cwc27	NO	YES
Anxa3	NO	YES
Etv6	NO	YES
Ddx11	NO	YES
Isy1	NO	YES
Ubox5	NO	YES
Usp3	NO	YES
Znf638	NO	YES
Inppl1	NO	YES
Snx6	NO	YES
Pcbp1	NO	YES
Mthfd1	NO	YES

Zhx3	NO	YES
Ppp4r3b	NO	YES
Napg	NO	YES
Ppl	NO	YES
Adnp	NO	YES
Cwc15	NO	YES
Cuedc2	NO	YES
Pkn2	NO	YES
Cnbp	NO	YES
Rai1	NO	YES
Ash2l	NO	YES
Gpsm2	NO	YES
Ddx56	NO	YES
Sh3gl1	NO	YES
Kiaa1522	NO	YES
Phb	NO	YES
Srbd1	NO	YES
Nfil3	NO	YES
Rps23	NO	YES
Mpc2	NO	YES
Mphosph10	NO	YES
Upf1	NO	YES
Rpl8	NO	YES
Mnat1	NO	YES
Tma16	NO	YES
Phc3	NO	YES
Smarcc1	NO	YES
Bin1	NO	YES
Phc2	NO	YES
Cetn2	NO	YES
Brip1	NO	YES
Cavin2	NO	YES
Ddx42	NO	YES
Dhx15	NO	YES
Lonp1	NO	YES
Bcar1	NO	YES
Zmym4	NO	YES
Lig1	NO	YES
Clta	NO	YES
Copz1	NO	YES
Dpf1	NO	YES
Cct4	NO	YES
Arid1a	NO	YES
Chaf1b	NO	YES
Sfmbt1	NO	YES
D8Ertd738e	NO	YES
Zc3h4	NO	YES
Atp5pb	NO	YES

Arcn1	NO	YES
Zc2hc1a	NO	YES
Kpna1	NO	YES
Alkbh3	NO	YES
Tomm70	NO	YES
Ptbp2	NO	YES
Zmat2	NO	YES
Abl2	NO	YES
Arnt	NO	YES
Hadhb	NO	YES
Gtf2e2	NO	YES
Wbp4	NO	YES
Mthfd1l	NO	YES
Nckap1	NO	YES
Mpp1	NO	YES
Actr3	NO	YES
Hat1	NO	YES
Atf7	NO	YES
Ccdc71	NO	YES
Cdc73	NO	YES
Ahdc1	NO	YES
Ubn2	NO	YES
Cic	NO	YES
Ftsj3	NO	YES
Mycbpap	NO	YES
Eif3c	NO	YES
Dnaja3	NO	YES
Ppp1r14b	NO	YES
Mastl	NO	YES
Akt1s1	NO	YES
Nsd2	NO	YES
Telo2	NO	YES
Kif22	NO	YES
Ccdc50	NO	YES
Elavl1	NO	YES
Asap1	NO	YES
Ttc33	NO	YES
Znf280c	NO	YES
Kmt2d	NO	YES
Cops4	NO	YES
Pnpt1	NO	YES
Ttf2	NO	YES
Sin3a	NO	YES
Kif2a	NO	YES
Brwd1	NO	YES
Sqor	NO	YES
Socs5	NO	YES
Fkbp5	NO	YES

Pwwp2b	NO	YES
Mtmr12	NO	YES
Sf3a3	NO	YES
Usp47	NO	YES
Rpl10	NO	YES
Btf3	NO	YES
Hnrnpa2b1	NO	YES
Alyref	NO	YES
Krt8	NO	YES
Rrp9	NO	YES
Ikzf5	NO	YES
Bola1	NO	YES
Vps25	NO	YES
Tsc22d4	NO	YES
Nop56	NO	YES
S100a6	NO	YES
Arglu1	NO	YES
Rrp1b	NO	YES
Foxa2	NO	YES
Camsap3	NO	YES
Igfn1	NO	YES
Rtf2	NO	YES
Lrrfip1	NO	YES
Rpf2	NO	YES
Esyt3	NO	YES
Cul1	NO	YES
Prrc2a	NO	YES
Chd8	NO	YES
LRWD1	NO	YES
Cir1	NO	YES
Hnrnpa0	NO	YES
Mrto4	NO	YES
Champ1	NO	YES
Nsd3	NO	YES
Eif3a	NO	YES
Nbn	NO	YES
Myef2	NO	YES
Snrpb2	NO	YES
Rngtt	NO	YES
Gfm1	NO	YES
Kdm2a	NO	YES
Wiz	NO	YES
Ampd2	NO	YES
Vsx2	NO	YES
Acsbg1	NO	YES
Dcaf13	NO	YES
Mrpl12	NO	YES
Phldb2	NO	YES

Fnbp1l	NO	YES
Isg20l2	NO	YES
Ythdc1	NO	YES
Top2a	NO	YES
Bahd1	NO	YES
Cwf19l1	NO	YES
Zc3h8	NO	YES
Rad54b	NO	YES
Rfc1	NO	YES
Ccnb1	NO	YES
Msl1	NO	YES
Gapvd1	NO	YES
Zhx2	NO	YES
Fh	NO	YES
Ythdf1	NO	YES
Psmb3	NO	YES
Snrnp40	NO	YES
Esf1	NO	YES
Usp7	NO	YES
Zyx	NO	YES
Prpf19	NO	YES
Necap2	NO	YES
Pgam1	NO	YES
Cnot2	NO	YES
Rpl36a	NO	YES
Ckap2	NO	YES
Cwf19l2	NO	YES
Rybp	NO	YES
Rtkn	NO	YES
Nacc1	NO	YES
Lbr	NO	YES
Krt76	NO	YES
Fosl2	NO	YES
Tom1l1	NO	YES
Acsl5	NO	YES
Tbl1xr1	NO	YES
Cct5	NO	YES
Hnrnpa3	NO	YES
Nrip1	NO	YES
Atp5f1e	NO	YES
Acot13	NO	YES
Eif3h	NO	YES
Atp5pd	NO	YES
Sbno1	NO	YES
Ilkap	NO	YES
Sinhcaf	NO	YES
Kpna3	NO	YES
Hdac4	NO	YES

Bub1b	NO	YES
Cdk13	NO	YES
Erf	NO	YES
Cmss1	NO	YES
Dido1	NO	YES
Ppp1r10	NO	YES
Cks1b	NO	YES
Mad1l1	NO	YES
Cdk12	NO	YES
Elof1	NO	YES
Bccip	NO	YES
Fam50a	NO	YES
Il1rn	NO	YES
Zeb2	NO	YES
Ndufb11	NO	YES
Sod2	NO	YES
Bclaf1	NO	YES
Spag7	NO	YES
Ywhag	NO	YES
Znf706	NO	YES
Mad2l1bp	NO	YES
Soat1	NO	YES
Dync1li2	NO	YES
Tcf20	NO	YES
Pgs1	NO	YES
Arhgdia	NO	YES
Fau	NO	YES
Ranbp1	NO	YES
Abcf3	NO	YES
Txndc9	NO	YES
Fscn1	NO	YES
Hadh	NO	YES
Tpr	NO	YES
Vill	NO	YES
Adss2	NO	YES
Acin1	NO	YES
Tjap1	NO	YES
Zzz3	NO	YES
Pias4	NO	YES
Fbrs	NO	YES
Arfip1	NO	YES
Slu7	NO	YES
Tbcd	NO	YES
Klf5	NO	YES
Baz1b	NO	YES
Rbm10	NO	YES
Cd2ap	NO	YES
Arhgap1	NO	YES

Etf1	NO	YES
Ctcf	NO	YES
Polr2a	NO	YES
Ppme1	NO	YES
Cbx2	NO	YES
Ess2	NO	YES
Mdc1	NO	YES
Cep131	NO	YES
Crebbp	NO	YES
Serf2	NO	YES
Armc1	NO	YES
Zc3h11a	NO	YES
Acad9	NO	YES
Wdr12	NO	YES
Prpf38b	NO	YES
Washc2	NO	YES
Rsl24d1	NO	YES
Eci1	NO	YES
Cep135	NO	YES
Chmp2b	NO	YES
Syncrip	NO	YES
Pin4	NO	YES
Dlgap5	NO	YES
Dpf2	NO	YES
Rpl10a	NO	YES
Gabpa	NO	YES
Jun	NO	YES
Mettl3	NO	YES
Tnks1bp1	NO	YES
Otud7b	NO	YES
Brf1	NO	YES
Tshz2	NO	YES
Tbcb	NO	YES
Sfswap	NO	YES
Pitrm1	NO	YES
Znf281	NO	YES
Eif3e	NO	YES
Med27	NO	YES
Maea	NO	YES
Esd	NO	YES
Sbds	NO	YES
Faim	NO	YES
Cit	NO	YES
Smyd5	NO	YES
Ak2	NO	YES
Ppp1r18	NO	YES
Ddx10	NO	YES
Vrk3	NO	YES

Ints8	NO	YES
Kiaa0754	NO	YES
Phf21a	NO	YES
Ect2	NO	YES
Marcksl1	NO	YES
Sugp1	NO	YES
Ogfod1	NO	YES
Cbx1	NO	YES
Ptpn2	NO	YES
Cdc5l	NO	YES
Cwc25	NO	YES
Ube2c	NO	YES
Mycbp	NO	YES
Tlk2	NO	YES
Fam172a	NO	YES
Ube2e3	NO	YES
Zc3h15	NO	YES
Psme3ip1	NO	YES
Ddx3x	NO	YES
Samhd1	NO	YES
Limd2	NO	YES
Wdr33	NO	YES
Aifm1	NO	YES
Ykt6	NO	YES
Smc3	NO	YES
Eif2s1	NO	YES
Ep300	NO	YES
Morf4l1	NO	YES
Ncoa3	NO	YES
Dnttip2	NO	YES
Lrpprc	NO	YES
Rnf213	NO	YES
Xpo5	NO	YES
Cdc20	NO	YES
Cks2	NO	YES
Tprkb	NO	YES
Fubp1	NO	YES
Ccdc92	NO	YES
Cdc26	NO	YES
Sap130	NO	YES
Nfatc1	NO	YES
Pacs1	NO	YES
Rbmxl1	NO	YES
Hdac7	NO	YES
Foxp4	NO	YES
C1d	NO	YES
Eya3	NO	YES
Dazap1	NO	YES

Ddx39a	NO	YES
Ston1	NO	YES
Eed	NO	YES
Pcna	NO	YES
Nop2	NO	YES
Zc3h18	NO	YES
Timm44	NO	YES
Hirip3	NO	YES
Gtf2f2	NO	YES
Ube2m	NO	YES
Tfip11	NO	YES
Kat8	NO	YES
Rrp15	NO	YES
Sf3a2	NO	YES
Rcor1	NO	YES
Arhgef18	NO	YES
Psmd12	NO	YES
Nob1	NO	YES
Zfyve19	NO	YES
Ddx5	NO	YES
Noc2l	NO	YES
Anln	NO	YES
Foxp1	NO	YES
Nmt2	NO	YES
Clasrp	NO	YES
Vwa5a	NO	YES
Erc1	NO	YES
Rpl13	NO	YES
Papola	NO	YES
Coro7	NO	YES
Pelp1	NO	YES
Tuba1a	NO	YES
Cdadc1	NO	YES
Ss18	NO	YES
Larp1	NO	YES
U2af2	NO	YES
Mlkl	NO	YES
Rap1gap2	NO	YES
Ppig	NO	YES
Atn1	NO	YES
Txnrd3	NO	YES
Gps2	NO	YES
Smarcc2	NO	YES
Gpatch11	NO	YES
Hnrnpdl	NO	YES
Cpox	NO	YES
Tex10	NO	YES
Aurka	NO	YES

Cs	NO	YES
Crtc2	NO	YES
Setd2	NO	YES
Ahsa1	NO	YES
Psip1	NO	YES
Vcpip1	NO	YES
Atxn1l	NO	YES
Ube3c	NO	YES
Phf5a	NO	YES
Ppan	NO	YES
Anxa1	NO	YES
Cep43	NO	YES
Tjp2	NO	YES
Crtc3	NO	YES
Nsrp1	NO	YES
Zc3hav1	NO	YES
Cpsf4	NO	YES
Akap8	NO	YES
Papss1	NO	YES
Rps8	NO	YES
Igf1	NO	YES
Psmd11	NO	YES
Polr1g	NO	YES
Ppp2r2a	NO	YES
H1-3	NO	YES
Tox4	NO	YES
Rsrc2	NO	YES
Rpl11	NO	YES
Dut	NO	YES
Arhgap27	NO	YES
Fxr2	NO	YES
Nosip	NO	YES
Cdc37	NO	YES
Hspbap1	NO	YES
Atxn2l	NO	YES
Atad3	NO	YES
Banp	NO	YES
Trim33	NO	YES
Ppid	NO	YES
Mta3	NO	YES
Sprr1a	NO	YES
Zc3h14	NO	YES
Stxbp2	NO	YES
Ell	NO	YES
Cnot1	NO	YES
Ercc1	NO	YES
Otud4	NO	YES
Arhgap12	NO	YES

Ywhaq	NO	YES
Drg2	NO	YES
Rbm15	NO	YES
Rad17	NO	YES
Atp5f1d	NO	YES
Tsnax	NO	YES
Pdcl3	NO	YES
Mbd2	NO	YES
Mettl16	NO	YES
Nedd4	NO	YES
Ubap2	NO	YES
Yeats2	NO	YES
Smndc1	NO	YES
Ncaph	NO	YES
Ankrd17	NO	YES
Map2k6	NO	YES
Ncoa2	NO	YES
Fundc2	NO	YES
Baz1a	NO	YES
Znf148	NO	YES
Bcl3	NO	YES
Nol12	NO	YES
Itpr3	NO	YES
Ctdspl2	NO	YES
Spdl1	NO	YES
Hist1h2bp	NO	YES
Taf1	NO	YES
Wdr46	NO	YES
Gse1	NO	YES
Setdb1	NO	YES
Yju2	NO	YES
Fkbp4	NO	YES
Chtop	NO	YES
Nck1	NO	YES

Supplementary Table 4 Clinical information of cohort 4

Number	Gender	Age	Catogory	OS
1			RPC	8.6
2	Male	71	RPC	30.7667
3	Female	72	RPC	26.2667
4	Male	73	RPC	16.9
5	Male	70	RPC	26.2
6			RPC	6.73333
7	Female	75	RPC	6.5
8	Female	70	RPC	5.8
9	Male	64	RPC	15.2667
10	Male	64	RPC	3.53333
11	Female	76	RPC	17.1333
12	Male	74	RPC	21.6
13	Male	69	RPC	7.1
14	Male	58	RPC	17.8667
15	Female	66	RPC	13.1667
16	Male	71	RPC	14.2333
17	Male	83	RPC	10.2667
18	Female	73	RPC	30
19	Male	79	RPC	23.4667
20	Female	62	RPC	25.9667
21	Female	67	RPC	25.6667
22	Female	49	RPC	28.0667
23	Female	65	RPC	11.8333
24	Female	64	RPC	14.6
25	Male	55	RPC	31.0333
26	Male	72	RPC	24.4333
27	Female	74	RPC	13
28	Male	63	RPC	13.8333
29	Male	64	RPC	9.76667
30	Male	79	RPC	3.13333
31	Male	72	RPC	32.7333
32	Female	62	RPC	34.6667
33	Male	63	RPC	30.4333
34	Male	62	RPC	17.2
35	Female	82	RPC	14.4667
36			RPC	13.9667

Supplementary Table 3 Clinical information of cohort 3

Number	Age	Gender	Catogory	Pathology	Treatment	Response	OS	Status
1	61	Female	MPC	No biopsy performed	mFFX+Sintilimab	PR	1044	DEAD
2	67	Male	MPC	Cancer found in biospy	mFFX+Sintilimab	PR	491	DEAD
3	66	Male	MPC	Cancer found in biospy	mFFX+Sintilimab	SD	369	DEAD
4	73	Male	MPC	No biopsy performed	mFFX+Sintilimab	PR	150	DEAD
5	64	Male	MPC	No biopsy performed	mFFX+Sintilimab	SD	272	DEAD
6	75	Male	MPC	Cancer found in biospy	mFFX+Sintilimab	PR	717	DEAD
7		Male	MPC	Cancer found in biospy	mFFX+Sintilimab	PR	273	DEAD
8	79	Male	MPC	Cancer found in biospy	mFFX+Sintilimab	PR	212	DEAD
9	71	Male	MPC	Cancer found in biospy	mFFX+Sintilimab	PR	878	DEAD
10	64	Male	MPC	Cancer found in biospy	mFFX+Sintilimab	PD	150	DEAD
11	59	Male	MPC	No cancer found in biospy	mFFX+Sintilimab	PR	1341	LIVE
12		Male	MPC	No biopsy performed	mFFX+Sintilimab	PD	339	DEAD
13	67	Female	MPC	Cancer found in biospy	mFFX+Sintilimab	PR	983	LIVE
14	74	Male	MPC	Cancer found in biospy	mFFX+Sintilimab	SD	304	DEAD
15	58	Male	MPC	Cancer found in biospy	mFFX+Sintilimab	PD	607	DEAD
16	58	Male	MPC	Cancer found in biospy	mFFX+Sintilimab	SD	593	DEAD
17	68	Male	MPC	No biopsy performed	mFFX+Sintilimab	PD	252	DEAD
18		Male	MPC	No biopsy performed	mFFX+Sintilimab	PD	157	DEAD
19	57	Male	MPC	Cancer found in biospy	mFFX+Sintilimab	PR	592	DEAD
20	59	Male	MPC	No biopsy performed	mFFX+Sintilimab	PR	1230	LIVE
21		Male	MPC	No biopsy performed	mFFX+Sintilimab	PR	356	DEAD
22	73	Male	MPC	No biopsy performed	mFFX+Sintilimab	PR	1181	LIVE
23	68	Male	MPC	Cancer found in biospy	mFFX+Sintilimab	SD	116	DEAD
24	56	Male	MPC	Cancer found in biospy	mFFX+Sintilimab	PD	201	DEAD
25	48	Female	MPC	No cancer found in biospy	mFFX+Sintilimab	CR	890	DEAD
26	61	Female	MPC	Cancer found in biospy	mFFX+Sintilimab	SD	965	LIVE
27	64	Male	MPC	Cancer found in biospy	mFFX+Sintilimab	SD	245	DEAD
28	71	Female	MPC	Cancer found in biospy	mFFX+Sintilimab	SD	177	DEAD
29	72	Male	MPC	Cancer found in biospy	mFFX+Sintilimab	SD	168	DEAD
30	64	Female	MPC	Cancer found in biospy	mFFX+Sintilimab	PR	1077	LIVE
31	69	Male	MPC	Cancer found in biospy	mFFX+Sintilimab	PR	541	DEAD
32	73	Female	MPC	Cancer found in biospy	mFFX+Sintilimab	SD	389	DEAD
33	65	Male	MPC	Cancer found in biospy	mFFX+Sintilimab	PD	253	DEAD
34	62	Male	MPC	Cancer found in biospy	mFFX+Sintilimab	SD	336	DEAD
35	58	Male	MPC	No biopsy performed	mFFX+Sintilimab	PR	308	DEAD
36	59	Male	MPC	Cancer found in biospy	mFFX+Sintilimab	SD	326	DEAD
37	61	Male	MPC	Cancer found in biospy	mFFX+Sintilimab	PR	720	DEAD
38	58	Female	MPC	No biopsy performed	mFFX+Sintilimab	SD	344	DEAD
39	59	Male	MPC	Cancer found in biospy	mFFX+Sintilimab	PR	519	DEAD
40	66	Female	MPC	Cancer found in biospy	mFFX+Sintilimab	PR	928	DEAD
41	67	Male	MPC	Cancer found in biospy	mFFX+Sintilimab	PR	943	LIVE
42	50	Male	MPC	Cancer found in biospy	mFFX+Sintilimab	SD	198	DEAD
43	62	Female	MPC	Cancer found in biospy	mFFX+Sintilimab	SD	130	DEAD
44	52	Male	MPC	Cancer found in biospy	mFFX+Sintilimab	PR	269	DEAD

Supplementary Table 2 Clinical information of cohort 2

Number	Gender	Age	Height	Weight	BMI	T stage	ca199	group	PFS	status
1	Male	55	173	75	25.0593	4	77.7	high	2.5	dead
2	Female	72	160	52	20.3125	1	167.4	high	7.5	dead
3	Male	75	168	60	21.2585	2	2	high	1	dead
4	Male	69	166	70	25.4028	3	730.4	high	6.5	dead
5	Female	64	150	40	17.7778	4	1084.85	high	3	dead
6	Female	67	160	62	24.2188	3	2	high	10.5	dead
7	Male	55	176	55.5	17.9171	4	927.2	high	11	dead
8	Female	64	150	49	21.7778	4	32.43	high	2.5	dead
9	Male	60	173	57	19.0451	1	2	high	9.5	dead
10	Female	70	152	53.5	23.1562	3	2.5	high	15	dead
11	Female	61	160	52	20.3125	2	6588	high	15	live
12	Male	74	168	59	20.9042	3	4617.8	high	3.5	dead
13	Male	66	162	50	19.052	2	8.4	high	5	dead
14	Male	51	170	50	17.301	3	44774.4	high	6	dead
15	Male	69	160	62	24.2188	4	528	high	5.5	dead
16	Female	71	165	52	19.1001	1	2	high	5	dead
17	Male	73	167	62.5	22.4103	4	56.2	high	6	dead
18	Female	71	150	65	28.8889	2	250.2	high	5	dead
19	Male	62	170	65	22.4913	2	124.6	high	11.5	dead
20	Male	70	178	65	20.5151	2	403	high	3.5	dead
21	Female	76	160	53	20.7031	2	3498.91	high	4	dead
22	Male	68	170	63	21.7993	2	148	high	2	dead
23	Male	69	164	83	30.8596	3	1044.8	high	8.5	dead
24	Male	57	172	69	23.3234	4	1046.5	high	3	dead
25	Male	58	173	59	19.7133	3	275.3	high	7	dead
26	Male	54	174	65	21.4692	2	3696.8	low	9.5	dead
27	Male	63	168	51.5	18.2469	3	8570.6	low	3.5	dead
28	Male	71	168	62.8	22.2506	4	243.3	low	16	dead
29	Male	73	169	69	24.1588	4	1468	low	11	dead
30	Male	73	164	48	17.8465	2	3618	low	2	dead
31	Male	72	170	60	20.7612	4	16.7	low	10.5	dead
32	Male	73	170	73	25.2595	2	281.36	low	14.5	dead
33	Male	40	162	51	19.433	4	315.5	low	9.5	dead
34	Male	61	168	69	24.4473	2	367.3	low	16	live
35	Female	75	145	43	20.4518	2	2.3	low	11.5	dead
36	Male	56	171	65	22.2291	3	1859.3	low	15	live
37	Female	69	172	51	17.239	2	2	low	23	live
38	Male	60	173	79	26.3958	4	2	low	3	dead
39	Male	53	170	60	20.7612	4	5634.2	low	7	dead
40	Male	68	178	70	22.0932	4	2987	low	8	dead
41	Male	74	170	58.5	20.2422	1	550.7	low	8	dead
42	Male	65	171	68	23.255	1	82.52	low	21	live
43	Female	63	151	52	22.806	1	406.7	low	16	live
44	Female	60	160	48	18.75	3	18.6	low	4	dead
45	Male	65	173	61	20.3816	4	332.7	low	12	dead
46	Female	[illegible]	[illegible]	[illegible]	[illegible]	[illegible]	[illegible]	low	[illegible]	dead

47	Male	64	178	80	25.2493	1	25.5	low	23	live
48	Female	61	155	45	18.7305	4	348.86	low	16	live
49	Female	67	159	45	17.7999	3	15.4	low	14	dead
50	Female	60	165	62.5	22.9568	3	2.6	low	8	dead
51	Male	60	168	54	19.1327	2	5548.69	low	2	dead

	High uptake (SUV>8.1, n=25)	High uptake (SUV≤8.1, n=26)	p value
mean age, years (SD)	65.88 (6.89)	64.38 (8.17)	0.65
sex, n(%)			0.771
Male	16	18	
Female	9	8	
BMI	21.90 (3.29)	21.35 (2.56)	0.487
mean serum CA19-9 U/mL	2655.78	1415.24	0.171
T stage			0.887
T1	3	4	
T2	8	8	
T3	7	5	
T4	7	9	

Supplementary Table 1 Clinical information of cohort 1

Number	Gender	Age	Catogory	Surgery date
1	Male	70	RPC	2024.4.11
2	Female	64	RPC	2024.4.12
3	Female	50	RPC	2024.4.13
4	Male	66	RPC	2024.4.15
5	Male	80	RPC	2024.4.16
6	Female	74	RPC	2024.4.16
7	Male	77	RPC	2024.4.18
8	Male	59	RPC	2024.4.18
9	Male	76	RPC	2024.4.22
10	Female	55	RPC	2024.4.23
11	Male	62	RPC	2024.4.24
12	Female	77	RPC	2024.4.30
13	Male	50	RPC	2024.5.1
14	Male	38	RPC	2024.5.2
15	Female	70	RPC	2024.5.7
16	Male	74	RPC	2024.5.8
17	Male	61	RPC	2024.5.9
18	Male	64	RPC	2024.5.9
19	Male	59	RPC	2024.5.10
20	Female	47	RPC	2024.5.13
